# Birth Intervals and Health in Adulthood: A Comparison of Siblings Using Swedish Register Data

**DOI:** 10.1007/s13524-018-0673-8

**Published:** 2018-05-21

**Authors:** Kieron J. Barclay, Martin Kolk

**Affiliations:** 1 Max Planck Institute for Demographic Research, Konrad-Zuse-Straße 1, 18057 Rostock, Germany; 2 Department of Social Policy, London School of Economics and Political Science, London, UK; 3 Demography Unit, Department of Sociology, Stockholm University, Stockholm, Sweden; 4 Centre for the Study of Cultural Evolution, Stockholm University, Stockholm, Sweden; 5 Institute for Futures Studies, Stockholm, Sweden

**Keywords:** Birth intervals, Health, Mortality, Population register data, Sweden

## Abstract

A growing body of research has examined whether birth intervals influence perinatal outcomes and child health as well as long-term educational and socioeconomic outcomes. To date, however, very little research has examined whether birth spacing influences long-term health. We use contemporary Swedish population register data to examine the relationship between birth-to-birth intervals and a variety of health outcomes in adulthood: for men, height, physical fitness, and the probability of falling into different body mass index categories; and for men and women, mortality. In models that do not adjust carefully for family background, we find that short and long birth intervals are clearly associated with height, physical fitness, being overweight or obese, and mortality. However, after carefully adjusting for family background using a within-family sibling comparison design, we find that birth spacing is generally not associated with long-term health, although we find that men born after very long birth intervals have a higher probability of being overweight or obese in early adulthood. Overall, we conclude that birth intervals have little independent effect on long-term health outcomes.

**Electronic supplementary material:**

The online version of this article (10.1007/s13524-018-0673-8) contains supplementary material, which is available to authorized users.

## Introduction

Recent years have seen a resurgence of interest in the long-term consequences of fertility decisions for both parents and children. Although several studies have examined how birth order, family size, and parental age at the time of birth are related to long-term cognitive development, educational and socioeconomic attainment, and health (Baranowska-Rataj et al. 2017; Barclay and Kolk 2015; Barclay and Myrskylä 2016; Black et al. 2005; McLanahan 2004), the importance of birth spacing for long-term outcomes has received far less attention. Those studies that have examined the medium- and long-term effects of birth spacing for children have largely focused on educational and socioeconomic outcomes (Barclay and Kolk 2017; Buckles and Munnich 2012; Petterson-Lidbom and Skogman Thoursie 2009; Powell and Steelman 1990, 1993). Although many studies have explored the consequences of birth spacing for child health, studies on the long-term physical health consequences of birth interval length are rare; a study using historical data from China by Campbell and Lee (2009) is the only example that we are aware of. To our knowledge, this question has not been examined in a contemporary setting, which is surprising given that previous research has shown that birth interval length is associated with the risk of preterm birth, low birth weight, and child mortality (Conde-Agudelo et al. 2006; DaVanzo et al. 2008), and poor perinatal outcomes have long-term consequences for socioeconomic attainment (Black et al. 2007; Conley and Bennett 2000) and health (Leon et al. 1998; Moster et al. 2008; Swamy et al. 2008), even in high-income countries. Furthermore, short intervals may increase sibling competition and dilute the time and resources that parents are able to invest in their children (Blake 1989; Zajonc 1976).

In this study, we use Swedish population register data to examine the relationship between birth interval length and height, physical fitness, and the probability of falling into different body mass index (BMI) categories measured at ages 17–20 for men, and mortality over ages 30–74 for both men and women. Our study extends the literature on this topic by examining a range of medium- and long-term health outcomes that have not been previously examined in relation to birth spacing, and we do so using a within-family sibling comparison design that allows us to minimize residual confounding and to isolate the net effect of birth interval length on long-term health. Furthermore, previous research has focused on the length of the birth interval preceding the birth of the index person; in this study, we also examine whether the length of the *subsequent* interval—the time until the birth of a younger sibling—is associated with long-term health.

## Empirical Research on Birth Intervals and Health

Despite the large and growing literature examining the relationship between family background and family structure on long-term health (e.g., Baranowska-Rataj et al. 2017; Elo and Preston 1992; Hayward and Gorman 2004; McEniry 2013; Preston et al. 1998), very little research has examined the long-term effects of birth spacing on the health of the children. A study using historical data from Qing China from 1749–1909 indicated that a short preceding birth interval of less than two years was associated with substantially higher mortality at ages 55–74, and this pattern persists even when comparing siblings within the same family (Campbell and Lee 2009). Some preliminary evidence also suggests that short birth intervals may be associated with an increased risk of schizophrenia (Gunawardana et al. 2011; Smits et al. 2004), autism (Gunnes et al. 2013), and self-harm (Riordan et al. 2012), but few other studies have pursued this topic.

In contrast to the research on the long-term health effects of birth intervals, a voluminous literature has explored the short-term health effects of birth spacing on child and maternal health. Hundreds of studies using data from low-income countries have shown that short birth intervals—variously classified as less than 9 or 24 months—are associated with an increased risk of low birth weight, preterm birth, intrauterine growth restriction (IUGR), and being small for gestational age (SGA) (Conde-Agudelo et al. 2006) as well as fetal, neonatal, and infant mortality (Casterline 1989; Conde-Agudelo et al. 2005; Fortney and Higgins 1984; Huttly et al. 1992; Rutstein 2005; Smith et al. 2003). A meta-analysis of studies using data from both low- and high-income countries published up to 2006 showed a J-shaped curve in the relationship between the length of birth intervals and perinatal and child health outcomes; interpregnancy intervals shorter than 18 months and longer than 59 months are significantly associated with poor perinatal outcomes (Conde-Agudelo et al. 2006). As a consequence, the World Health Organization (WHO) issued recommendations for mothers to wait at least 24 months before attempting to conceive again (WHO 2005).

Evidence on the negative effects of especially short and long birth intervals is not limited to low-income countries. Studies from high-income countries in North America and Western Europe also suggest that short and long birth intervals are associated with an increased risk of preterm birth, low birth weight, SGA (Conde-Agudelo et al. 2006), and to a certain extent also an increased risk of fetal death, neonatal mortality, and infant mortality (Hussaini et al. 2013; McKinney et al. 2017; Stephansson et al. 2003). In this study, we examine health outcomes in adulthood rather than childhood, but a growing number of studies have linked perinatal outcomes to long-term health. Low birth weight and preterm birth are associated with an increased risk of cardiovascular disease (Frankel et al. 1996; Leon et al. 1998; Rich-Edwards et al. 1997); mental and physical disability (Moster et al. 2008); higher mortality (Swamy et al. 2008); lower fertility (Swamy et al. 2008); and lower height, IQ, and educational and socioeconomic attainment (Behrman and Rosenzweig 2004; Black et al. 2007; Conley and Bennett 2000; Derraik et al. 2017). Behrman and Rosenzweig (2004) reported that birth weight is not associated with BMI, but other work has shown that preterm birth is associated with altered adiposity (Uthaya et al. 2005). Children born with low birth weight experience accelerated weight gain during infancy, and research has suggested that this may be linked to an increased risk of being overweight or obese in adulthood (Mathai et al. 2013) as well as a higher risk cardiovascular health profile (Posod et al. 2016). Given that short birth intervals are also associated with worse maternal health (Conde-Agudelo et al. 2007), birth interval length possibly affects the long-term health of the child through a negative effect on the health, and perhaps subsequently socioeconomic status, of the mother. Other work has also indicated that short birth spacing has negative effects on long-term development, as evidenced by lower grades in high school and a lower probability of pursuing tertiary education (e.g., Buckles and Munnich 2012; Powell and Steelman 1990, 1993), which are also known to affect health (Mackenbach et al. 1997; Torssander and Erikson 2010).

Despite this almost overwhelming body of evidence, a pair of recent studies cast doubt on whether the length of birth intervals is causally responsible for poor perinatal outcomes in high-income countries (Klebanoff 2017). Analyses using data from Australia (Ball et al. 2014) and Canada (Hanley et al. 2017) revealed that when siblings born to the same mother are compared, the association between short birth spacing and the risk of preterm birth, low birth weight, and SGA is either completely removed or substantially reduced. However, another pair of recent studies that also used a sibling-comparison design found that the association between short intervals and an increased risk of preterm birth and low birth weight persisted (Koullali et al. 2017; Shachar et al. 2016). Partly in response to these new findings, a 2015 report from the Centers for Disease Control and Prevention (CDC) in the United States suggested that more research is needed to understand the effect of birth spacing on maternal and child health (Copen et al. 2015).

## Birth Intervals and Long-Term Health: Potential Explanatory Mechanisms

A previous review by Conde-Agudelo et al. (2012) of potential explanatory mechanisms for a causal relationship between birth interval length and child health outcomes identified eight candidates: maternal nutrient depletion, folate depletion, cervical insufficiency, vertical transmission of infections, suboptimal lactation related to breastfeeding-pregnancy overlap, physiological regression, sibling competition, and transmission of infectious diseases among siblings. The first six can be broadly categorized as physiological explanations related to prenatal conditions, while the latter two are better categorized in reference to social and environmental conditions within the family and household. Another important factor is the role of confounding and selection processes. In the following sections, we consider each of these three groups of explanations (physiological, social/environmental, selection/confounding) in turn, and discuss the processes by which each might be linked to the long-run health outcomes that we study in this outcome: height, physical fitness, BMI, and mortality.

### Physiological Mechanisms

The *maternal nutrient depletion hypothesis*, in relation to birth intervals, describes how the health of the mother as well as the fetus can be affected if the mother suffers from nutrient depletion because of a short interval between pregnancies (King 2003; Winkvist et al. 1992). Essentially, a short birth interval and postpartum activities, such as breastfeeding, mean that the mother may not have completed the process of nutrient repletion, which can lead to competition between the mother and fetus for resources, thereby affecting fetal growth. The *folate depletion hypothesis* is very similar but applies specifically to the maternal repletion of folic acid, which is critical for fetal growth (Smits and Essed 2001). The *cervical insufficiency hypothesis* describes how insufficient time between pregnancies can mean that muscles in the reproductive tissues do not fully recover, limiting the mother’s physical ability to retain the pregnancy (Haaga 1988). Structural weaknesses in the cervix can lead to preterm birth (Ludmir and Sehdev 2000). The *vertical transmission of infections hypothesis* concerns how pregnant women can contract infections during pregnancy, which may continue to survive in or on their bodies for a limited period after giving birth (Goldenberg et al. 2005), increasing the risk of exposure for a fetus conceived after a short interval. These persistent maternal infections may be located in a physical region whereby the new fetus can be directly infected or cause an infection that leads to preterm delivery (Cheng et al. 2008). These hypotheses primarily predict poor outcomes for the pregnancy following a short birth interval.

The *breastfeeding-pregnancy overlap hypothesis* describes how continued breastfeeding during a subsequent pregnancy, which is relatively uncommon because of lactational amenorrhea (Trussell 2004) but is not impossible, can lead to lower quality breastmilk given the competing demands on the mother’s physical resources. However, this behavior is probably very uncommon in Sweden. Breastfeeding-pregnancy overlap would primarily affect the older child whose younger sibling was conceived after a short interval. The *physiological regression hypothesis* has been proposed to explain why relatively long birth intervals—greater than five years—are also associated with worse perinatal outcomes (Zhu et al. 1999). Pregnancy is associated with a number of physical changes in the female body, which first occur during a woman’s first pregnancy. A long birth interval may lead to a physical regression, a state in which the body is no longer primed for childbearing. It has been suggested that this is why perinatal outcomes for children born after long birth intervals are similarly poor to perinatal outcomes for firstborn children (Zhu et al. 1999).

Reviewing the research, Conde-Agudelo et al. (2012) found only indirect evidence to support the maternal depletion and physiological regression hypotheses, growing evidence to support the folate depletion hypothesis, emerging evidence to support the vertical transmission of infections and cervical insufficiency hypotheses, and only limited evidence to support the breastfeeding-pregnancy overlap hypothesis.

### Social and Environmental Mechanisms

Birth interval length may also influence social and environmental conditions during childhood, by diluting parental resources and by affecting interaction dynamics between siblings. The *sibling competition*—or *resource dilution*—*hypothesis* (Blake 1989) can be applied to birth spacing when one considers how short intervals mean that parental resources are split among their children, particularly during the earliest years of life, which recent research has suggested may be a particularly sensitive development period (Campbell and Ramey 1994; Campbell et al. 2001; Heckman 2006; Knudsen et al. 2006). Even though the dilution of socioeconomic resources may not have a large effect in Sweden given the high level of human development, comprehensive welfare state, and extensive parental leave system, parental *time* is absolutely finite. Some preliminary evidence indicates that short birth intervals lead to less parental supervision in disadvantaged families (Crowne et al. 2012) and that intervals shorter than two years are associated with an increased risk of injuries among children (Nathens et al. 2000).

Another potentially important theory here is the *confluence hypothesis* (Zajonc and Markus 1975), which argues that short birth intervals mean that the average degree of intellectual stimulation in the household is more rapidly lowered by the arrival of an additional child. Thus, for previous siblings, short birth intervals mean less time interacting with cognitively mature parents and more time interacting with callow siblings. These processes could lead indirectly to worse health in adulthood by negatively affecting cognitive development and educational and socioeconomic achievement.

The *transmission of infectious disease hypothesis* argues that the younger of any pair of siblings will be more exposed to infectious diseases than they would be without an older sibling, particularly if the older sibling is roughly two years older—a common birth interval length and an age at which the older sibling is commonly carrying an infection (Conde-Agudelo et al. 2012). Alternatively, however, the *hygiene hypothesis* has been proposed to explain why children from larger families are less likely to develop allergies: early-life exposure to disease can strengthen the capacities of the immune system (Karmaus and Botezan 2002; Strachan 1989). Indeed, in a high-income country like Sweden, where disease exposure during childhood is rarely severe or life-threatening, the more common mild respiratory infections that children experience may help to strengthen the immune system. Thus, the transmission of mild infectious diseases might be beneficial for long-term health rather than harmful.

### Selection and Confounding

Although the aforementioned hypothetical mechanisms linking birth intervals to long-term health assume a genuine causal relationship, an alternative, noncausal explanation is that any crude association between birth interval length and long-term health might be explained by confounding by factors related to the timing and spacing of births as well as child health. Such confounding factors might include maternal health, or parental education and socioeconomic conditions. If, for example, especially short or long birth intervals were more common among parents with low education or worse health, this could explain a negative association between birth interval length and long-term child health. For example, in the United States, interpregnancy intervals less than 18 months are more likely to be reported as mistimed or unwanted; however, among those who did report short intervals, those who were older, more highly educated, and married were more likely to report them as intentional (Gemmill and Lindberg 2013). The latter pattern seems to reflect a later age at first birth and an accelerated fertility schedule designed to achieve desired family size (Gemmill and Lindberg 2013). Nevertheless, despite socioeconomic advantage, multiple closely spaced births among mothers of advanced reproductive age may exacerbate the harmful effect of the aforementioned physiological mechanisms. Other factors, such as underlying maternal health, are more difficult to observe and may also be associated with birth interval length. For example, a woman who has difficulty getting pregnant because of an underlying health issue will be more likely to have longer birth intervals; in turn, her health might affect her child-rearing or her children might inherit her health problems.

It is difficult *a priori* to assess the extent to which previous research on birth spacing may have been affected by omitted variable bias. However, recent studies that have examined the association between birth interval length and both short- and long-term outcomes among siblings born to the same mother suggest that this may indeed be a prevalent issue. By comparing siblings born to the same mother, it is possible to adjust for all factors that are shared among siblings but that might otherwise be difficult to observe and adjust for, such as maternal and paternal health, shared genetics, and unmeasured socioeconomic aspects of the shared home environment. As described earlier in the section on previous empirical research, several recent studies have shown that after siblings are compared, the relationship between birth interval length and the risk of poor perinatal outcomes (such as preterm birth, low birth weight, and SGA) is either completely removed or reduced substantially (Ball et al. 2014; Hanley et al. 2017; Shachar et al. 2016). Furthermore, another recent study adopting the same approach (Barclay and Kolk 2017) showed that the previously widely reported negative effect of short birth spacing on medium- and long-term educational, cognitive, and socioeconomic outcomes (e.g., Buckles and Munnich 2012; Powell and Steelman 1990, 1993) is also wiped out.

The existing body of evidence provides good reasons to believe that birth intervals should be related to long-term health outcomes, although a number of recent studies have suggested that this association may be primarily driven by selection and confounding. In this study, we apply a sibling fixed-effects approach to assess the relationship between birth interval length and long-term health.

## Data and Methods

### Data

This study uses administrative register data on the full population of Sweden. Each individual in Sweden has a unique personal identification number (PIN) that is universally used for administrative purposes. A key administrative register that we use in this study is the Swedish multigenerational register, which allows us to link individuals to their parents and siblings. We examine sibling groups where all the children were born in Sweden in order to maximize the accuracy of the parent-child-sibling linkages.

We examine the relationship between birth intervals and five outcome variables: height, physical fitness, being overweight or obese, being underweight or severely underweight, and mortality. Apart from mortality, information on all the other outcome variables is drawn from the Swedish military conscription register. In Sweden, men were universally required to report to military conscription tests between ages 17 and 20 to determine their physical and psychological suitability for military service. Data on height, physical fitness, and BMI are available for cohorts born 1962–1979. Because only men were required to report to conscription tests in Sweden, we do not have data on these measures for women. However, although the outcome measures are available for men only, the measures of birth spacing and other characteristics of the sibling group are based on the whole sibling groups, including males and females.

The other main register that we use is the Swedish mortality register, which contains detailed information on all deaths in Sweden between 1960 and 2012. Although the Swedish mortality register contains data for the period 1960–2012, the multigenerational registers that allow family members to be linked to one another are incomplete before the 1990s (Statistiska Centralbyråns (SCB) 2011). In this study, we focus on all-cause mortality over the period 1990–2012. We study mortality for Swedish men and women born in 1938–1960; thus, we study mortality over the age range 52–74 for those born in 1938 and age range 30–54 for those born in 1960.

This study is based on a population of sibling groups for which neither parent has any children with a third partner. Thus, none of the individuals included in our analysis have any half-siblings. Although this restriction reduces external validity, it also offers distinct advantages, such as increasing the degree of genetic similarity of the siblings and the likelihood that the children share the same childhood environment. We also exclude sibling groups with multiple births because it is not possible to distinguish the effect of birth interval from the presence of a twin on long-term outcomes in these families.

In our analyses, we compare the results from within-family sibling fixed-effects models and between-family linear regression models. We explain those models in detail in the Statistical Analyses section, but this modeling approach also has implications for our data selection. Because the fixed-effects approach requires variance in the groups in which the comparisons are conducted, we necessarily exclude the following: (1) sibling groups with only one child, given the irrelevance of birth spacing in such cases; and (2) sibling groups with two children because these sibling groups contain only one birth interval. Nevertheless, by studying birth intervals in families with three or more children, we still study the majority of birth intervals observed in the population. Sibling groups of size *N* contribute *N* – 1 birth intervals to the total population of birth intervals. Therefore, in a sibling group with four children, three siblings have a younger (or older) sibling; in a sibling group with two children, only one child has a younger (or older) sibling. The four-child group therefore has three times as many intervals as the two-child group. Given the family size distribution in Sweden in the birth cohorts that we study, we estimate that by examining sibling groups with three or more children, we are examining sibling groups where more than 69 % of all birth intervals are observed.

In studying the effect of birth intervals on each of the five outcomes that we address, we perform separate analyses examining the importance of the length of the *preceding birth interval* (the time in months between the birth of the older sibling and the birth of the index person) and the *subsequent birth interval* (the time in months between the birth of the index person and the birth of the younger sibling). In the analyses examining the importance of the preceding birth interval, we necessarily exclude firstborn individuals given that there was no preceding interval. Thus, our analysis population for the analyses on the preceding birth interval is second and later-born children in sibling groups with at least three children. In our analyses examining the importance of the subsequent birth interval, we necessarily exclude last-born individuals because they do not have any siblings born after them. Table S1 in Online Resource 1 details how we reach our analytical sample.

**Fig. 1:**
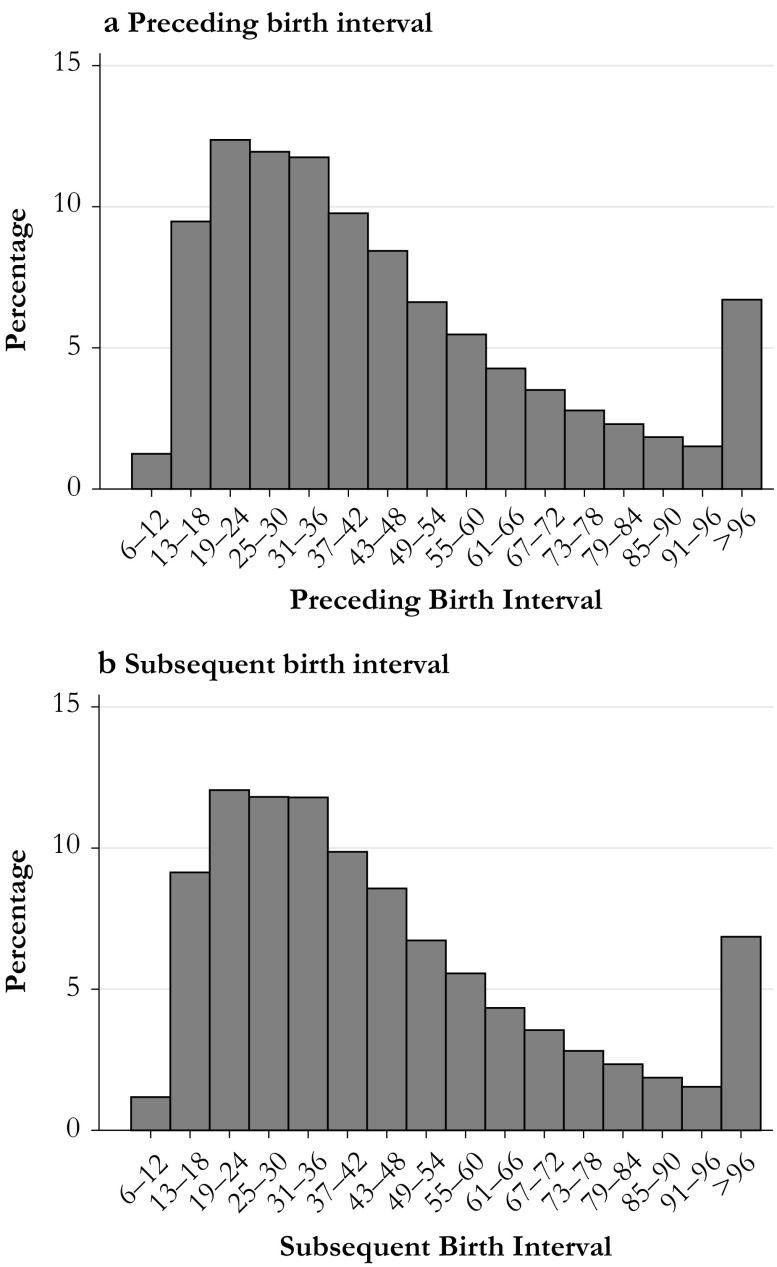
Birth interval (months) distribution in Sweden, 1938–1979

The measure for the birth interval that we use in this study is the length of the birth-to-birth interval, meaning the months between one live birth and another. We categorize the length of the birth interval into 16 categories of six-month periods, ranging from a minimum of 9-12 months to 96 months or longer. The reference category for the preceding and subsequent birth interval is 25–30 months. The distribution of birth intervals in Sweden between 1938 and 1979 is shown in Fig. 1.

### Outcome Variables

#### Height

Height, measured in centimeters, is standardized.

#### Physical Fitness

Our measure for physical fitness is based on a measure of maximal working capacity, measured in watts (*fysisk arbetsförmåga i watt*). *Maximal working capacity* (MWC)— measured as the maximum resistance attained in watts when riding on a stationary bike (one of the most effective ways of measuring aerobic fitness) for 5–10 minutes—is closely related to maximal oxygen uptake (V02max), also known as maximal aerobic capacity. The correlation between these two variables has been reported to be approximately .9 (Patton et al. 1982). The variable for MWC is an important predictor of mortality in adulthood among men (Sandvik et al. 1993). Because a measure of MWC in watts is not intuitively easy to interpret, we standardize this outcome measure.

#### BMI Categories

We calculate BMI as mass (in kg) divided by height (in m) squared at the time of conscription test. Using the standard cutoff points, we categorize BMI as overweight or obese (≥25), normal (18.5–24.9), and underweight or severely underweight (<18.5).

#### Mortality

We study all-cause mortality in the period 1990–2012 for Swedish men and women born in 1938–1960. Thus, conditional on survival to 1990, we study mortality over the age range 52–74 for those born in 1938 and over the age range 30–54 for those born in 1960.

### Covariates

In addition to our main explanatory variable—the length of birth intervals—we include several covariates in our models that are likely to be associated with both birth spacing as well as long-term health. Factors such as birth order, parental age at the time of birth, and birth year may be associated with birth interval length, and are also associated with long-term health outcomes. We include controls for birth order because both the confluence hypothesis and the resource dilution hypothesis predict independent effects of birth order and birth spacing, and previous research has indicated that birth order is related to height (Myrskylä et al. 2013), physical fitness (Barclay and Myrskylä 2014), BMI (Jelenkovic et al. 2013), and mortality (Barclay and Kolk 2015). Birth interval length is also likely to be associated with maternal age, and maternal age is associated with adult height (Barclay and Myrskylä 2016), physical fitness (Barclay and Myrskylä 2016), and mortality (Smith et al. 2009). We adjust for maternal age using five-year categories. Previous studies have also shown secular trends in height, obesity, and mortality, with people becoming taller (Komlos and Lauderdale 2007), heavier (Lissner et al. 2000), and living longer (Oeppen and Vaupel 2002); thus, we also adjust our analyses for birth year. For the analyses drawn from the military conscription register, we also adjust for age at the time of conscription test and the year of the conscription test.

### Statistical Analyses

#### Military Conscription Data

To study the relationship between birth intervals and the four outcome variables drawn from the military conscription register (described earlier), we use fixed-effects linear regression. Our outcome variables for physical fitness and height are continuous, but we use the categories of being overweight/obese and being underweight/severely underweight as binary variables, treating each outcome as though it were independent. In these cases, we also use a linear probability model with robust Huber-White standard errors (Stock and Watson 2008). We prefer linear probability models over nonlinear models, such as the logit specification, because only the former allow direct comparisons of coefficients across models and groups (Mood 2010), and one of the aims of our study is to compare the effect of birth intervals length across the various health outcomes that we study. Average marginal effects from logit models are comparable but are then close to identical to unstandardized coefficients from linear probability models (Angrist and Pischke 2009:103–107). The linear probability model is a consistent estimator even for binary outcomes (Angrist and Pischke 2009:47, 51), our data set is very large, and inference problems related to heteroskedastic residuals in the linear probability model are mitigated by robust standard errors. However, we also run these analyses using a logit model in order to check that the patterns are the same.

The fixed effects are applied to the sibling group; that is, we conduct a within-family comparison. The use of sibling fixed effects implicitly adjusts for all factors that remain constant within the sibling group. Thus, the within-family comparison adjusts for the size of the sibling group, as well as parental resources, to the degree that the latter remains constant. The fixed-effects approach also inherently adjusts for factors that are difficult to observe and measure, such as all elements of shared socioeconomic background and general parenting style, to the extent that such factors are indeed shared by siblings.

For each outcome variable, we estimate four models: (1) one between-family comparison, and (2) one within-family comparison, examining the relationship between the preceding birth interval and the outcome variable; and (3) one between-family comparison, and (4) one within-family comparison examining the relationship between the subsequent birth interval and the outcome variable, using a different population for the analyses on the preceding and subsequent intervals:

1 $$ {y}_i={\upbeta}_1{PBI}_i+{\upbeta}_2{BirthOrder}_i+{\upbeta}_3{MatAge}_i+{\upbeta}_4{BirthYear}_i+{\upbeta}_5{ConAge}_i+{\upbeta}_6{ConYear}_i+{\upbeta}_7{Size}_i+\upalpha +{\upvarepsilon}_i $$

2 $$ {y}_{ij}={\upbeta}_1{PBI}_{ij}+{\upbeta}_2{BirthOrder}_{ij}+{\upbeta}_3{MatAge}_{ij}+{\upbeta}_4{BirthYear}_{ij}+{\upbeta}_5{ConAge}_{ij}+{\upbeta}_6{ConYear}_{ij}+{\upalpha}_j+{\upvarepsilon}_{ij} $$

3 $$ {y}_i={\upbeta}_1{SBI}_i+{\upbeta}_2{BirthOrder}_i+{\upbeta}_3{MatAge}_i+{\upbeta}_4{BirthYear}_i+{\upbeta}_5{ConAge}_i+{\upbeta}_6{ConYear}_i+{\upbeta}_7{Size}_i+\upalpha +{\upvarepsilon}_i $$

4 $$ {y}_{ij}={\upbeta}_1{SBI}_{ij}+{\upbeta}_2{BirthOrder}_{ij}+{\upbeta}_3{MatAge}_{ij}+{\upbeta}_4{BirthYear}_{ij}+{\upbeta}_5{ConAge}_{ij}+{\upbeta}_6{ConYear}_{ij}+{\upalpha}_j+{\upvarepsilon}_{ij}, $$

where *y*_*ij*_ is the outcome for individual *i* in sibling group *j* on height, physical fitness, being overweight or obese, and being underweight or severely underweight. In Model 1, we use a regular linear regression—a between-family comparison—to examine the relationship between the length of the preceding birth interval (*PBI*_*i*_) and the health outcome; we control for birth order, maternal age, birth year, age at the time of the conscription test, year of the conscription test, and sibling group size. *PBI*_*i*_ is entered into the model as a series of 16 dummy variables based on six-month categories for the length of the preceding birth interval. In Model 1, our analysis population is second and later-born children in sibling groups with at least three children; that is, we exclude firstborns because they have no value for the length of the preceding interval. In Model 2, we introduce the sibling fixed effect, α_*j*_, and remove the control for sibling group size because it is adjusted for in the fixed-effects approach. We use the same analysis sample for Model 2 as used in Model 1. Models 3 and 4 follow the same format except that we substitute the variable for the preceding interval with *SBI*, a variable for the length of the subsequent interval. We regard Models 2 and 4 as an improvement on Models 1 and 3, respectively: the sibling comparison approach that we use in Models 2 and 4 minimizes residual confounding from unobserved factors that are shared by siblings. To this end, we are much better able to isolate the net effect of birth intervals on the multiple long-term outcomes that we study.

#### Mortality Data

To study mortality, we use survival analysis in the form of Cox proportional hazard regressions (Cox 1972). The proportional hazards model is expressed as

5 $$ h\left(t\left|{X}_1,\dots, {X}_k\right.\right)={h}_0(t)\exp \left(\sum \limits_{j=1}^k{\upbeta}_j{X}_j(t)\right), $$

where *h*(*t*|*X*_1_, . . . , *X*_*k*_) is the hazard rate for individuals with characteristics *X*_1_, . . . , *X*_*k*_ at time *t*; *h*_0_(*t*) is the baseline hazard at time *t*; and β_*j*_, *j* = 1, . . . , *k* are the estimated coefficients. Because the failure event in our analysis is the death of the individual, the baseline hazard of our model, *h*_0_(*t*), is age. Individuals are censored on first migration out of Sweden, at death, or in 2012—whichever comes first. We estimate robust standard errors to account for clustering within sibling groups (Lin and Wei 1989). To estimate a sibling comparison model, we use stratified Cox models (Allison 2009), stratified by the shared sibling group ID. The stratified Cox model estimating the hazard for an individual from stratum *s* takes the following form:

6 $$ {h}_s\left(t\left|{X}_1,\dots, {X}_k\right.\right)={h}_{0s}(t)\exp \left(\sum \limits_{j=1}^k{\upbeta}_j{X}_j(t)\right), $$

where *h*_0*s*_(*t*) is the baseline hazard for stratum *s*, *s* = 1, . . . , *S*. Each stratum, *s*, is a sibling group. In the standard Cox proportional hazard regression, the baseline hazard *h*_0_ is common to all individuals in the analysis. In the stratified Cox model (Eq. (6)), we allow the baseline hazard to differ between strata, based on the assumption that there are unobserved factors particular to each sibling group that may confound the relationship between birth intervals and mortality in adulthood (Allison 2009: chapter 5). As with the fixed-effects approach applied to linear regression, these stratified Cox models adjust for all factors that are shared by siblings, and we also estimate cluster-adjusted robust standard errors (Lin and Wei 1989). We estimate the following models:

7 $$ \log h(t)={\upbeta}_1{PBI}_i+{\upbeta}_2{Sex}_i+{\upbeta}_3{BirthOrder}_i+{\upbeta}_4{MatAge}_i+{\upbeta}_5{BirthYear}_i+{\upbeta}_6{Size}_i $$

8 $$ \log h(t)={\upbeta}_1{PBI}_{ij}+{\upbeta}_2{Sex}_{ij}+{\upbeta}_3{BirthOrder}_{ij}+{\upbeta}_4{MatAge}_{ij}+{\upbeta}_5{BirthYear}_{ij}+{\upalpha}_j $$

9 $$ \log h(t)={\upbeta}_1{SBI}_i+{\upbeta}_2{Sex}_i+{\upbeta}_3{BirthOrder}_i+{\upbeta}_4{MatAge}_i+{\upbeta}_5{BirthYear}_i+{\upbeta}_6{Size}_i $$

10 $$ \log h(t)={\upbeta}_1{SBI}_{ij}+{\upbeta}_2{Sex}_{ij}+{\upbeta}_3{BirthOrder}_{ij}+{\upbeta}_4{MatAge}_{ij}+{\upbeta}_5{BirthYear}_{ij}+{\upalpha}_j, $$

where log*h*_*i*_(*t*) is the log hazard of mortality, α_*j*_ is the fixed effect for sibling group *j*, and the index *ij* refers to the individual *i* in sibling group *j*. As with the linear regression analyses, *PBI*_*i*_ is entered into the model as a series of 16 dummy variables based on six-month categories for the length of the preceding birth interval. In Model 7, our analysis population is second and later-born children in sibling groups with at least three children; that is, we exclude firstborns because they have no value for the length of the preceding interval. In Model 8, we introduce the sibling fixed effect, α_*j*_, and remove the control for sibling group size because it is implicitly adjusted for. We use the same analysis sample for Model 7 as used in Model 6. In Models 9 and 10, we substitute the variable for the preceding interval with *SBI*_*i*_, a variable for the length of the subsequent interval. We regard Models 8 and 10 as improvements on Models 7 and 9, respectively: the stratified approach that we use in Models 8 and 10 minimizes residual confounding from unobserved factors that are shared by siblings.

## Results

### Descriptive Statistics

Table 1 shows the relative degree of variation in birth interval lengths across and within the families that we study in our analyses. The degree of within-family variation is smaller than the between-family variation in the sample of data drawn from the military conscription registers. However, the degree of variation is still substantial; our within-family models are not based on data with low variation in birth interval length. In the sample that we use to study mortality, the within-family variation in birth interval length is very similar to the variation found across families. The lower within-family variation among men included in the military conscription data is attributable to period declines in within-family variation in birth spacing because the military conscription data are drawn from later birth cohorts.

**Table 1:** Mean and standard deviation of preceding and subsequent birth interval length in months in the analytical samples used for the military conscription data analysis and the mortality data analysis

	Conscription Sample				Mortality Sample			
	Preceding		Subsequent		Preceding		Subsequent	
	Between	Within	Between	Within	Between	Within	Between	Within
Mean	42.6	42.6	47.5	47.5	41.5	41.5	43.6	43.6
SD	22.3	11.8	26.4	16.9	19.7	20.0	23.4	21.7

Table 2 displays summary statistics for the five outcome variables that we study. For physical fitness, height, being underweight or severely underweight, and being overweight or obese, we show the mean by categories of birth interval length in our analytical samples. For mortality, we show the number of deaths by birth interval length as well as the rate of mortality. For physical fitness, those who experienced a preceding or subsequent birth interval of 25–36 months have the highest scores, while both shorter and longer intervals are associated with lower fitness. For height, we observe the same pattern by the length of the subsequent interval but find little variation by the length of the preceding interval. Being underweight is less common at the tail ends of the birth interval distribution, while the percentage of overweight individuals is higher among those born after a long preceding birth interval. Mortality rates are lower among those born after a long interval but higher among those born before a long interval. More detailed descriptive information can be found in Online Resource 1, Tables S2–S5.

**Table 2:** Summary statistics for the outcome variables by the length of preceding and subsequent birth intervals in months

	Physical Fitness (watts)				Height (cm)			
	Preceding		Subsequent		Preceding		Subsequent	
Interval	Mean	SD	Mean	SD	Mean	SD	Mean	SD
9–12	295.1	50.4	294.4	52.7	178.8	6.6	178.6	6.6
13–18	297.0	51.7	298.5	52.6	179.3	6.5	179.4	6.6
19–24	299.6	52.1	301.7	51.2	179.5	6.5	179.8	6.5
25–30	301.4	51.4	302.2	51.3	179.5	6.4	179.6	6.5
31–36	300.9	51.3	302.3	51.4	179.5	6.5	179.6	6.5
37–42	299.9	50.7	301.5	51.3	179.5	6.5	179.5	6.5
43–48	299.0	51.1	302.4	51.2	179.5	6.4	179.6	6.5
49–54	299.1	51.6	301.4	50.8	179.6	6.5	179.5	6.4
55–60	298.3	50.4	299.8	51.0	179.5	6.5	179.5	6.5
61–66	298.6	50.1	301.5	52.0	179.5	6.4	179.4	6.5
67–72	298.9	52.0	300.8	50.9	179.7	6.6	179.4	6.5
73–78	299.1	50.8	302.3	50.7	179.6	6.6	179.4	6.5
79–84	298.1	51.6	301.5	52.5	179.4	6.7	179.3	6.6
85–90	297.8	49.0	299.5	51.6	179.6	6.2	179.5	6.5
91–96	299.4	49.9	299.4	49.1	179.9	6.5	179.1	6.5
97+	297.5	51.6	299.7	50.5	179.5	6.5	179.3	6.4
Total	299.4	51.3	301.2	51.3	179.5	6.5	179.5	6.5
	Underweight		Overweight		Mortality			
	Preceding	Subsequent	Preceding	Subsequent	Preceding		Subsequent	
Interval	%	%	%	%	Rate (10^–3^)	Deaths	Rate (10^–3^)	Deaths
9–12	6.4	6.8	9.5	11.7	1.22	659	1.25	624
13–18	7.5	7.2	10.2	10.9	1.20	6,322	1.32	6,352
19–24	6.5	7.3	9.9	10.3	1.18	7,366	1.37	7,658
25–30	6.8	7.1	9.8	10.8	1.25	6,230	1.41	6,287
31–36	6.9	7.1	11.0	10.5	1.23	5,038	1.46	5,374
37–42	7.1	7.3	11.0	10.9	1.24	3,982	1.49	4,337
43–48	6.4	7.3	11.3	10.8	1.21	3,269	1.56	3,758
49–54	7.3	7.0	11.5	11.2	1.22	2,586	1.52	2,943
55–60	6.7	7.0	13.0	10.7	1.12	2,041	1.60	2,606
61–66	5.9	6.8	13.2	10.8	1.16	1,659	1.61	2,125
67–72	7.3	6.7	13.9	10.3	1.16	1,349	1.59	1,689
73–78	7.9	6.1	14.1	11.4	1.09	1,026	1.58	1,398
79–84	6.7	6.6	15.3	10.4	1.06	822	1.64	1,215
85–90	6.1	7.4	15.4	11.3	1.00	596	1.66	980
91–96	4.5	6.5	17.1	10.8	0.95	477	1.63	843
97+	6.0	6.3	18.0	11.1	0.84	1,635	1.68	3,820
Total	6.8	7.0	11.5	10.8	1.18	45,057	1.47	52,009

### Height

**Fig. 2:**
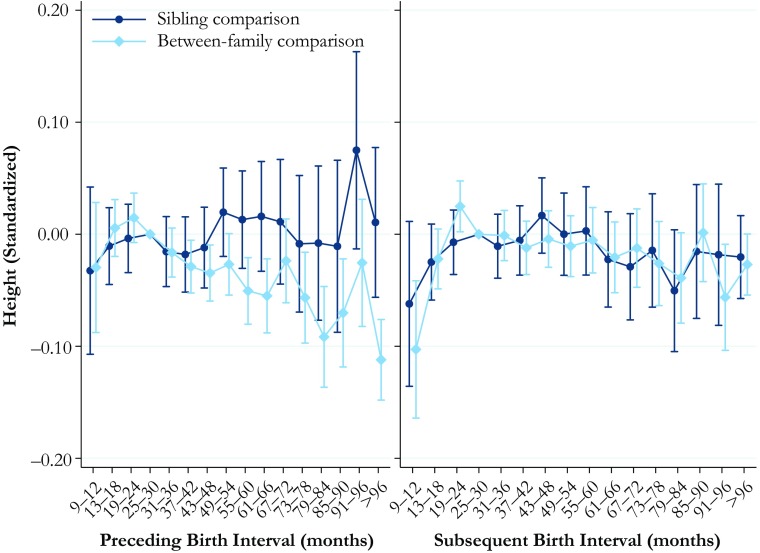
Height at ages 17–20 by preceding and subsequent birth intervals, Swedish men born in 1962–1979. The analysis population for examining preceding birth intervals consists of individuals in sibling groups with at least three children, excluding the firstborn. The analysis population for examining subsequent birth intervals consists of individuals in sibling groups with at least three male children, excluding the last-born. The reference category is a birth interval of 25–30 months. Error bars are 95 % confidence intervals

The results for the relationship between birth interval length and height standardized are shown in Fig. 2. One standard deviation for height is 6.5 cm. The full tables of the results are available in Online Resource 1, Tables S8 and S9. The results for the length of the preceding birth interval are shown in the left panel, and the results for the length of the subsequent interval in the right panel. Short preceding birth intervals are not significantly associated with height in the between-family comparison model, but longer birth intervals are associated with lower height. Individuals born after an interval of 61–66 are 5 % of a standard deviation shorter than those born after intervals of 25–30 months, while those born after intervals of 96 months or longer are 11 % of a standard deviation shorter than the reference category. However, the results from the sibling comparison model show no statistically significant differences in height by birth interval length.

The between-family analysis estimates for the association between the length of the subsequent birth interval and height (right panel, Fig. 2) show that individuals who experienced the birth of a sibling shortly after their own birth are estimated to be shorter. When the subsequent birth interval length was only 9–12 months, individuals were 10 % of a standard deviation shorter than if the birth interval had been 25–30 months. However, the within-family comparison analysis again suggests that this association may have been driven by unobserved factors associated with the timing and spacing of births as well as long-term health. The sibling comparison analysis reveals no statistically significant associations between the subsequent birth interval length and height.

### Physical Fitness

**Fig. 3:**
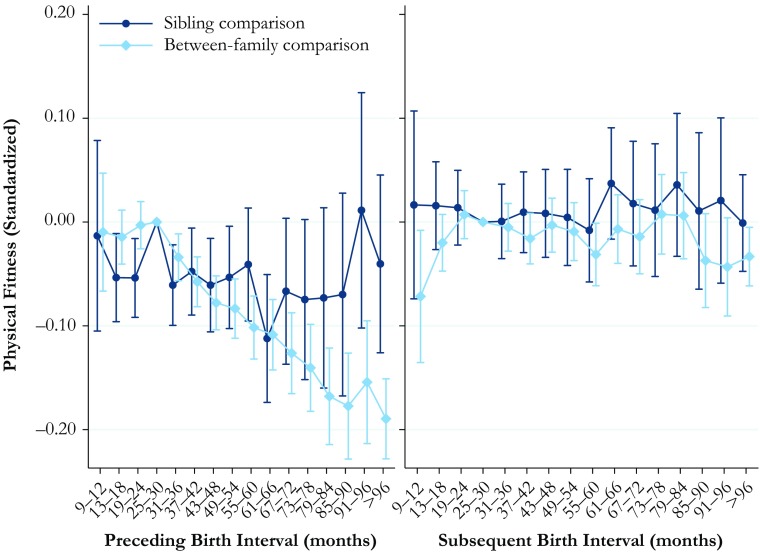
Physical fitness at ages 17–20 by preceding and subsequent birth intervals, Swedish men born in 1962–1979. The analysis population for examining preceding birth intervals consists of individuals in sibling groups with at least three children, excluding the firstborn. The analysis population for examining subsequent birth intervals consists of individuals in sibling groups with at least three male children, excluding the last-born. The reference category is a birth interval of 25–30 months. Error bars are 95 % confidence intervals

The results for the relationship between birth interval length and physical fitness are shown in Fig. 3. The full tables of the results are available in Online Resource 1, Tables S6 and S7. The between-family analyses in the left panel show that being born after a long birth interval is associated with substantially lower physical fitness. Indeed, those born after an interval of 96 months or longer have a maximal working capacity that is 20 % of a standard deviation lower than the reference category. In general, the results from the within-family comparison analysis do not show a particularly clear pattern in the association, but there is some indication that being born after a birth interval length of 31–54 months and being born after a birth interval of 13–24 months decrease physical fitness by approximately 5 % of a standard deviation in comparison with the reference category of 25–30 months.

The results for the relationship between the length of the subsequent interval and physical fitness are shown in the right panel of Fig. 3. The between-family analyses generally show no statistically significant relationship, but a very short subsequent interval of 9–12 months is associated with having a maximal working capacity that is 7 % of a standard deviation lower. The results from the within-family comparison, however, indicate that neither a very short subsequent interval nor any other subsequent interval length is associated with differences in physical fitness in early adulthood.

### Overweight or Obese

**Fig. 4:**
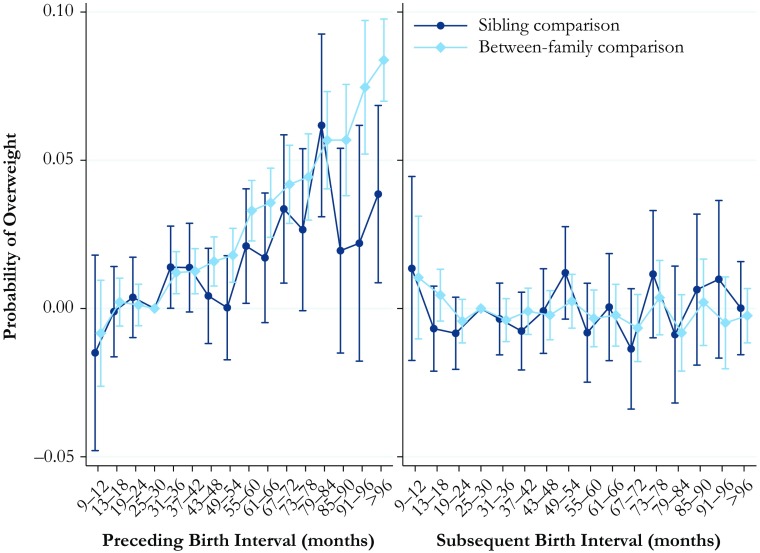
Predicted probability of being overweight or obese at ages 17–20 by preceding and subsequent birth intervals, Swedish men born in 1962–1979. The analysis population for examining preceding birth intervals consists of individuals in sibling groups with at least three children, excluding the firstborn. The analysis population for examining subsequent birth intervals consists of individuals in sibling groups with at least three male children, excluding the last-born. The reference category is a birth interval of 25–30 months. Error bars are 95 % confidence intervals

The results for the probability of being overweight or obese are shown in Fig. 4. The full tables are available in Online Resource 1, Tables S10 and S11. Because these analyses were conducted using linear probability models, the *y*-axis shows the predicted probability of high BMI in relation to birth interval length. The results for the length of the preceding interval, shown in the left panel of Fig. 4, indicate that being born after the interval of 31 months or longer is associated with a greater probability of being overweight or obese in early adulthood, and this probability increases the longer the birth interval was. In this case, we find that the results from the within-family sibling comparison model are relatively consistent with the results from the between-family comparison model. The results for the association between the subsequent interval length and the predicted probability of being overweight or obese are shown in the right panel of Fig. 4. Both the between-family and within-family comparison analyses indicate no statistically significant associations between subsequent interval length and the probability of being overweight or obese in early adulthood.

### Underweight or Severely Underweight

**Fig. 5:**
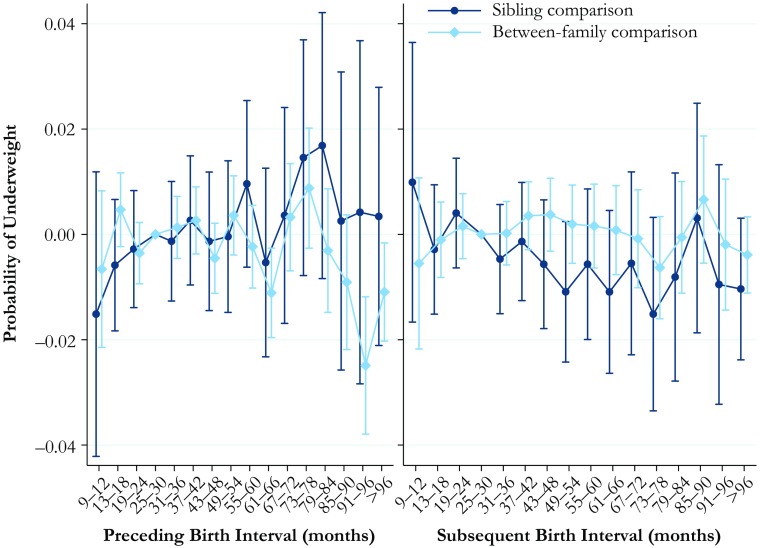
Predicted probability of being underweight at ages 17–20 by preceding and subsequent birth intervals, Swedish men born in 1962–1979. The analysis population for examining preceding birth intervals consists of individuals in sibling groups with at least three children, excluding the firstborn. The analysis population for examining subsequent birth intervals consists of individuals in sibling groups with at least three male children, excluding the last-born. The reference category is a birth interval of 25–30 months. Error bars are 95 % confidence intervals

The estimates for the relationship between firth interval length and the predicted probability of being underweight or severely underweight are shown in Fig. 5. The full tables of the results are available in Online Resource 1, Tables S12 and S13. Being underweight or severely underweight is less common than being overweight or obese in our data, which is unsurprising given that our data are based on males aged 17–20. We find few clear patterns in the association between birth interval lengths and being underweight or severely underweight for either the length of the preceding or the subsequent birth interval. The only statistically significant pattern that can be observed is that the between-family estimates indicate that those born after a long birth interval of 91 months or longer are less likely to be underweight. However, the association between birth interval length and the probability of being underweight is not visible in the within-family comparison model that adjusts for factors shared by siblings.

### Mortality

**Fig. 6:**
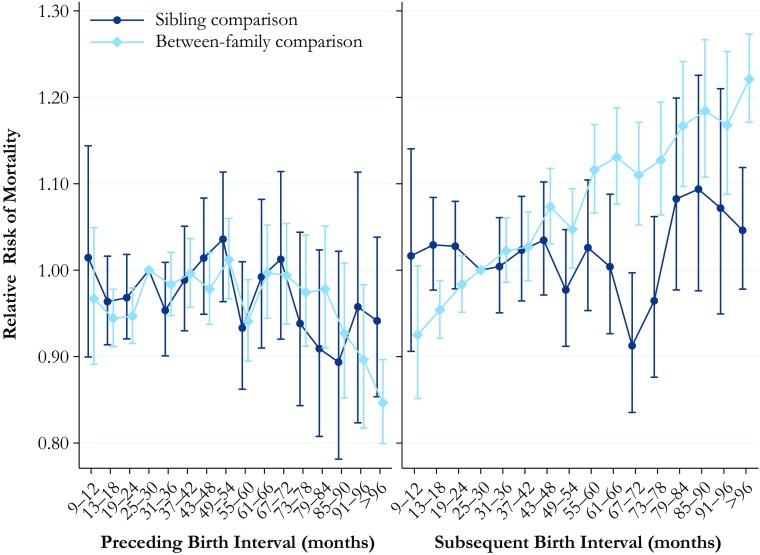
Hazard of mortality at ages 30–74 by preceding and subsequent birth intervals, Swedish men and women born in 1938–1960. The analysis population for examining preceding birth intervals consists of individuals in sibling groups with at least three children, excluding the firstborn. The analysis population for examining subsequent birth intervals consists of individuals in sibling groups with at least three children, excluding the last-born. The reference category is a birth interval of 25–30 months. Error bars are 95 % confidence intervals

Finally, the results for the association between birth interval length and the hazard of mortality in adulthood are shown in Fig. 6. The full tables of the results are available in Online Resource 1, Tables S14 and S15. The between-family analysis presented in the left panel, showing the risk of mortality in relation to the length of the preceding interval, indicates that those born after a short birth interval or after a particularly long birth interval of 91 months or longer have lower mortality than those born after an interval of 25–30 months. This finding is somewhat surprising given that previous research has typically indicated that short and long birth intervals are likely to have negative consequences. However, in the within-family comparison model, we find that the length of the preceding birth interval is not significantly associated with the hazard of mortality in adulthood. The results in the right hand panel—length of the subsequent interval—are far more pronounced. The results from the between-family analysis show that a longer subsequent birth interval is almost monotonically associated with a greater hazard of mortality in adulthood. Again, however, in the within-family comparison model (which adjusts for factors unobserved but shared by siblings), no statistically significant relationship exists between subsequent birth interval length and mortality in adulthood, although the point estimates may indicate a higher hazard for the longest birth intervals. We also conducted additional sex-stratified models, which are consistent with the pooled models presented earlier. The results from those models are shown in Online Resource 1, Figs. S1 and S2. We also investigated whether the interaction between gender and birth interval length is significant, but we found no statistically significant differences.

### Robustness Checks

We conducted a series of robustness checks using different operationalizations of the birth interval length to examine whether this produces different results. We reran our models using two binary specifications for the length of the preceding and subsequent birth interval: 18 months or longer, or 24 months or longer; in the within-family comparison models, we found no statistically significant effects of birth interval length on long-term outcomes. We also ran our analyses using linear and quadratic terms for birth interval length, again finding that when comparing siblings who share the same biological mother and father, birth interval length does not have a significant effect on long-term health outcomes. Those results are available in Online Resource 1, Tables S16 and S17. In further robustness checks, we examined whether the results for our analyses of being overweight or underweight are consistent when using logistic regression instead of a linear probability model. Those results are fully consistent with the main results presented earlier; see Online Resource 1, Figs. S3 and S4. We also examined whether the between-family estimates would be different had we estimated those models using population-averaged generalized estimating equations (GEE) instead of ordinary least squares (OLS) models with cluster-robust standard errors. As we show in Online Resource 1, Figs, S5–S8, the results are very similar regardless of whether we used the OLS or GEE estimation procedure.

Finally, we examined whether our results differ according to family size. We estimated our between-family OLS estimates separately on sibling groups with two, three, or four or more children; we estimated our sibling comparison models on sibling groups with three or four or more children for each health outcome that we study. Those results (shown in Online Resource 1, Figs. S9–S18) are generally very similar regardless of family size; the one exception is that the between-family estimates for mortality for the length of the subsequent interval in two-child sibling groups suggest a higher hazard of mortality among those with a sibling born 9–12 months after them. However, given that this model does not account for unobserved confounding, we hesitate to suggest that the relationship between the length of subsequent birth intervals and mortality is very different in two-child sibling groups.

## Discussion

In this study, we are one of the first to examine the relationship between birth interval length and long-term health outcomes. Although a previous study using historical data showed that a preceding birth interval shorter than two years was associated with substantially higher mortality at ages 55–74 (Campbell and Lee 2009), we are not aware of other research examining how birth interval length is related to long-term physical health in contemporary populations. When reducing residual confounding as much as possible by comparing siblings who share the same biological parents, we find that birth interval length was not significantly associated with height, physical fitness, or the probability of being underweight or severely underweight among men, or mortality among men and women. However, we do find some evidence to suggest that a long preceding birth interval was associated with a significantly higher probability of being overweight or obese among men aged 17–20. Given that a large body of previous research has shown that short and long birth intervals are associated with an increased risk of poor perinatal outcomes and poor long-term educational and socioeconomic outcomes, there was good reason to believe that this might also translate into worse long-term health. Overall, however, this study suggests that in a high-income country such as Sweden, even birth intervals as short as 9 to 12 months do not have much of a long-term effect on overall health.

The pattern that we observe for the probability of being overweight or obese in early adulthood is intriguing. When the preceding birth interval is 97 months or longer, the index person is, relative to the baseline, 34 % more likely to be overweight or obese—a substantial effect size. Previous research has suggested that preterm birth can affect adiposity (Uthaya et al. 2005), which can in turn affect the risk of becoming overweight or obese later in childhood and adulthood (Mathai et al. 2013). Furthermore, short and long birth intervals have been shown to be related to the risk of preterm birth (Conde-Agudelo et al. 2006). This is a possible explanation for the association that we observe between birth interval length and the probability of being overweight or obese in early adulthood; but if it is the explanation, it is surprising that we do not find that short birth intervals are also associated with an increased risk of becoming overweight or obese. The true explanation is unclear. One speculative explanation for our findings on being overweight is that birth spacing could be related to how the changing family environment is related to eating habits in childhood, such that a child born after a long birth interval is exposed to different food, and consumes more calories. For example, perhaps parents prepare the same dishes and a similar food portion size for all their children; for a sibling born after a long birth interval, this might mean that they are served a portion of food more suitable for a child several years older, or served food that would generally be introduced to only older children, thereby leading to higher calorie consumption through childhood. Indeed, a review of studies indicates that the eating patterns of children are strongly influenced by family and the social environment, and children model the behaviors of those around them (Patrick and Nicklas 2005). Furthermore, like adults (Wansink et al. 2005), children will generally eat what is put in front of them; larger portions lead to greater consumption (Fisher and Kral 2008). Ultimately, however, a definitive explanation for our finding remains elusive.

Although the sibling comparison approach that we implement in this study has the advantage of eliminating confounding from factors shared among siblings, perhaps we fail to adequately capture the influence of confounding factors that vary among siblings. We attempt to address this issue by adjusting for plausible potential confounding factors such as birth year, maternal age, and birth order, as well as age at conscription test attendance and the year of the conscription test. While residual confounding may remain, we consider this to be a relatively small problem given that we observe almost no statistically significant associations between birth interval length and health and mortality in adulthood. That is, our results are not being driven by omitted variable bias because we do not observe that birth intervals have much of an effect on long-term health. Another limitation of our study is that we do not adjust for the perinatal outcomes that we discuss as potential mediating factors for the relationship between birth interval length and the long-term health outcomes that we study because we did not have access to these variables. Nevertheless, because these perinatal outcomes are a consequence of the birth interval length and not a confounding factor, the failure to include these variables in our models does not bias our estimates for the effect of birth interval length on health in adulthood. Furthermore, again, apart from a higher probability of being overweight, we do not observe any negative effect of birth intervals on long-term health, even without adjusting for these theoretically important mediating factors.

Perhaps a more important limitation of our study is that the sibling comparison design that we implement is based on sibling groups with at least three children because there is no variance in birth interval length in a two-child sibling group. It is possible that the effect of birth intervals on long-term health is different in two-child sibling groups, the most common family size in Sweden (Andersson 1999), than it is in larger sibling groups. However, we think this is unlikely. The various theoretical hypotheses that have been proposed to explain a potential relationship between birth interval length and maternal and child health, as well as resource dilution in the household, should all be operating at a more severe level in larger sibling groups than in smaller sibling groups. Larger sibling groups would mean heavier dilution of resources. One would also expect that maternal nutrient depletion, folate depletion, cervical insufficiency, and the transmission of infections would be worse in larger sibling groups.

Another important issue is the external validity of our results. Although our estimates have excellent internal validity, the nature of the data available for the analysis may reduce the generalizability of our findings. The nature of our study design and data availability forces us to focus on sibling groups with three or more children and to exclude women from our analyses of the health outcomes measured by the military conscription tests. Although two-child families are the norm in Sweden (Andersson 1999), the majority of births intervals are observed in larger sibling groups. Given the distribution of family size in Sweden in the birth cohorts that we study, we estimate that 69 % of all birth intervals occurred in sibling groups with three or more children. Although it is not possible for us to know whether our findings regarding the negligible effect of birth intervals on height, fitness, and BMI are the same for women as they are for men, this would be an interesting topic for future work if suitable data is available elsewhere. However, given the null findings for men, and the null findings from the analyses of the relationship between birth intervals and mortality for both men and women, we anticipate no statistically significant relationship between birth intervals and length and height, fitness, or BMI for women.

A broader question concerns the degree to which our findings can be generalized to other countries. Sweden has a very high level of human development, very low infant mortality rates (WHO 2017), an excellent welfare system (Thakur et al. 2003), and widespread use of family planning techniques. Although we would expect similar results to be observed in countries with similar levels of development, such as Norway or Finland, it is more difficult to predict whether birth intervals might have a substantial effect on long-term health in countries with more severe levels of social stratification, such as the United States. It is certainly possible that short and long birth intervals could have a more serious negative effect on long-term outcomes among the most disadvantaged groups even in high-income countries. We would also suggest that given the strong relationship between birth interval length and the risk of poor perinatal outcomes and child mortality in low-income countries (Conde-Agudelo et al. 2006; Rutstein 2005; Rutstein and Winter 2014), birth intervals are likely to have meaningful long-term effects on health in contexts where public health conditions are much poorer than Sweden.

Research on the relationship between birth interval length and child outcomes in the short and long term has received a great deal of attention, but the received wisdom on the negative effects of short and very long birth intervals in high-income countries (e.g., Conde-Agudelo et al. 2006, 2007; Powell and Steelman 1990, 1993) has recently been challenged by a series of studies that question whether birth intervals actually do influence perinatal outcomes (Ball et al. 2014; Hanley et al. 2017) or educational and socioeconomic outcomes (Barclay and Kolk 2017). This study adds to this literature by showing that in a high-income country, birth interval length does not seem to influence long-term health, with the exception of a possible link to becoming overweight or obese. However, as the U.S. CDC noted (Copen et al. 2015), more research is needed on this topic before a new consensus can be reached. In particular, we encourage others with high-quality population or state-level data to emulate recent research, including this study, using a within-mother or sibling-comparison design (Ball et al. 2014; Barclay and Kolk 2017; Hanley et al. 2017; Koullali et al. 2017; Shachar et al. 2016) to evaluate the effect of birth spacing on short- and long-term child outcomes.

## Electronic supplementary material

ESM 1 (PDF 772 kb)
