# Supplementary material for: Birth Intervals and Health in Adulthood: A Comparison of Siblings Using Swedish Register Data
Source: Demography. 2018 May 21;55(3):929–55. doi: 10.1007/s13524-018-0673-8 (PMC5992250; doi:10.1007/s13524-018-0673-8)
Supplement: Supplementary file 1 — (PDF 772 kb) [file 13524_2018_673_MOESM1_ESM.pdf]

Online Resource 1

TABLE S1. Sample Exclusion Process

**Preceding Interval**	1962-1979		1938-1960	
	N	N excluded	N	N excluded
Total born in Sweden	1,951,202		2,491,059	
ID for both parents	1,927,677	23,525	2,210,012	281,047
All siblings born in Sweden	1,893,446	34,231	2,193,609	16,403
No multiple births	1,846,327	47,119	1,980,743	212,866
No half-siblings	1,347,819	498,508	1,735,140	245,603
Sibling group size >2	570,595	777,224	871,081	864,059
Males only	294,808	275,787	-	-
Outmigration or death before 1990	-	-	836,583	34,498
No first-borns	209,361	85,447	596,833	239,750
Cohort cut	125,340	84,021	581,544	15,289
Within-group variance in mortality	-	-	159,554	421,990
No missing values on model variables	~105,000	20,340	159,554	0
Final	~105,000		159,554	

**Subsequent Interval**	1962-1979		1938-1960	
	N	N excluded	N	N excluded
Total born in Sweden	1,951,202		2,491,059	
ID for both parents	1,927,677	23,525	2,210,012	281,047
All siblings born in Sweden	1,893,446	34,231	2,193,609	16,403
No multiple births	1,846,327	47,119	1,980,743	212,866
No half-siblings	1,347,819	498,508	1,735,140	245,603
Sibling group size >2	570,595	777,224	871,081	864,059
Males only	294,808	275,787	-	
Outmigration or death before 1990	-	-	836,583	34,498
No last-borns	198,431	96,377	639,628	196,955
Cohort cut	132,618	65,813	606,515	33,113
Within-group variance in mortality	-	-	150,907	455,608
No missing values on model variables	~110,000	22,618	150,907	0
Final	~110,000		150,907	

Note: Final sample for 1962-1979 cohorts varies slightly by outcome variable.

TABLE S2. Descriptive Statistics: Length of the Preceding Birth Interval in Relation to Physical Fitness, Height, and Being Underweight or Severely Underweight at Ages 17-20.

			Preceding Birth Interval																
			9-12	13-18	19-24	25-30	31-36	37-42	43-48	49-54	55-60	61-66	67-72	73-78	79-84	85-90	91-96	>96	Everyone
Physical		N	1,148	8,522	13,550	14,266	13,648	10,784	8,575	6,733	5,461	4,016	3,142	2,521	2,061	1,520	1,135	3,565	100,647
Fitness (watts)		Mean fitness	295.1	297.0	299.6	301.4	300.9	299.9	299.0	299.1	298.3	298.6	298.9	299.1	298.1	297.8	299.4	297.5	299.4
	Birth order	Mean	2.6	2.7	2.6	2.5	2.6	2.7	2.8	2.9	3.0	3.1	3.1	3.1	3.2	3.2	3.2	3.2	2.8
	Maternal age	Mean	24.9	26.3	26.7	27.0	27.5	28.1	28.8	29.6	30.3	30.8	31.3	31.7	32.3	32.8	33.3	34.8	28.5
	Birth year	Mean	1969.6	1969.9	1970.4	1970.8	1970.9	1970.9	1970.9	1971.0	1971.0	1971.2	1971.4	1971.7	1971.9	1972.3	1972.5	1973.1	1970.9
	Sibling group size	Mean	3.8	3.8	3.6	3.5	3.4	3.4	3.5	3.5	3.5	3.5	3.5	3.4	3.5	3.5	3.4	3.5	3.5
	Fitness by birth year	1962-1964	245.9	258.2	262.2	258.5	267.9	262.4	263.8	256.2	256.7	276.3	310.7	242.5	198.0	287.5	252.0	261.2	261.3
		1965-1969	290.5	291.1	293.1	294.6	295.0	293.4	293.3	292.3	291.8	293.1	294.5	293.1	298.3	295.4	295.0	288.3	293.3
		1970-1974	303.7	303.4	305.4	307.3	304.3	304.9	301.8	303.1	302.9	301.5	299.8	301.1	297.7	297.7	299.7	300.6	303.5
		1975-1979	294.3	304.8	306.6	306.3	307.0	304.4	305.4	304.6	301.9	301.5	303.7	302.7	298.8	300.2	302.5	297.0	304.4
Height (cm)		N	1,184	8,906	14,266	15,156	14,538	11,540	9,191	7,228	5,875	4,321	3,416	2,730	2,227	1,659	1,246	4,027	107,510
		Mean height	178.8	179.3	179.5	179.5	179.5	179.5	179.5	179.6	179.5	179.5	179.7	179.6	179.4	179.6	179.9	179.5	179.5
	Birth order	Mean	2.6	2.7	2.6	2.5	2.6	2.7	2.8	2.9	3.0	3.1	3.1	3.1	3.2	3.2	3.2	3.2	2.8
	Maternal age	Mean	24.9	26.3	26.7	27.0	27.5	28.1	28.8	29.6	30.3	30.8	31.3	31.8	32.3	32.8	33.3	34.8	28.5
	Birth year	Mean	1969.8	1970.2	1970.7	1971.1	1971.3	1971.3	1971.3	1971.4	1971.4	1971.6	1971.9	1972.1	1972.3	1972.7	1972.9	1973.5	1971.3
	Sibling group size	Mean	3.8	3.8	3.6	3.5	3.4	3.4	3.5	3.5	3.5	3.5	3.5	3.4	3.5	3.5	3.5	3.5	3.5
	Fitness by birth year	1962-1964	176.7	178.1	179.3	178.6	180.5	178.2	180.8	179.5	179.3	180.6	181.7	177.0	175.0	179.0	179.0	180.2	179.1
		1965-1969	178.4	179.0	179.2	179.2	179.3	179.1	179.3	179.4	179.1	179.2	179.7	179.3	178.9	179.4	179.3	179.0	179.2
		1970-1974	179.5	179.6	179.8	179.6	179.5	179.7	179.4	179.6	179.8	179.5	179.5	179.7	179.4	179.6	180.0	179.5	179.6
		1975-1979	178.8	179.6	179.7	179.9	179.7	179.8	179.6	179.8	179.4	179.8	180.1	179.8	179.8	179.6	180.2	179.6	179.7
Underweight		N	1,132	8,530	13,643	14,422	13,813	10,957	8,698	6,852	5,571	4,086	3,197	2,555	2,091	1,561	1,143	3,749	102,000
or Severely		%	6.4	7.5	6.5	6.8	6.9	7.1	6.4	7.3	6.7	5.9	7.3	7.9	6.7	6.1	4.5	6.0	6.8
Underweight	Birth order	Mean	2.7	2.6	2.6	2.5	2.6	2.7	2.8	2.9	3.0	3.1	3.1	3.1	3.2	3.2	3.2	3.2	2.7
	Maternal age	Mean	25.0	26.3	26.7	27.0	27.5	28.2	28.8	29.6	30.3	30.8	31.3	31.7	32.4	32.8	33.3	34.8	28.6
	Birth year	Mean	1969.7	1970.1	1970.6	1971.0	1971.2	1971.2	1971.2	1971.3	1971.3	1971.5	1971.8	1971.9	1972.2	1972.6	1972.8	1973.4	1971.2
	Sibling group size	Mean	3.8	3.8	3.6	3.5	3.4	3.4	3.5	3.5	3.5	3.5	3.5	3.4	3.5	3.5	3.5	3.5	3.5
	% by birth year	1962-1964	0.0	5.3	6.3	8.5	3.7	9.0	7.5	7.1	0.0	0.0	0.0	0.0	0.0	0.0	0.0	0.0	6.2
		1965-1969	6.4	7.9	6.9	7.4	7.6	8.0	6.7	8.0	7.1	6.6	8.1	10.1	8.3	4.3	4.3	5.3	7.4
		1970-1974	6.6	7.9	6.8	6.7	7.1	7.2	7.0	7.3	7.0	5.9	7.5	7.4	6.8	7.3	5.9	6.5	7.0
		1975-1979	6.1	5.9	5.3	5.9	5.8	5.6	5.1	6.2	5.7	4.9	6.2	6.3	5.0	5.6	2.9	5.7	5.6

TABLE S3. Descriptive Statistics: Length of the Preceding Birth Interval in Relation to Being Overweight or Obese at Ages 17-20, and Mortality at Ages 30-74.

			Preceding Birth Interval																
			9-12	13-18	19-24	25-30	31-36	37-42	43-48	49-54	55-60	61-66	67-72	73-78	79-84	85-90	91-96	>96	Everyone
Overweight		N	1,132	8,530	13,643	14,422	13,813	10,957	8,698	6,852	5,571	4,086	3,197	2,555	2,091	1,561	1,143	3,749	102,000
or Obese		%	9.5	10.2	9.9	9.8	11.0	11.0	11.3	11.5	13.0	13.2	13.9	14.1	15.3	15.4	17.1	18.0	11.5
	Birth order	Mean	2.7	2.6	2.6	2.5	2.6	2.7	2.8	2.9	3.0	3.1	3.1	3.1	3.2	3.2	3.2	3.2	2.7
	Maternal age	Mean	25.0	26.3	26.7	27.0	27.5	28.2	28.8	29.6	30.3	30.8	31.3	31.7	32.4	32.8	33.3	34.8	28.6
	Birth year	Mean	1969.7	1970.1	1970.6	1971.0	1971.2	1971.2	1971.2	1971.3	1971.3	1971.5	1971.8	1971.9	1972.2	1972.6	1972.8	1973.4	1971.2
	Sibling group size	Mean	3.8	3.8	3.6	3.5	3.4	3.4	3.5	3.5	3.5	3.5	3.5	3.4	3.5	3.5	3.5	3.5	3.5
	% by birth year	1962-1964	7.7	9.6	7.7	7.7	13.6	4.5	5.7	2.4	10.0	0.0	0.0	0.0	0.0	25.0	0.0	33.3	8.0
		1965-1969	8.1	9.7	8.8	8.5	9.9	9.8	10.0	9.8	12.5	11.5	12.0	10.8	13.3	15.4	16.9	16.6	10.0
		1970-1974	11.2	9.3	9.7	10.0	10.8	10.9	11.1	11.8	12.0	13.5	14.5	13.7	14.8	13.7	17.1	17.3	11.5
		1975-1979	10.2	13.1	12.7	11.7	12.6	13.1	13.8	13.5	15.0	14.9	15.1	17.9	18.0	17.9	17.2	19.1	13.9
Mortality	Mortality rate	(10^{-3})	1.22	1.20	1.18	1.25	1.23	1.24	1.21	1.22	1.12	1.16	1.16	1.09	1.06	1.00	0.95	0.84	1.18
	Deaths		659	6,322	7,366	6,230	5,038	3,982	3,269	2,586	2,041	1,659	1,349	1,026	822	596	477	1,635	45,057
	Person-time	%	1.4	13.8	16.2	13.0	10.7	8.4	7.0	5.5	4.8	3.7	3.0	2.5	2.0	1.6	1.3	5.1	100.0
	Sex	%	47.0	46.5	48.0	46.1	46.8	46.5	46.6	46.9	47.1	46.2	46.9	48.2	46.5	48.1	46.3	47.2	46.9
	Birth order	Mean	3.1	3.3	3.3	3.3	3.2	3.2	3.2	3.2	3.2	3.2	3.2	3.3	3.3	3.3	3.3	3.3	3.2
	Maternal age	Mean	26.8	28.0	29.2	29.9	30.3	30.8	31.3	31.7	32.3	32.9	33.1	33.7	34.2	34.6	35.1	36.8	30.8
	Birth year	Mean	1947.0	1946.6	1946.8	1947.2	1947.7	1948.0	1948.4	1948.7	1949.2	1949.4	1949.7	1950.2	1950.7	1951.2	1951.6	1953.5	1948.2
	Sibling group size	Mean	5.0	4.9	4.8	4.6	4.4	4.3	4.2	4.1	4.1	4.0	3.9	3.9	3.9	3.8	3.8	3.6	4.4
	Rate by birth year	1938-1940	1.50	1.73	1.93	2.16	2.31	2.46	2.48	2.58	2.63	2.85	2.66	2.70	3.04	2.98	2.59	2.76	2.12
		1941-1945	1.45	1.31	1.30	1.41	1.42	1.48	1.52	1.73	1.59	1.85	1.87	2.01	2.16	2.01	2.39	2.58	1.47
		1946-1950	1.09	1.04	1.00	1.06	1.06	1.09	1.06	1.03	0.96	1.08	1.09	1.04	1.07	1.17	1.06	1.29	1.05
		1951-1955	0.97	0.87	0.88	0.94	0.91	0.89	0.89	0.84	0.84	0.78	0.83	0.81	0.73	0.75	0.68	0.65	0.85
		1956-1960	0.97	1.00	0.88	0.91	0.88	0.89	0.86	0.83	0.70	0.56	0.65	0.53	0.62	0.49	0.61	0.54	0.77

TABLE S4. Descriptive Statistics: Length of the Subsequent Birth Interval in Relation to Physical Fitness, Height, and Being Underweight or Severely Underweight at Ages 17-20.

			Subsequent Birth Interval																
			9-12	13-18	19-24	25-30	31-36	37-42	43-48	49-54	55-60	61-66	67-72	73-78	79-84	85-90	91-96	>96	Everyone
Physical Fitness		N	1,017	7,658	12,646	13,724	13,474	10,691	8,793	6,950	5,771	4,607	3,600	3,013	2,585	2,119	1,718	7,576	105,942
		Mean fitness	294.4	298.5	301.7	302.2	302.3	301.5	302.4	301.4	299.8	301.5	300.8	302.3	301.5	299.5	299.4	299.7	301.2
	Birth order	Mean	1.6	1.6	1.5	1.5	1.5	1.6	1.7	1.8	1.9	2.0	2.1	2.1	2.1	2.1	2.2	2.2	1.7
	Maternal age	Mean	23.9	25.1	24.9	24.8	24.7	24.9	25.1	25.2	25.4	25.6	25.5	25.6	25.5	25.6	25.6	25.2	25.1
	Birth year	Mean	1969.4	1970.0	1970.6	1970.9	1971.0	1971.0	1971.1	1971.0	1971.0	1971.2	1971.0	1971.1	1971.2	1971.2	1971.5	1971.4	1970.9
	Sibling group size	Mean	3.8	3.7	3.6	3.5	3.4	3.4	3.4	3.4	3.4	3.4	3.4	3.4	3.4	3.4	3.4	3.4	3.5
	Fitness by birth year	1962-1964	250.6	261.1	260.5	269.3	274.5	272.8	258.6	262.1	265.1	258.1	256.2	267.4	239.1	240.5	270.0	256.6	264.0
		1965-1969	290.4	291.2	294.7	295.6	296.7	293.7	297.3	294.8	294.1	295.6	294.7	296.3	294.3	295.3	292.6	291.7	294.7
		1970-1974	302.2	307.0	307.5	308.1	306.6	307.8	306.7	306.4	304.6	306.3	305.6	307.0	305.2	301.5	303.6	304.3	306.5
		1975-1979	291.0	305.5	307.6	305.3	305.6	305.8	305.4	305.8	303.2	304.8	304.9	305.8	308.9	304.0	301.8	303.8	305.4
Height (cm)		N	1,043	8,025	13,464	14,639	14,422	11,457	9,423	7,450	6,182	4,966	3,869	3,246	2,787	2,260	1,861	8,156	113,250
		Mean height	178.6	179.4	179.8	179.6	179.6	179.5	179.6	179.5	179.5	179.4	179.4	179.4	179.3	179.5	179.1	179.3	179.5
	Birth order	Mean	1.6	1.6	1.5	1.5	1.5	1.6	1.7	1.8	1.9	2.0	2.0	2.1	2.1	2.1	2.2	2.2	1.7
	Maternal age	Mean	23.9	25.1	25.0	24.8	24.7	24.9	25.1	25.3	25.5	25.6	25.6	25.6	25.6	25.6	25.6	25.2	25.1
	Birth year	Mean	1969.6	1970.3	1971.0	1971.3	1971.4	1971.4	1971.5	1971.4	1971.4	1971.6	1971.5	1971.5	1971.6	1971.6	1972.0	1971.8	1971.3
	Sibling group size	Mean	3.8	3.7	3.6	3.5	3.4	3.4	3.4	3.4	3.4	3.4	3.4	3.4	3.4	3.4	3.4	3.4	3.5
	Fitness by birth year	1962-1964	177.9	178.3	179.7	179.3	180.1	179.2	179.1	179.0	181.0	180.1	179.3	181.5	179.1	178.0	182.5	179.1	179.4
		1965-1969	178.3	179.2	179.4	179.4	179.3	179.1	179.3	179.2	179.4	179.1	179.2	179.1	178.9	179.3	179.1	178.9	179.2
		1970-1974	178.9	179.8	179.8	179.8	179.8	179.7	179.7	179.6	179.4	179.5	179.7	179.6	179.5	179.9	179.2	179.5	179.7
		1975-1979	179.1	179.4	180.1	179.8	179.8	179.8	179.7	179.7	179.8	179.6	179.5	179.4	179.3	179.5	179.1	179.3	179.7
Underweight or Severely Underweight		N	997	7,688	12,885	14,012	13,782	10,973	8,965	7,082	5,907	4,727	3,653	3,070	2,648	2,142	1,772	7,721	108,024
		%	6.8	7.2	7.3	7.1	7.1	7.3	7.3	7.0	7.0	6.8	6.7	6.1	6.6	7.4	6.5	6.3	7.0
	Birth order	Mean	1.6	1.6	1.5	1.5	1.5	1.6	1.7	1.8	1.9	2.0	2.1	2.1	2.1	2.1	2.2	2.2	1.7
	Maternal age	Mean	24.0	25.1	25.0	24.8	24.8	24.9	25.1	25.3	25.5	25.6	25.6	25.6	25.6	25.6	25.7	25.2	25.1
	Birth year	Mean	1969.5	1970.2	1970.9	1971.2	1971.3	1971.3	1971.4	1971.3	1971.3	1971.5	1971.3	1971.4	1971.5	1971.5	1971.9	1971.7	1971.2
	Sibling group size	Mean	3.8	3.7	3.6	3.5	3.4	3.4	3.4	3.4	3.4	3.4	3.4	3.4	3.4	3.4	3.4	3.4	3.5
	% by birth year	1962-1964	12.5	11.7	10.6	2.7	5.5	7.7	10.0	0.0	8.9	8.0	12.5	12.5	11.1	20.0	0.0	7.0	7.7
		1965-1969	6.1	8.5	7.9	7.4	7.2	8.0	7.4	8.3	7.0	7.9	7.2	6.4	6.6	7.0	6.6	7.2	7.5
		1970-1974	7.9	6.7	7.4	7.2	7.5	7.5	7.2	7.5	8.4	6.4	7.1	6.6	7.5	9.4	5.7	6.5	7.3
		1975-1979	6.4	4.6	6.3	6.6	6.4	6.2	7.1	5.1	5.4	5.9	5.4	5.1	5.8	5.2	7.4	5.0	6.0

TABLE S5. Descriptive Statistics: Length of the Subsequent Birth Interval in Relation to Being Overweight or Obese at Ages 17-20, and Mortality at Ages 30-74.

			Subsequent Birth Interval																
			9-12	13-18	19-24	25-30	31-36	37-42	43-48	49-54	55-60	61-66	67-72	73-78	79-84	85-90	91-96	>96	Everyone
Overweight		N	997	7,688	12,885	14,012	13,782	10,973	8,965	7,082	5,907	4,727	3,653	3,070	2,648	2,142	1,772	7,721	108,024
or Obese		%	11.7	10.9	10.3	10.8	10.5	10.9	10.8	11.2	10.7	10.8	10.3	11.4	10.4	11.3	10.8	11.1	10.8
	Birth order	Mean	1.6	1.6	1.5	1.5	1.5	1.6	1.7	1.8	1.9	2.0	2.1	2.1	2.1	2.1	2.2	2.2	1.7
	Maternal age	Mean	24.0	25.1	25.0	24.8	24.8	24.9	25.1	25.3	25.5	25.6	25.6	25.6	25.6	25.6	25.7	25.2	25.1
	Birth year	Mean	1969.5	1970.2	1970.9	1971.2	1971.3	1971.3	1971.4	1971.3	1971.3	1971.5	1971.3	1971.4	1971.5	1971.5	1971.9	1971.7	1971.2
	Sibling group size	Mean	3.8	3.7	3.6	3.5	3.4	3.4	3.4	3.4	3.4	3.4	3.4	3.4	3.4	3.4	3.4	3.4	3.5
	% by birth year	1962-1964	0.0	5.8	6.2	10.9	7.3	7.7	8.0	14.3	13.3	8.0	0.0	18.8	0.0	0.0	20.0	11.6	8.4
		1965-1969	10.9	9.6	9.3	9.6	9.6	9.7	9.8	9.4	8.8	9.1	8.9	9.9	9.1	9.6	9.6	9.9	9.5
		1970-1974	12.9	11.1	9.8	10.9	10.0	10.3	9.9	11.7	9.9	10.8	10.0	10.0	9.6	11.3	10.3	10.4	10.4
		1975-1979	12.8	14.2	12.8	12.6	12.6	13.3	13.2	13.0	14.1	13.0	13.0	14.8	13.0	13.7	12.3	13.4	13.1
Mortality	Mortality rate	(10^{-3})	1.25	1.32	1.37	1.41	1.46	1.49	1.56	1.52	1.60	1.61	1.59	1.58	1.64	1.66	1.63	1.68	1.47
	Deaths		624	6,352	7,658	6,287	5,374	4,337	3,758	2,943	2,606	2,125	1,689	1,398	1,215	980	843	3,820	52,009
	Person-time	%	1.4	13.6	15.9	12.6	10.4	8.2	6.8	5.5	4.6	3.7	3.0	2.5	2.1	1.7	1.5	6.4	100.0
	Sex	%	49.5	47.3	46.5	46.7	47.2	46.3	45.9	45.3	43.4	45.6	44.4	46.5	45.4	45.2	46.4	45.8	46.3
	Birth order	Mean	2.0	2.2	2.3	2.3	2.2	2.2	2.2	2.3	2.3	2.4	2.4	2.5	2.5	2.5	2.5	2.5	2.285
	Maternal age	Mean	25.6	26.5	27.2	27.5	27.4	27.6	27.6	27.6	27.7	27.9	27.7	27.8	27.7	27.7	27.7	27.1	27.338
	Birth year	Mean	1946.5	1945.8	1945.7	1945.8	1946.0	1946.0	1946.1	1946.3	1946.4	1946.4	1946.6	1946.7	1946.9	1946.9	1947.0	1947.1	1946.121
	Sibling group size	Mean	4.9	4.8	4.7	4.5	4.4	4.2	4.1	4.1	4.0	4.0	3.9	3.9	3.9	3.8	3.8	3.7	4.346
	Rate by birth year	1938-1940	1.65	1.73	1.79	1.89	1.98	2.02	2.27	2.31	2.51	2.48	2.57	2.50	2.68	2.87	2.65	3.07	2.08
		1941-1945	1.31	1.39	1.44	1.51	1.55	1.60	1.61	1.60	1.73	1.78	1.79	1.73	1.84	1.87	1.95	1.94	1.57
		1946-1950	1.32	1.11	1.20	1.17	1.30	1.28	1.31	1.27	1.30	1.33	1.26	1.30	1.37	1.20	1.17	1.28	1.24
		1951-1955	0.87	1.10	1.07	1.12	1.12	1.16	1.19	1.11	1.22	1.13	1.09	1.20	1.16	1.22	1.23	1.31	1.14
		1956-1960	0.85	1.07	1.07	1.07	1.14	1.13	1.28	1.18	1.14	1.10	1.17	1.14	1.19	1.43	1.24	1.28	1.14

TABLE S6. Results: Relationship Between Preceding Birth Interval and Physical Fitness for Swedish Men Born 1962-1979. Estimates for Age at Conscription Test and Year of Conscription Test Not Shown.

		Within-family			Between-family		
		Beta	SE	95% CI	Beta	SE	95% CI
Preceding	9-12	-0.013	0.047	-0.105, 0.079	-0.010	0.029	-0.067, 0.047
Interval Length	13-18	-0.054	0.022	-0.096, -0.011	-0.015	0.013	-0.041, 0.011
	19-24	-0.054	0.019	-0.092, -0.016	-0.003	0.012	-0.026, 0.020
	25-30 (ref)	0.000			0.000		
	31-36	-0.061	0.020	-0.100, -0.022	-0.034	0.012	-0.057, -0.011
	37-42	-0.048	0.021	-0.090, -0.006	-0.058	0.012	-0.082, -0.034
	43-48	-0.061	0.023	-0.106, -0.016	-0.078	0.013	-0.104, -0.052
	49-54	-0.053	0.025	-0.103, -0.004	-0.083	0.015	-0.112, -0.055
	55-60	-0.041	0.028	-0.095, 0.013	-0.102	0.015	-0.132, -0.071
	61-66	-0.112	0.031	-0.174, -0.051	-0.109	0.017	-0.143, -0.075
	67-72	-0.067	0.036	-0.137, 0.004	-0.126	0.020	-0.165, -0.087
	73-78	-0.075	0.039	-0.152, 0.002	-0.140	0.021	-0.182, -0.099
	79-84	-0.073	0.044	-0.160, 0.014	-0.168	0.024	-0.214, -0.121
	85-90	-0.070	0.050	-0.168, 0.028	-0.177	0.026	-0.228, -0.126
	91-96	0.011	0.058	-0.102, 0.125	-0.154	0.030	-0.213, -0.095
	97+	-0.040	0.044	-0.126, 0.045	-0.190	0.020	-0.228, -0.151
Birth Order	2 (ref)	0.000			0.000		
	3	-0.066	0.020	-0.105, -0.026	-0.116	0.007	-0.131, -0.102
	4	-0.056	0.039	-0.133, 0.021	-0.189	0.014	-0.215, -0.162
	5	-0.136	0.060	-0.254, -0.018	-0.264	0.025	-0.312, -0.215
	6	-0.189	0.083	-0.352, -0.026	-0.310	0.037	-0.383, -0.236
	7+	-0.129	0.109	-0.342, 0.084	-0.291	0.048	-0.385, -0.198
Maternal Age	15-19	-0.099	0.065	-0.227, 0.028	-0.336	0.033	-0.402, -0.271
	20-24	0.006	0.021	-0.035, 0.048	-0.157	0.009	-0.174, -0.140
	25-29 (ref)	0.000			0.000		
	30-34	0.002	0.021	-0.038, 0.043	0.102	0.008	0.085, 0.118
	35-39	-0.029	0.040	-0.106, 0.049	0.122	0.013	0.097, 0.147
	40-44	-0.091	0.070	-0.228, 0.047	0.098	0.026	0.047, 0.150
	45+	0.042	0.194	-0.337, 0.421	0.057	0.109	-0.156, 0.270
Birth Year	1962	-0.924	0.764	-2.421, 0.574	-0.338	0.208	-0.747, 0.070
	1963	-1.197	0.250	-1.686, -0.707	-0.804	0.134	-1.067, -0.542
	1964	-0.771	0.166	-1.097, -0.445	-0.472	0.093	-0.655, -0.290
	1965	-0.576	0.144	-0.860, -0.293	-0.354	0.083	-0.517, -0.191
	1966	-0.330	0.119	-0.563, -0.096	-0.205	0.071	-0.344, -0.065
	1967	-0.253	0.100	-0.448, -0.057	-0.170	0.060	-0.288, -0.053
	1968	-0.310	0.076	-0.458, -0.161	-0.253	0.046	-0.343, -0.163
	1969	-0.091	0.054	-0.197, 0.014	-0.133	0.034	-0.200, -0.067
	1970 (ref)	0.000			0.000		
	1971	-0.072	0.049	-0.168, 0.023	-0.095	0.032	-0.158, -0.032
	1972	0.014	0.068	-0.119, 0.147	-0.036	0.044	-0.123, 0.051
	1973	-0.017	0.085	-0.183, 0.149	-0.026	0.054	-0.133, 0.080
	1974	0.006	0.101	-0.191, 0.204	-0.014	0.063	-0.138, 0.109
	1975	0.199	0.116	-0.028, 0.426	0.196	0.071	0.056, 0.336
	1976	0.344	0.133	0.084, 0.604	0.388	0.080	0.231, 0.545
	1977	0.527	0.156	0.222, 0.832	0.570	0.090	0.393, 0.746
	1978	0.683	0.181	0.328, 1.038	0.770	0.102	0.570, 0.970
	1979	0.841	0.225	0.400, 1.283	0.813	0.120	0.578, 1.048
Sibling	3 (ref)				0.000		
Group Size	4				-0.038	0.009	-0.056, -0.020
	5				-0.100	0.017	-0.133, -0.068
	6				-0.105	0.027	-0.157, -0.053
	7				-0.154	0.034	-0.221, -0.087
N			100,647			100,647	

TABLE S7. Results: Relationship Between Subsequent Birth Interval and Physical Fitness for Swedish Men Born 1962-1979. Estimates for Age at Conscription Test and Year of Conscription Test Not Shown.

		Within-family			Between-family		
		Beta	SE	95% CI	Beta	SE	95% CI
Subsequent Interval Length	9-12	0.016	0.046	-0.074, 0.107	-0.072	0.032	-0.135, -0.008
	13-18	0.016	0.022	-0.027, 0.058	-0.020	0.014	-0.047, 0.007
	19-24	0.014	0.018	-0.022, 0.050	0.007	0.012	-0.016, 0.030
	25-30 (ref)	0.000			0.000		
	31-36	0.001	0.018	-0.035, 0.037	-0.005	0.012	-0.028, 0.018
	37-42	0.009	0.020	-0.029, 0.048	-0.016	0.012	-0.040, 0.008
	43-48	0.008	0.022	-0.034, 0.051	-0.003	0.013	-0.029, 0.023
	49-54	0.004	0.024	-0.042, 0.051	-0.009	0.014	-0.037, 0.019
	55-60	-0.008	0.025	-0.058, 0.042	-0.031	0.015	-0.061, -0.001
	61-66	0.037	0.027	-0.017, 0.091	-0.007	0.017	-0.040, 0.026
	67-72	0.018	0.031	-0.042, 0.078	-0.014	0.018	-0.050, 0.022
	73-78	0.011	0.033	-0.052, 0.075	0.007	0.020	-0.031, 0.046
	79-84	0.036	0.035	-0.033, 0.105	0.006	0.021	-0.035, 0.048
	85-90	0.011	0.038	-0.065, 0.086	-0.037	0.023	-0.082, 0.008
	91-96	0.021	0.041	-0.059, 0.100	-0.043	0.024	-0.091, 0.004
	97+	-0.001	0.024	-0.048, 0.046	-0.033	0.014	-0.061, -0.005
Birth Order	1	0.000	0.021	-0.041, 0.041	0.069	0.007	0.055, 0.082
	2 (ref)	0.000			0.000		
	3	-0.024	0.024	-0.072, 0.024	-0.083	0.012	-0.107, -0.059
	4	0.021	0.048	-0.074, 0.116	-0.161	0.024	-0.208, -0.113
	5	-0.072	0.078	-0.225, 0.081	-0.214	0.043	-0.298, -0.130
	6	-0.074	0.113	-0.297, 0.148	-0.235	0.059	-0.351, -0.119
Maternal Age	15-19	-0.006	0.033	-0.070, 0.059	-0.256	0.013	-0.282, -0.230
	20-24	0.009	0.017	-0.024, 0.042	-0.139	0.007	-0.153, -0.125
	25-29 (ref)	0.000			0.000		
	30-34	-0.002	0.022	-0.046, 0.041	0.034	0.010	0.014, 0.055
	35-39	0.051	0.059	-0.065, 0.166	0.001	0.024	-0.045, 0.048
	40-44	0.291	0.197	-0.096, 0.678	-0.054	0.083	-0.217, 0.109
	45+				-0.075	0.032	-0.139, -0.012
Birth Year	1962	-0.272	0.824	-1.886, 1.343	-0.040	0.232	-0.494, 0.415
	1963	-1.312	0.260	-1.822, -0.801	-0.894	0.123	-1.136, -0.652
	1964	-0.677	0.157	-0.984, -0.370	-0.477	0.088	-0.650, -0.303
	1965	-0.727	0.133	-0.987, -0.466	-0.388	0.079	-0.543, -0.233
	1966	-0.432	0.108	-0.644, -0.221	-0.228	0.068	-0.362, -0.094
	1967	-0.337	0.086	-0.506, -0.169	-0.186	0.057	-0.298, -0.074
	1968	-0.436	0.066	-0.566, -0.306	-0.292	0.046	-0.381, -0.203
	1969	-0.190	0.047	-0.282, -0.098	-0.140	0.034	-0.206, -0.074
	1970 (ref)	0.000			0.000		
	1971	-0.056	0.044	-0.142, 0.030	-0.108	0.031	-0.169, -0.047
	1972	0.093	0.061	-0.027, 0.212	-0.011	0.043	-0.095, 0.073
	1973	0.068	0.075	-0.080, 0.215	-0.007	0.052	-0.108, 0.094
	1974	0.106	0.090	-0.070, 0.283	0.015	0.061	-0.103, 0.134
	1975	0.345	0.104	0.142, 0.548	0.189	0.069	0.055, 0.324
	1976	0.489	0.118	0.257, 0.721	0.418	0.076	0.269, 0.567
	1977	0.639	0.137	0.370, 0.908	0.623	0.085	0.456, 0.790
	1978	0.885	0.159	0.573, 1.198	0.862	0.096	0.673, 1.051
	1979	1.015	0.200	0.622, 1.407	0.914	0.113	0.693, 1.135
Sibling Group Size	3 (ref)				0.000		
	4				-0.056	0.009	-0.074, -0.038
	5				-0.132	0.017	-0.165, -0.100
	6				-0.133	0.028	-0.188, -0.079
	7				-0.171	0.035	-0.241, -0.102
N			105,942			105,942	

TABLE S8. Results: Relationship Between Preceding Birth Interval and Height for Swedish Men Born 1962-1979. Estimates for Age at Conscription Test and Year of Conscription Test Not Shown.

		Within-family			Between-family		
		Beta	SE	95% CI	Beta	SE	95% CI
Preceding	9-12	-0.033	0.038	-0.107, 0.042	-0.030	0.030	-0.088, 0.028
Interval Length	13-18	-0.011	0.017	-0.045, 0.024	0.006	0.013	-0.020, 0.031
	19-24	-0.004	0.016	-0.034, 0.027	0.015	0.011	-0.007, 0.037
	25-30 (ref)	0.000			0.000		
	31-36	-0.015	0.016	-0.047, 0.016	-0.016	0.011	-0.038, 0.006
	37-42	-0.018	0.017	-0.052, 0.016	-0.029	0.012	-0.052, -0.005
	43-48	-0.012	0.018	-0.048, 0.024	-0.035	0.013	-0.060, -0.010
	49-54	0.020	0.020	-0.020, 0.059	-0.027	0.014	-0.054, 0.001
	55-60	0.013	0.022	-0.030, 0.057	-0.051	0.015	-0.080, -0.021
	61-66	0.016	0.025	-0.033, 0.065	-0.055	0.017	-0.088, -0.022
	67-72	0.011	0.028	-0.044, 0.067	-0.024	0.019	-0.061, 0.014
	73-78	-0.009	0.031	-0.069, 0.052	-0.057	0.021	-0.097, -0.016
	79-84	-0.008	0.035	-0.077, 0.061	-0.092	0.023	-0.137, -0.047
	85-90	-0.011	0.039	-0.088, 0.066	-0.070	0.025	-0.118, -0.022
	91-96	0.075	0.045	-0.013, 0.163	-0.026	0.029	-0.082, 0.031
	97+	0.011	0.034	-0.056, 0.077	-0.112	0.018	-0.148, -0.076
Birth Order	2 (ref)	0.000			0.000		
	3	-0.048	0.016	-0.079, -0.017	-0.070	0.007	-0.083, -0.057
	4	-0.074	0.031	-0.135, -0.013	-0.140	0.013	-0.166, -0.115
	5	-0.108	0.048	-0.202, -0.014	-0.184	0.023	-0.229, -0.138
	6	-0.098	0.066	-0.228, 0.032	-0.225	0.036	-0.295, -0.155
	7	0.031	0.087	-0.139, 0.202	-0.286	0.052	-0.388, -0.185
Maternal Age	15-19	-0.025	0.053	-0.128, 0.079	-0.340	0.034	-0.408, -0.273
	20-24	0.013	0.017	-0.021, 0.046	-0.142	0.008	-0.158, -0.125
	25-29 (ref)	0.000			0.000		
	30-34	-0.009	0.017	-0.041, 0.024	0.110	0.008	0.095, 0.126
	35-39	-0.028	0.032	-0.090, 0.034	0.156	0.013	0.131, 0.181
	40-44	-0.064	0.056	-0.173, 0.046	0.154	0.025	0.105, 0.203
	45+	-0.064	0.152	-0.363, 0.234	0.182	0.092	0.002, 0.362
Birth Year	1962	0.213	0.640	-1.041, 1.467	0.654	0.352	-0.037, 1.344
	1963	-0.064	0.208	-0.471, 0.344	0.298	0.152	0.001, 0.596
	1964	-0.014	0.136	-0.281, 0.253	0.447	0.094	0.263, 0.631
	1965	0.072	0.118	-0.159, 0.304	0.423	0.082	0.262, 0.584
	1966	0.025	0.098	-0.167, 0.217	0.318	0.069	0.182, 0.453
	1967	0.016	0.082	-0.144, 0.176	0.275	0.059	0.160, 0.389
	1968	-0.016	0.062	-0.138, 0.106	0.225	0.045	0.136, 0.314
	1969	-0.020	0.044	-0.107, 0.067	0.108	0.034	0.041, 0.174
	1970 (ref)	0.000			0.000		
	1971	0.022	0.040	-0.056, 0.100	0.010	0.031	-0.052, 0.071
	1972	-0.021	0.055	-0.129, 0.087	-0.118	0.042	-0.202, -0.035
	1973	-0.074	0.068	-0.208, 0.060	-0.204	0.052	-0.306, -0.102
	1974	-0.096	0.081	-0.255, 0.063	-0.273	0.060	-0.391, -0.155
	1975	-0.116	0.093	-0.298, 0.066	-0.338	0.068	-0.471, -0.204
	1976	-0.081	0.106	-0.288, 0.127	-0.330	0.076	-0.479, -0.181
	1977	-0.047	0.122	-0.285, 0.192	-0.367	0.085	-0.533, -0.200
	1978	0.085	0.137	-0.183, 0.353	-0.275	0.093	-0.457, -0.093
	1979	0.082	0.158	-0.228, 0.392	-0.309	0.104	-0.512, -0.105
Sibling	3 (ref)				0.000		
Group Size	4				-0.026	0.009	-0.044, -0.008
	5				-0.056	0.017	-0.089, -0.024
	6				-0.082	0.026	-0.133, -0.031
	7				-0.084	0.036	-0.155, -0.013
N			107,510			107,510	

TABLE S9. Results: Relationship Between Subsequent Birth Interval and Height for Swedish Men Born 1962-1979. Estimates for Age at Conscription Test and Year of Conscription Test Not Shown.

		Within-family			Between-family		
		Beta	SE	95% CI	Beta	SE	95% CI
Subsequent	9-12	-0.062	0.038	-0.136, 0.011	-0.103	0.031	-0.164, -0.041
Interval Length	13-18	-0.025	0.017	-0.059, 0.009	-0.022	0.014	-0.049, 0.005
	19-24	-0.007	0.015	-0.036, 0.022	0.025	0.012	0.002, 0.048
	25-30 (ref)	0.000			0.000		
	31-36	-0.011	0.015	-0.039, 0.018	-0.001	0.011	-0.024, 0.021
	37-42	-0.006	0.016	-0.036, 0.025	-0.012	0.012	-0.036, 0.012
	43-48	0.017	0.017	-0.017, 0.050	-0.004	0.013	-0.030, 0.021
	49-54	0.000	0.019	-0.037, 0.037	-0.011	0.014	-0.038, 0.016
	55-60	0.003	0.020	-0.036, 0.042	-0.005	0.015	-0.035, 0.024
	61-66	-0.022	0.022	-0.065, 0.020	-0.021	0.016	-0.052, 0.011
	67-72	-0.029	0.024	-0.076, 0.018	-0.012	0.018	-0.047, 0.023
	73-78	-0.014	0.026	-0.065, 0.036	-0.026	0.019	-0.064, 0.011
	79-84	-0.050	0.028	-0.105, 0.004	-0.039	0.021	-0.079, 0.001
	85-90	-0.015	0.030	-0.075, 0.044	0.001	0.022	-0.042, 0.045
	91-96	-0.018	0.032	-0.081, 0.045	-0.056	0.024	-0.104, -0.009
	97+	-0.020	0.019	-0.057, 0.017	-0.027	0.014	-0.054, 0.000
Birth Order	1	0.033	0.016	0.001, 0.065	0.055	0.007	0.042, 0.068
	2 (ref)	0.000			0.000		
	3	-0.024	0.019	-0.061, 0.014	-0.065	0.011	-0.087, -0.042
	4	-0.014	0.038	-0.089, 0.061	-0.151	0.023	-0.197, -0.105
	5	-0.088	0.061	-0.207, 0.032	-0.165	0.041	-0.245, -0.084
	6	-0.119	0.089	-0.294, 0.056	-0.273	0.061	-0.393, -0.154
Maternal Age	15-19	0.021	0.026	-0.030, 0.072	-0.240	0.013	-0.266, -0.213
	20-24	-0.006	0.014	-0.033, 0.020	-0.124	0.007	-0.137, -0.110
	25-29 (ref)	0.000			0.000		
	30-34	0.025	0.017	-0.009, 0.059	0.079	0.010	0.059, 0.099
	35-39	0.015	0.046	-0.074, 0.105	0.068	0.024	0.022, 0.115
	40-44	0.028	0.153	-0.272, 0.329	0.012	0.083	-0.150, 0.175
	45+				0.200	0.034	0.134, 0.267
Birth Year	1962	0.219	0.687	-1.127, 1.565	0.647	0.381	-0.099, 1.393
	1963	0.112	0.215	-0.310, 0.534	0.090	0.139	-0.182, 0.362
	1964	0.211	0.129	-0.041, 0.463	0.450	0.089	0.275, 0.625
	1965	0.180	0.109	-0.034, 0.394	0.386	0.078	0.233, 0.540
	1966	0.111	0.089	-0.063, 0.284	0.318	0.066	0.189, 0.448
	1967	0.140	0.071	0.002, 0.279	0.257	0.056	0.147, 0.367
	1968	0.052	0.055	-0.055, 0.160	0.171	0.045	0.084, 0.258
	1969	0.003	0.039	-0.073, 0.079	0.085	0.033	0.020, 0.151
	1970 (ref)	0.000			0.000		
	1971	0.035	0.036	-0.036, 0.106	-0.047	0.031	-0.107, 0.014
	1972	-0.012	0.050	-0.110, 0.086	-0.132	0.042	-0.213, -0.050
	1973	-0.042	0.061	-0.162, 0.078	-0.196	0.050	-0.295, -0.097
	1974	-0.022	0.073	-0.164, 0.121	-0.246	0.059	-0.362, -0.131
	1975	-0.015	0.083	-0.178, 0.147	-0.325	0.067	-0.457, -0.194
	1976	0.035	0.094	-0.149, 0.219	-0.286	0.074	-0.431, -0.141
	1977	0.033	0.106	-0.176, 0.242	-0.320	0.082	-0.480, -0.160
	1978	0.081	0.119	-0.153, 0.314	-0.226	0.089	-0.400, -0.051
	1979	0.034	0.139	-0.238, 0.307	-0.245	0.100	-0.441, -0.050
Sibling	3 (ref)				0.000		
Group Size	4				-0.025	0.009	-0.043, -0.007
	5				-0.043	0.017	-0.077, -0.009
	6				-0.094	0.028	-0.148, -0.040
	7				-0.061	0.037	-0.134, 0.013
N			113,250			113,250	

TABLE S10. Results: Relationship Between Preceding Birth Interval and Being Overweight or Obese for Swedish Men Born 1962-1979. Estimates for Age at Conscription Test and Year of Conscription Test Not Shown.

		Within-family			Between-family		
		Beta	SE	95% CI	Beta	SE	95% CI
Preceding	9-12	-0.015	0.017	-0.048, 0.018	-0.008	0.009	-0.026, 0.010
Interval Length	13-18	-0.001	0.008	-0.016, 0.014	0.002	0.004	-0.006, 0.010
	19-24	0.004	0.007	-0.010, 0.017	0.001	0.004	-0.006, 0.008
	25-30 (ref)	0.000			0.000		
	31-36	0.014	0.007	0.000, 0.028	0.012	0.004	0.005, 0.019
	37-42	0.014	0.008	-0.001, 0.029	0.013	0.004	0.005, 0.020
	43-48	0.004	0.008	-0.012, 0.020	0.016	0.004	0.008, 0.024
	49-54	0.000	0.009	-0.017, 0.018	0.018	0.005	0.009, 0.027
	55-60	0.021	0.010	0.002, 0.040	0.033	0.005	0.023, 0.043
	61-66	0.017	0.011	-0.005, 0.039	0.036	0.006	0.024, 0.047
	67-72	0.034	0.013	0.009, 0.059	0.042	0.007	0.029, 0.055
	73-78	0.027	0.014	-0.001, 0.054	0.044	0.007	0.030, 0.059
	79-84	0.062	0.016	0.031, 0.093	0.057	0.008	0.040, 0.073
	85-90	0.020	0.018	-0.015, 0.054	0.057	0.010	0.038, 0.076
	91-96	0.022	0.020	-0.018, 0.062	0.075	0.011	0.052, 0.097
	97+	0.039	0.015	0.009, 0.068	0.084	0.007	0.070, 0.098
Birth Order	2 (ref)	0.000			0.000		
	3	0.007	0.007	-0.007, 0.021	0.023	0.002	0.019, 0.028
	4	0.021	0.014	-0.007, 0.048	0.050	0.005	0.041, 0.059
	5	0.017	0.021	-0.025, 0.059	0.073	0.008	0.056, 0.089
	6	0.041	0.030	-0.017, 0.099	0.090	0.013	0.064, 0.115
	7	0.001	0.039	-0.075, 0.077	0.122	0.017	0.090, 0.154
Maternal Age	15-19	-0.011	0.024	-0.058, 0.036	0.028	0.011	0.006, 0.050
	20-24	-0.006	0.008	-0.021, 0.009	0.032	0.003	0.027, 0.038
	25-29 (ref)	0.000			0.000		
	30-34	-0.003	0.007	-0.017, 0.012	-0.020	0.003	-0.025, -0.015
	35-39	-0.004	0.014	-0.032, 0.024	-0.026	0.004	-0.034, -0.018
	40-44	-0.034	0.025	-0.082, 0.015	-0.042	0.008	-0.059, -0.025
	45+	0.101	0.070	-0.035, 0.238	-0.063	0.032	-0.126, 0.000
Birth Year	1962	0.015	0.272	-0.519, 0.549	-0.127	0.138	-0.398, 0.145
	1963	0.052	0.094	-0.133, 0.236	-0.120	0.048	-0.215, -0.025
	1964	-0.015	0.060	-0.132, 0.103	-0.122	0.028	-0.178, -0.067
	1965	-0.020	0.052	-0.122, 0.082	-0.093	0.026	-0.143, -0.043
	1966	0.002	0.043	-0.083, 0.086	-0.064	0.022	-0.108, -0.021
	1967	-0.023	0.036	-0.094, 0.047	-0.056	0.019	-0.093, -0.020
	1968	-0.019	0.027	-0.073, 0.035	-0.043	0.014	-0.071, -0.015
	1969	-0.020	0.019	-0.058, 0.018	-0.031	0.010	-0.051, -0.011
	1970 (ref)	0.000			0.000		
	1971	0.017	0.018	-0.018, 0.051	0.031	0.010	0.012, 0.049
	1972	0.036	0.024	-0.012, 0.084	0.054	0.013	0.027, 0.080
	1973	0.020	0.030	-0.039, 0.080	0.059	0.017	0.027, 0.092
	1974	0.042	0.036	-0.029, 0.112	0.080	0.020	0.042, 0.118
	1975	0.050	0.041	-0.031, 0.130	0.098	0.022	0.054, 0.141
	1976	0.054	0.047	-0.038, 0.146	0.098	0.025	0.049, 0.147
	1977	0.110	0.054	0.004, 0.217	0.107	0.028	0.052, 0.163
	1978	0.127	0.061	0.007, 0.248	0.106	0.031	0.044, 0.167
	1979	0.084	0.072	-0.057, 0.226	0.110	0.036	0.040, 0.181
Sibling	3 (ref)				0.000		
Group Size	4				-0.005	0.003	-0.010, 0.001
	5				-0.009	0.005	-0.019, 0.001
	6				-0.018	0.008	-0.035, -0.002
	7				-0.039	0.010	-0.060, -0.019
N			102,000			102,000	

TABLE S11. Results: Relationship Between Subsequent Birth Interval and Being Overweight or Obese for Swedish Men Born 1962-1979. Estimates for Age at Conscription Test and Year of Conscription Test Not Shown.

		Within-family			Between-family		
		Beta	SE	95% CI	Beta	SE	95% CI
Subsequent Interval Length	9-12	0.014	0.016	-0.018, 0.045	0.010	0.011	-0.010, 0.031
	13-18	-0.007	0.007	-0.021, 0.008	0.005	0.004	-0.004, 0.013
	19-24	-0.008	0.006	-0.021, 0.004	-0.004	0.004	-0.012, 0.003
	25-30 (ref)	0.000			0.000		
	31-36	-0.004	0.006	-0.016, 0.009	-0.004	0.004	-0.011, 0.003
	37-42	-0.008	0.007	-0.021, 0.006	-0.001	0.004	-0.009, 0.007
	43-48	-0.001	0.007	-0.015, 0.013	-0.002	0.004	-0.011, 0.006
	49-54	0.012	0.008	-0.004, 0.028	0.002	0.005	-0.007, 0.012
	55-60	-0.008	0.009	-0.025, 0.009	-0.003	0.005	-0.013, 0.006
	61-66	0.000	0.009	-0.018, 0.019	-0.002	0.005	-0.013, 0.008
	67-72	-0.014	0.010	-0.034, 0.007	-0.007	0.006	-0.018, 0.005
	73-78	0.012	0.011	-0.010, 0.033	0.004	0.006	-0.009, 0.016
	79-84	-0.009	0.012	-0.032, 0.014	-0.008	0.007	-0.021, 0.005
	85-90	0.006	0.013	-0.019, 0.032	0.002	0.007	-0.012, 0.017
	91-96	0.010	0.014	-0.017, 0.036	-0.005	0.008	-0.020, 0.011
	97+	0.000	0.008	-0.016, 0.016	-0.002	0.005	-0.012, 0.007
Birth Order	1	0.023	0.007	0.009, 0.037	-0.006	0.002	-0.010, -0.001
	2 (ref)	0.000			0.000		
	3	-0.004	0.008	-0.020, 0.012	0.025	0.004	0.017, 0.033
	4	-0.003	0.016	-0.035, 0.029	0.040	0.008	0.024, 0.055
	5	-0.018	0.026	-0.069, 0.033	0.051	0.014	0.023, 0.078
	6	0.005	0.038	-0.069, 0.080	0.072	0.020	0.032, 0.111
Maternal Age	15-19	0.012	0.011	-0.010, 0.034	0.065	0.005	0.056, 0.074
	20-24	0.007	0.006	-0.004, 0.018	0.030	0.002	0.026, 0.034
	25-29	0.000			0.000		
	30-34	-0.007	0.007	-0.021, 0.008	-0.007	0.003	-0.013, -0.001
	35-39	-0.029	0.020	-0.067, 0.010	0.004	0.008	-0.011, 0.019
	40-44	-0.019	0.065	-0.145, 0.108	0.017	0.030	-0.041, 0.075
	45+				-0.085	0.006	-0.097, -0.074
Birth Year	1962	-0.071	0.279	-0.619, 0.476	-0.024	0.157	-0.332, 0.284
	1963	-0.093	0.091	-0.270, 0.085	-0.035	0.045	-0.123, 0.053
	1964	-0.023	0.054	-0.128, 0.083	-0.053	0.027	-0.106, 0.001
	1965	-0.002	0.046	-0.091, 0.087	-0.026	0.025	-0.075, 0.023
	1966	0.017	0.037	-0.056, 0.089	-0.021	0.021	-0.063, 0.021
	1967	0.017	0.030	-0.041, 0.075	-0.009	0.018	-0.045, 0.027
	1968	0.016	0.023	-0.029, 0.061	-0.008	0.014	-0.036, 0.020
	1969	0.030	0.016	-0.002, 0.062	0.010	0.011	-0.012, 0.031
	1970 (ref)	0.000			0.000		
	1971	0.002	0.015	-0.027, 0.032	0.016	0.009	-0.002, 0.035
	1972	0.010	0.021	-0.031, 0.052	0.025	0.013	-0.001, 0.051
	1973	0.021	0.026	-0.030, 0.072	0.044	0.016	0.013, 0.075
	1974	0.035	0.031	-0.025, 0.095	0.065	0.019	0.028, 0.101
	1975	0.053	0.035	-0.015, 0.122	0.081	0.021	0.040, 0.122
	1976	0.055	0.040	-0.022, 0.133	0.067	0.024	0.020, 0.113
	1977	0.059	0.045	-0.030, 0.148	0.071	0.027	0.018, 0.123
	1978	0.060	0.051	-0.040, 0.160	0.069	0.029	0.012, 0.127
	1979	0.064	0.060	-0.054, 0.181	0.053	0.034	-0.013, 0.119
Sibling Group Size	3 (ref)				0.000		
	4				-0.004	0.003	-0.010, 0.001
	5				-0.004	0.005	-0.014, 0.006
	6				-0.004	0.009	-0.021, 0.014
	7				-0.033	0.010	-0.053, -0.013
N			108,024			108,024	

TABLE S12. Results: Relationship Between Preceding Birth Interval and Being Underweight or Severely Underweight for Swedish Men Born 1962-1979. Estimates for Age at Conscription Test and Year of Conscription Test Not Shown.

		Within-family			Between-family		
		Beta	SE	95% CI	Beta	SE	95% CI
Preceding Interval Length	9-12	-0.015	0.014	-0.042, 0.012	-0.007	0.008	-0.021, 0.008
	13-18	-0.006	0.006	-0.018, 0.007	0.005	0.004	-0.002, 0.012
	19-24	-0.003	0.006	-0.014, 0.008	-0.004	0.003	-0.009, 0.002
	25-30 (ref)	0.000			0.000		
	31-36	-0.001	0.006	-0.013, 0.010	0.001	0.003	-0.005, 0.007
	37-42	0.003	0.006	-0.010, 0.015	0.003	0.003	-0.004, 0.009
	43-48	-0.001	0.007	-0.014, 0.012	-0.005	0.003	-0.011, 0.002
	49-54	0.000	0.007	-0.015, 0.014	0.004	0.004	-0.004, 0.011
	55-60	0.010	0.008	-0.006, 0.025	-0.002	0.004	-0.010, 0.006
	61-66	-0.005	0.009	-0.023, 0.013	-0.011	0.004	-0.020, -0.003
	67-72	0.004	0.010	-0.017, 0.024	0.003	0.005	-0.007, 0.013
	73-78	0.015	0.011	-0.008, 0.037	0.009	0.006	-0.003, 0.020
	79-84	0.017	0.013	-0.008, 0.042	-0.003	0.006	-0.015, 0.009
	85-90	0.003	0.014	-0.026, 0.031	-0.009	0.007	-0.022, 0.004
	91-96	0.004	0.017	-0.028, 0.037	-0.025	0.007	-0.038, -0.012
	97+	0.003	0.013	-0.021, 0.028	-0.011	0.005	-0.020, -0.002
Birth Order	2 (ref)	0.000			0.000		
	3	0.010	0.006	-0.002, 0.021	-0.002	0.002	-0.006, 0.002
	4	0.006	0.011	-0.016, 0.028	-0.008	0.004	-0.015, -0.001
	5	-0.004	0.018	-0.038, 0.031	-0.025	0.007	-0.038, -0.012
	6	0.003	0.024	-0.044, 0.051	-0.032	0.011	-0.054, -0.011
	7	0.000	0.032	-0.063, 0.062	-0.054	0.014	-0.081, -0.026
Maternal Age	15-19	0.000	0.019	-0.038, 0.038	-0.013	0.009	-0.030, 0.005
	20-24	-0.005	0.006	-0.017, 0.007	-0.005	0.002	-0.009, 0.000
	25-29 (ref)	0.000			0.000		
	30-34	0.006	0.006	-0.005, 0.018	0.003	0.002	-0.001, 0.008
	35-39	0.021	0.012	-0.002, 0.044	0.013	0.003	0.006, 0.019
	40-44	0.070	0.020	0.031, 0.110	0.026	0.007	0.012, 0.040
	45+	0.083	0.057	-0.029, 0.195	0.006	0.024	-0.042, 0.054
Birth Year	1962	0.000	0.223	-0.437, 0.438	-0.080	0.028	-0.135, -0.026
	1963	-0.052	0.077	-0.203, 0.099	0.009	0.039	-0.067, 0.084
	1964	-0.057	0.049	-0.153, 0.040	-0.016	0.024	-0.063, 0.030
	1965	-0.021	0.043	-0.105, 0.062	-0.012	0.021	-0.053, 0.029
	1966	-0.026	0.035	-0.095, 0.043	-0.010	0.018	-0.045, 0.025
	1967	-0.034	0.029	-0.092, 0.024	-0.016	0.015	-0.045, 0.014
	1968	-0.038	0.022	-0.082, 0.006	-0.008	0.012	-0.031, 0.015
	1969	-0.008	0.016	-0.039, 0.023	0.001	0.009	-0.016, 0.019
	1970 (ref)	0.000			0.000		
	1971	0.009	0.014	-0.019, 0.037	0.009	0.008	-0.007, 0.024
	1972	0.003	0.020	-0.036, 0.043	0.020	0.011	0.000, 0.041
	1973	0.010	0.025	-0.039, 0.058	0.022	0.013	-0.003, 0.047
	1974	0.011	0.029	-0.047, 0.068	0.012	0.015	-0.017, 0.042
	1975	0.019	0.034	-0.047, 0.085	0.009	0.017	-0.024, 0.042
	1976	0.018	0.039	-0.058, 0.093	0.002	0.019	-0.034, 0.039
	1977	0.001	0.044	-0.086, 0.088	0.001	0.021	-0.040, 0.042
	1978	0.013	0.050	-0.086, 0.112	0.016	0.023	-0.028, 0.060
	1979	0.031	0.059	-0.085, 0.147	0.031	0.025	-0.019, 0.080
Sibling Group Size	3 (ref)				0.000		
	4				0.001	0.002	-0.003, 0.006
	5				0.020	0.004	0.012, 0.029
	6				0.022	0.007	0.008, 0.036
	7				0.048	0.010	0.027, 0.068
N			102,000			102,000	

TABLE S13. Results: Relationship Between Subsequent Birth Interval and Being Underweight or Severely Underweight for Swedish Men Born 1962-1979. Estimates for Age at Conscription Test and Year of Conscription Test Not Shown.

		Within-family			Between-family		
		Beta	SE	95% CI	Beta	SE	95% CI
Subsequent Interval Length	9-12	0.010	0.014	-0.017, 0.036	-0.006	0.008	-0.022, 0.011
	13-18	-0.003	0.006	-0.015, 0.009	-0.001	0.004	-0.008, 0.006
	19-24	0.004	0.005	-0.006, 0.014	0.002	0.003	-0.005, 0.008
	25-30 (ref)	0.000			0.000		
	31-36	-0.005	0.005	-0.015, 0.006	0.000	0.003	-0.006, 0.006
	37-42	-0.001	0.006	-0.013, 0.010	0.004	0.003	-0.003, 0.010
	43-48	-0.006	0.006	-0.018, 0.007	0.004	0.004	-0.003, 0.011
	49-54	-0.011	0.007	-0.024, 0.002	0.002	0.004	-0.005, 0.009
	55-60	-0.006	0.007	-0.020, 0.009	0.002	0.004	-0.006, 0.010
	61-66	-0.011	0.008	-0.026, 0.005	0.001	0.004	-0.008, 0.009
	67-72	-0.005	0.009	-0.023, 0.012	-0.001	0.005	-0.010, 0.008
	73-78	-0.015	0.009	-0.034, 0.003	-0.006	0.005	-0.016, 0.003
	79-84	-0.008	0.010	-0.028, 0.012	-0.001	0.005	-0.011, 0.010
	85-90	0.003	0.011	-0.019, 0.025	0.007	0.006	-0.005, 0.019
	91-96	-0.010	0.012	-0.032, 0.013	-0.002	0.006	-0.014, 0.010
	97+	-0.010	0.007	-0.024, 0.003	-0.004	0.004	-0.011, 0.003
Birth Order	1	-0.013	0.006	-0.025, -0.002	0.007	0.002	0.003, 0.011
	2 (ref)	0.000			0.000		
	3	0.023	0.007	0.009, 0.036	0.004	0.003	-0.003, 0.011
	4	0.031	0.014	0.004, 0.058	0.007	0.007	-0.006, 0.021
	5	0.031	0.022	-0.012, 0.075	-0.008	0.011	-0.031, 0.014
	6	0.056	0.032	-0.007, 0.120	-0.017	0.018	-0.052, 0.019
Maternal Age	15-19	0.005	0.010	-0.014, 0.024	-0.008	0.003	-0.015, -0.002
	20-24	0.003	0.005	-0.007, 0.012	-0.002	0.002	-0.005, 0.002
	25-29 (ref)	0.000			0.000		
	30-34	-0.004	0.006	-0.016, 0.009	0.005	0.003	-0.001, 0.010
	35-39	-0.016	0.017	-0.049, 0.016	0.004	0.007	-0.009, 0.017
	40-44	0.082	0.055	-0.026, 0.190	0.036	0.028	-0.019, 0.090
	45+				-0.072	0.003	-0.078, -0.065
Birth Year	1962	0.066	0.239	-0.402, 0.535	-0.052	0.026	-0.104, -0.001
	1963	-0.046	0.078	-0.198, 0.106	0.023	0.034	-0.044, 0.090
	1964	0.013	0.046	-0.077, 0.103	0.011	0.023	-0.034, 0.056
	1965	0.012	0.039	-0.064, 0.089	-0.002	0.020	-0.041, 0.037
	1966	0.026	0.032	-0.036, 0.088	0.007	0.018	-0.027, 0.042
	1967	0.019	0.025	-0.031, 0.068	0.000	0.015	-0.029, 0.029
	1968	0.017	0.020	-0.021, 0.055	0.003	0.012	-0.020, 0.026
	1969	0.024	0.014	-0.003, 0.051	0.013	0.009	-0.005, 0.031
	1970 (ref)	0.000			0.000		
	1971	0.005	0.013	-0.021, 0.030	0.016	0.007	0.001, 0.030
	1972	-0.011	0.018	-0.047, 0.024	0.017	0.010	-0.003, 0.038
	1973	-0.019	0.022	-0.062, 0.025	0.029	0.012	0.005, 0.053
	1974	-0.025	0.026	-0.077, 0.026	0.023	0.015	-0.006, 0.051
	1975	-0.028	0.030	-0.087, 0.031	0.011	0.017	-0.022, 0.043
	1976	-0.030	0.034	-0.097, 0.036	0.015	0.018	-0.021, 0.051
	1977	-0.026	0.039	-0.102, 0.050	0.009	0.020	-0.030, 0.049
	1978	-0.036	0.044	-0.122, 0.049	0.015	0.022	-0.028, 0.058
	1979	-0.057	0.051	-0.157, 0.044	0.038	0.025	-0.010, 0.086
Sibling Group Size	3 (ref)				0.000		
	4				-0.001	0.002	-0.005, 0.004
	5				0.012	0.004	0.004, 0.021
	6				0.007	0.007	-0.006, 0.021
	7				0.029	0.010	0.009, 0.049
N			108,024			108,024	

TABLE S14. Results: Relationship Between Preceding Birth Interval and Mortality for Swedish Men and Women Born 1938-1960.

		Within-family			Between-family		
		RR	SE	95% CI	RR	SE	95% CI
Preceding Interval Length	9-12	1.01	0.06	0.90-1.14	0.97	0.04	0.89-1.05
	13-18	0.96	0.03	0.91-1.02	0.94	0.02	0.91-0.98
	19-24	0.97	0.03	0.92-1.02	0.95	0.02	0.92-0.98
	25-30 (ref)	1.00			1.00		
	31-36	0.95	0.03	0.90-1.01	0.98	0.02	0.95-1.02
	37-42	0.99	0.03	0.93-1.05	1.00	0.02	0.96-1.04
	43-48	1.01	0.03	0.95-1.08	0.98	0.02	0.94-1.02
	49-54	1.04	0.04	0.96-1.11	1.01	0.02	0.97-1.06
	55-60	0.93	0.04	0.86-1.01	0.94	0.03	0.89-0.99
	61-66	0.99	0.04	0.91-1.08	1.00	0.03	0.94-1.05
	67-72	1.01	0.05	0.92-1.11	0.99	0.03	0.94-1.05
	73-78	0.94	0.05	0.84-1.04	0.97	0.03	0.91-1.04
	79-84	0.91	0.06	0.81-1.02	0.98	0.04	0.91-1.05
	85-90	0.89	0.07	0.78-1.02	0.93	0.04	0.85-1.01
	91-96	0.96	0.08	0.82-1.11	0.90	0.05	0.82-0.98
	97+	0.94	0.05	0.85-1.04	0.85	0.03	0.80-0.90
Gender	Men (ref)	1.00	0.00	1.00-1.00	1.00	0.00	1.00-1.00
	Women	0.65	0.01	0.63-0.67	0.68	0.01	0.67-0.70
Birth Order	2 (ref)	1.00	0.00	1.00-1.00	1.00	0.00	1.00-1.00
	3	1.03	0.02	0.99-1.07	0.91	0.01	0.89-0.93
	4	0.98	0.03	0.92-1.05	0.83	0.02	0.80-0.86
	5	1.00	0.05	0.91-1.10	0.82	0.03	0.78-0.86
	6	1.01	0.06	0.89-1.14	0.80	0.04	0.74-0.87
	7	1.04	0.08	0.89-1.21	0.73	0.04	0.67-0.80
Maternal Age	15-19	1.08	0.11	0.87-1.33	1.16	0.06	1.04-1.30
	20-24	1.01	0.03	0.95-1.07	1.04	0.01	1.01-1.07
	25-29 (ref)	1.00	0.00	1.00-1.00	1.00	0.00	1.00-1.00
	30-34	0.97	0.03	0.92-1.03	0.96	0.01	0.94-0.99
	35-39	0.97	0.04	0.89-1.06	0.95	0.01	0.93-0.98
	40-44	1.07	0.07	0.94-1.21	1.01	0.02	0.97-1.05
	45+	1.03	0.13	0.81-1.33	0.98	0.07	0.85-1.12
Cohort	1938-1940	1.03	0.04	0.94-1.12	0.88	0.02	0.85-0.91
	1941-1945	1.03	0.03	0.98-1.09	0.89	0.01	0.87-0.92
	1946-1950 (ref)	1.00	0.00	1.00-1.00	1.00	0.00	1.00-1.00
	1951-1955	0.98	0.03	0.93-1.04	1.26	0.02	1.22-1.30
	1956-1960	0.92	0.05	0.83-1.01	1.75	0.02	1.68-1.82
Sibling Group Size	3 (ref)				1.00	0.00	1.00-1.00
	4				0.83	0.01	0.81-0.84
	5				0.72	0.01	0.70-0.74
	6				0.64	0.02	0.62-0.66
	7				0.55	0.02	0.53-0.58
N			159,554			159,554	

TABLE S15. Results: Relationship Between Subsequent Birth Interval and Mortality for Swedish Men and Women Born 1938-1960.

		Within-family			Between-family		
		RR	SE	95% CI	RR	SE	95% CI
Subsequent	9-12	1.02	0.06	0.91-1.14	0.92	0.04	0.85-1.00
Interval Length	13-18	1.03	0.03	0.98-1.08	0.95	0.02	0.92-0.99
	19-24	1.03	0.03	0.98-1.08	0.98	0.02	0.95-1.02
	25-30 (ref)	1.00	0.00	1.00-1.00	1.00	0.00	1.00-1.00
	31-36	1.00	0.03	0.95-1.06	1.02	0.02	0.99-1.06
	37-42	1.02	0.03	0.96-1.09	1.03	0.02	0.99-1.07
	43-48	1.03	0.03	0.97-1.10	1.07	0.02	1.03-1.12
	49-54	0.98	0.04	0.91-1.05	1.05	0.02	1.00-1.09
	55-60	1.03	0.04	0.95-1.10	1.12	0.02	1.07-1.17
	61-66	1.00	0.04	0.93-1.09	1.13	0.03	1.08-1.19
	67-72	0.91	0.05	0.84-1.00	1.11	0.03	1.05-1.17
	73-78	0.96	0.05	0.88-1.06	1.13	0.03	1.06-1.19
	79-84	1.08	0.05	0.98-1.20	1.17	0.03	1.10-1.24
	85-90	1.09	0.06	0.98-1.23	1.18	0.03	1.11-1.27
	91-96	1.07	0.06	0.95-1.21	1.17	0.04	1.09-1.25
	97+	1.05	0.03	0.98-1.12	1.22	0.02	1.17-1.27
Gender	Men (ref)	1.00	0.00	1.00-1.00	1.00	0.00	1.00-1.00
	Women	0.64	0.01	0.62-0.66	0.69	0.01	0.68-0.70
Birth Order	1	1.01	0.02	0.97-1.05	1.15	0.01	1.12-1.18
	2 (ref)	1.00	0.00	1.00-1.00	1.00	0.00	1.00-1.00
	3	1.03	0.02	0.99-1.07	0.92	0.02	0.89-0.95
	4	1.00	0.04	0.93-1.07	0.81	0.02	0.78-0.85
	5	1.05	0.05	0.94-1.16	0.77	0.03	0.72-0.83
	6	1.07	0.07	0.93-1.23	0.72	0.05	0.65-0.80
Maternal Age	15-19	0.97	0.05	0.88-1.07	1.11	0.02	1.06-1.15
	20-24	0.99	0.02	0.94-1.04	1.05	0.01	1.03-1.07
	25-29 (ref)	1.00	0.00	1.00-1.00	1.00	0.00	1.00-1.00
	30-34	0.97	0.03	0.92-1.02	0.98	0.01	0.96-1.01
	35-39	0.97	0.05	0.89-1.07	1.01	0.02	0.97-1.04
	40-44	1.23	0.10	1.02-1.49	1.19	0.04	1.11-1.29
	45+	0.49	1.13	0.05-4.48	0.53	0.73	0.13-2.20
Cohort	1938-1940	1.12	0.04	1.03-1.21	0.71	0.01	0.69-0.73
	1941-1945	1.08	0.03	1.03-1.14	0.80	0.01	0.78-0.82
	1946-1950 (ref)	1.00	0.00	1.00-1.00	1.00	0.00	1.00-1.00
	1951-1955	0.95	0.03	0.90-1.01	1.46	0.02	1.41-1.50
	1956-1960	0.91	0.05	0.82-1.01	2.23	0.02	2.14-2.32
Sibling	3 (ref)				1.00	0.00	1.00-1.00
Group Size	4				0.82	0.01	0.80-0.84
	5				0.73	0.01	0.71-0.75
	6				0.66	0.02	0.64-0.68
	7				0.60	0.02	0.57-0.62
N			150,907			150,907	

TABLE S16. Preceding Birth Interval: Robustness Checks Using Alternative Specifications of Birth Interval Variable.

		Fitness				Height			
		Between-family		Within-family		Between-family		Within-family	
		Beta	95% CI	Beta	95% CI	Beta	95% CI	Beta	95% CI
Binary	0-24 months (ref)	0.000		0.00		0.000		0.000	
	>24 months	-0.051	-0.066, -0.036	0.011	-0.015, 0.036	-0.035	-0.050, -0.021	0.001	-0.019, 0.022
Binary	0-18 months (ref)	0.000		0.000		0.000		0.000	
	>18 months	-0.032	-0.053, -0.012	0.005	-0.028, 0.039	-0.019	-0.039, 0.001	0.007	-0.020, 0.035
Continuous	Interval (months)	-0.002	-0.002, -0.002	0.000	-0.001, 0.000	-0.001	-0.001, -0.001	0.000	0.000, 0.001
Quadratic	Interval (months)	-0.003	-0.005, -0.002	-0.003	-0.007, 0.001	-0.002	-0.003, -0.001	0.000	-0.004, 0.003
	Interval squared	0.000	0.000, 0.000	0.000	0.000, 0.000	0.000	0.000, 0.000	0.000	0.000, 0.000

		Underweight				Overweight			
		Between-family		Within-family		Between-family		Within-family	
		Beta	95% CI	Beta	95% CI	Beta	95% CI	Beta	95% CI
Binary	0-24 months (ref)	0.000		0.00		0.000		0.000	
	>24 months	0.000	-0.004, 0.004	0.005	-0.003, 0.012	0.016	0.011, 0.021	0.006	-0.003, 0.015
Binary	0-18 months (ref)	0.000		0.000		0.000		0.000	
	>18 months	-0.004	-0.010, 0.001	0.006	-0.004, 0.016	0.013	0.006, 0.019	0.009	-0.003, 0.021
Continuous	Interval (months)	0.000	0.000, 0.000	0.000	0.000, 0.000	0.001	0.001, 0.001	0.000	0.000, 0.001
Quadratic	Interval (months)	0.000	0.000, 0.000	0.000	-0.001, 0.002	0.000	0.000, 0.001	0.000	-0.002, 0.001
	Interval squared	0.000	0.000, 0.000	0.000	0.000, 0.000	0.000	0.000, 0.000	0.000	0.000, 0.000

		Mortality			
		Between-family		Within-family	
		RR	95% CI	RR	95% CI
Binary	0-24 months (ref)	1.00		1.00	
	>24 months	1.04	1.02-1.06	1.02	0.98-1.05
Binary	0-18 months (ref)	1.00		1.00	
	>18 months	1.03	1.00-1.06	1.01	0.97-1.05
Continuous	Interval (months)	1.00	1.00-1.00	1.00	1.00-1.00
Quadratic	Interval (months)	1.00	1.00-1.00	1.00	1.00-1.00
	Interval squared	1.00	1.00-1.00	1.00	1.00-1.00

TABLE S17. Subsequent Birth Interval: Robustness Checks Using Alternative Specifications of Birth Interval Variable.

		Fitness				Height			
		Between-family		Within-family		Between-family		Within-family	
		Beta	95% CI	Beta	95% CI	Beta	95% CI	Beta	95% CI
Binary	0-24 months (ref)	0.000		0.00		0.000		0.000	
	>24 months	-0.003	-0.018, 0.012	-0.011	-0.037, 0.016	-0.011	-0.026, 0.004	0.011	-0.011, 0.032
Binary	0-18 months (ref)	0.000		0.000		0.000		0.000	
	>18 months	0.019	-0.003, 0.041	-0.009	-0.044, 0.026	0.028	0.007, 0.050	0.024	-0.004, 0.052
Continuous	Interval (months)	0.000	0.000, 0.000	0.000	0.000, 0.000	0.000	0.000, 0.000	0.000	0.000, 0.000
Quadratic	Interval (months)	0.000	-0.001, 0.000	0.000	-0.001, 0.001	0.000	-0.001, 0.000	0.000	-0.001, 0.001
	Interval squared	0.000	0.000, 0.000	0.000	0.000, 0.000	0.000	0.000, 0.000	0.000	0.000, 0.000
		Underweight				Overweight			
		Between-family		Within-family		Between-family		Within-family	
		Beta	95% CI	Beta	95% CI	Beta	95% CI	Beta	95% CI
Binary	0-24 months (ref)	0.000		0.00		0.000		0.000	
	>24 months	0.001	-0.003, 0.005	-0.005	-0.013, 0.002	-0.001	-0.006, 0.004	0.005	-0.004, 0.014
Binary	0-18 months (ref)	0.000		0.000		0.000		0.000	
	>18 months	0.003	-0.003, 0.008	0.001	-0.009, 0.011	-0.007	-0.014, 0.000	0.000	-0.012, 0.012
Continuous	Interval (months)	0.000	0.000, 0.000	0.000	0.000, 0.000	0.000	0.000, 0.000	0.000	0.000, 0.000
Quadratic	Interval (months)	0.000	0.000, 0.000	0.000	-0.001, 0.000	0.000	0.000, 0.000	0.000	0.000, 0.001
	Interval squared	0.000	0.000, 0.000	0.000	0.000, 0.000	0.000	0.000, 0.000	0.000	0.000, 0.000
		Mortality							
		Between-family		Within-family					
		RR	95% CI	RR	95% CI				
Binary	0-24 months (ref)	1.00		1.00					
	>24 months	1.10	1.08-1.12	0.98	0.95-1.01				
Binary	0-18 months (ref)	1.00		1.00					
	>18 months	1.10	1.07-1.12	0.99	0.95-1.03				
Continuous	Interval (months)	1.00	1.00-1.00	1.00	1.00-1.00				
Quadratic	Interval (months)	1.00	1.00-1.00	1.00	1.00-1.00				
	Interval squared	1.00	1.00-1.00	1.00	1.00-1.00				

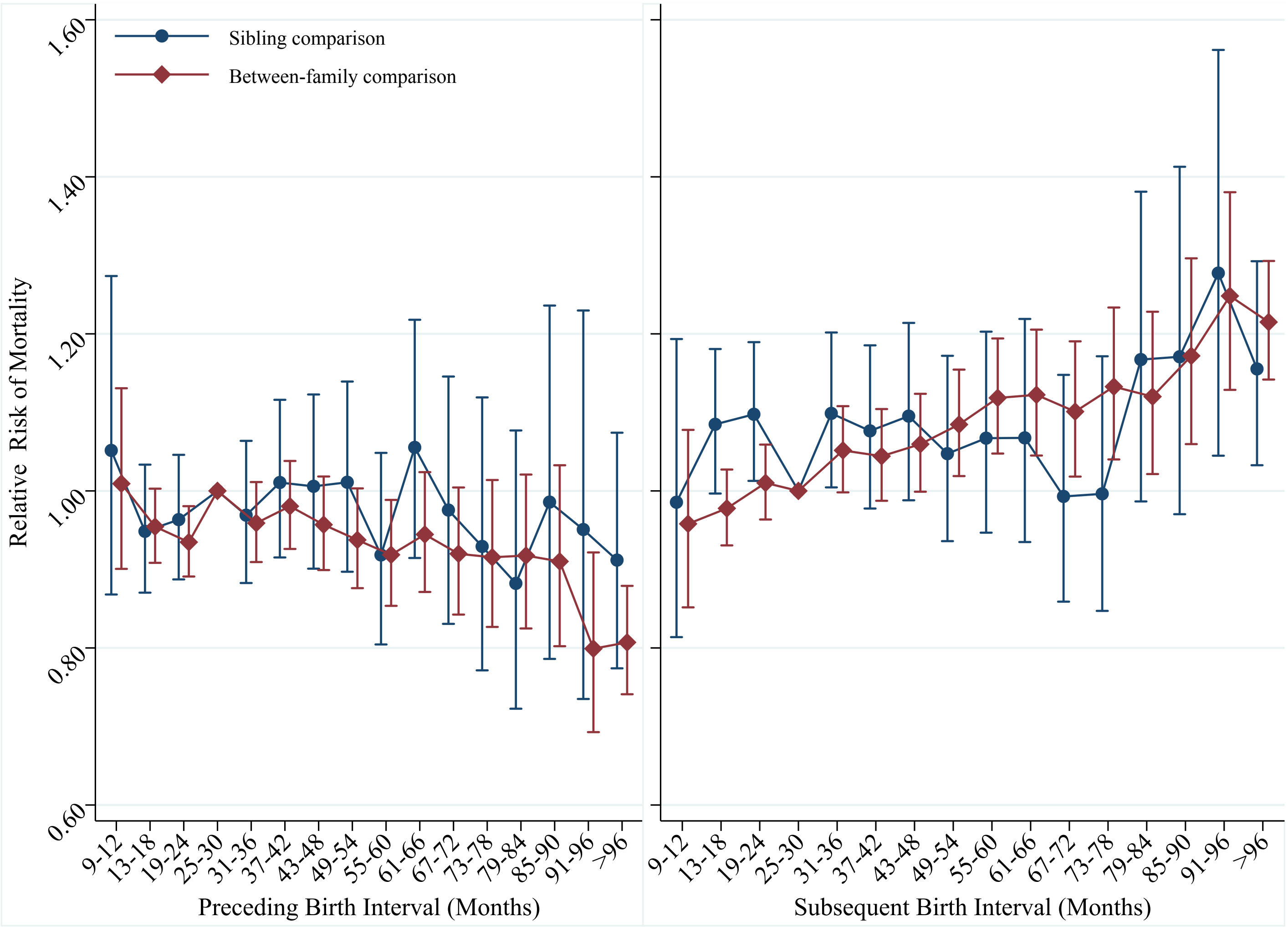

FIGURE S1. Hazard of mortality by preceding and subsequent birth intervals, Swedish men born 1938 to 1960. The analysis population for examining preceding birth intervals consists of individuals in sibling groups with at least three children, excluding the first-born. The analysis population for examining subsequent birth intervals consists of individuals in sibling groups with at least three children, excluding the last-born. Error bars are 95% confidence intervals.

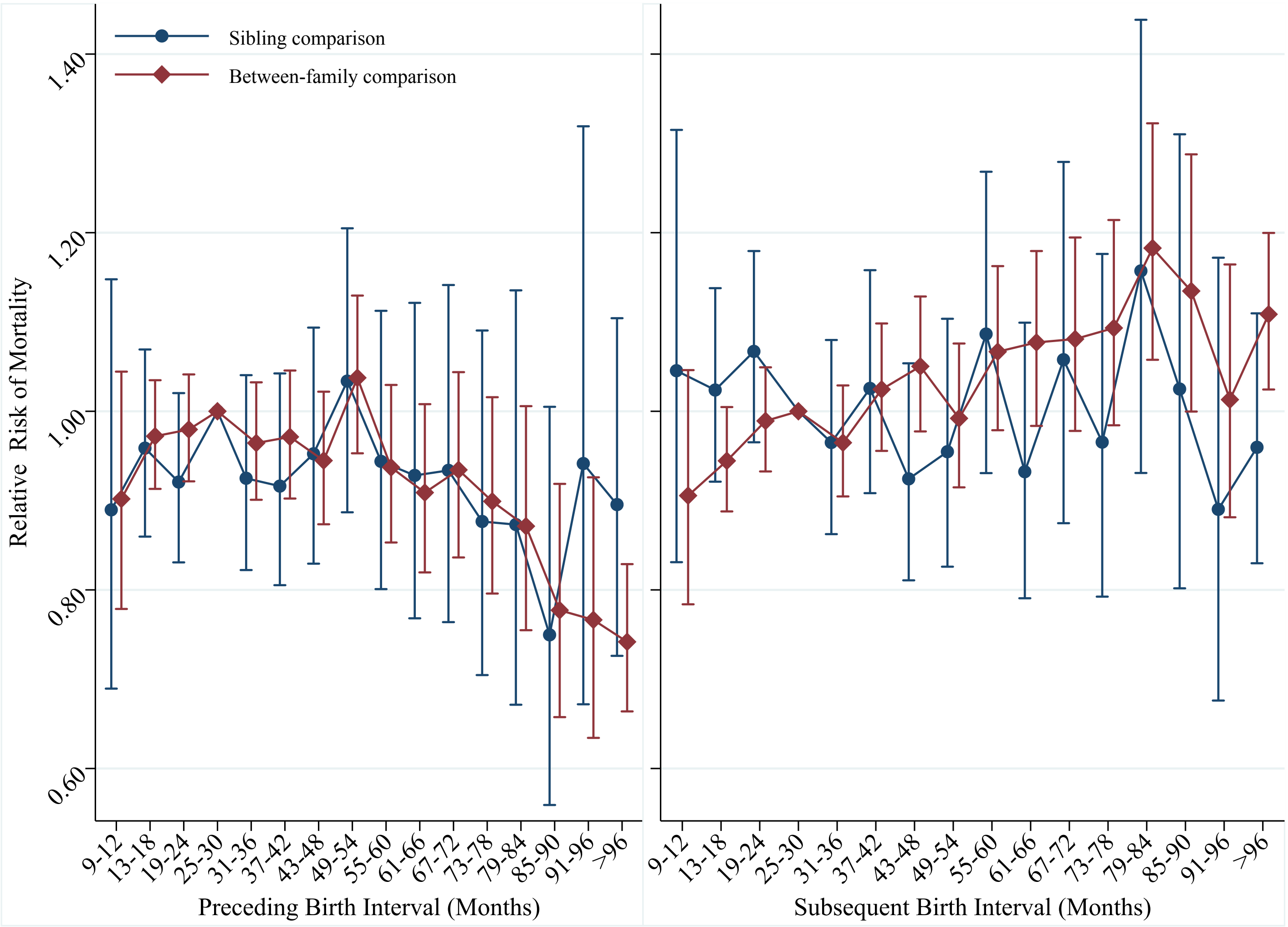

FIGURE S2. Hazard of mortality by preceding and subsequent birth intervals, Swedish women born 1938 to 1960. The analysis population for examining preceding birth intervals consists of individuals in sibling groups with at least three children, excluding the first-born. The analysis population for examining subsequent birth intervals consists of individuals in sibling groups with at least three children, excluding the last-born. Error bars are 95% confidence intervals.

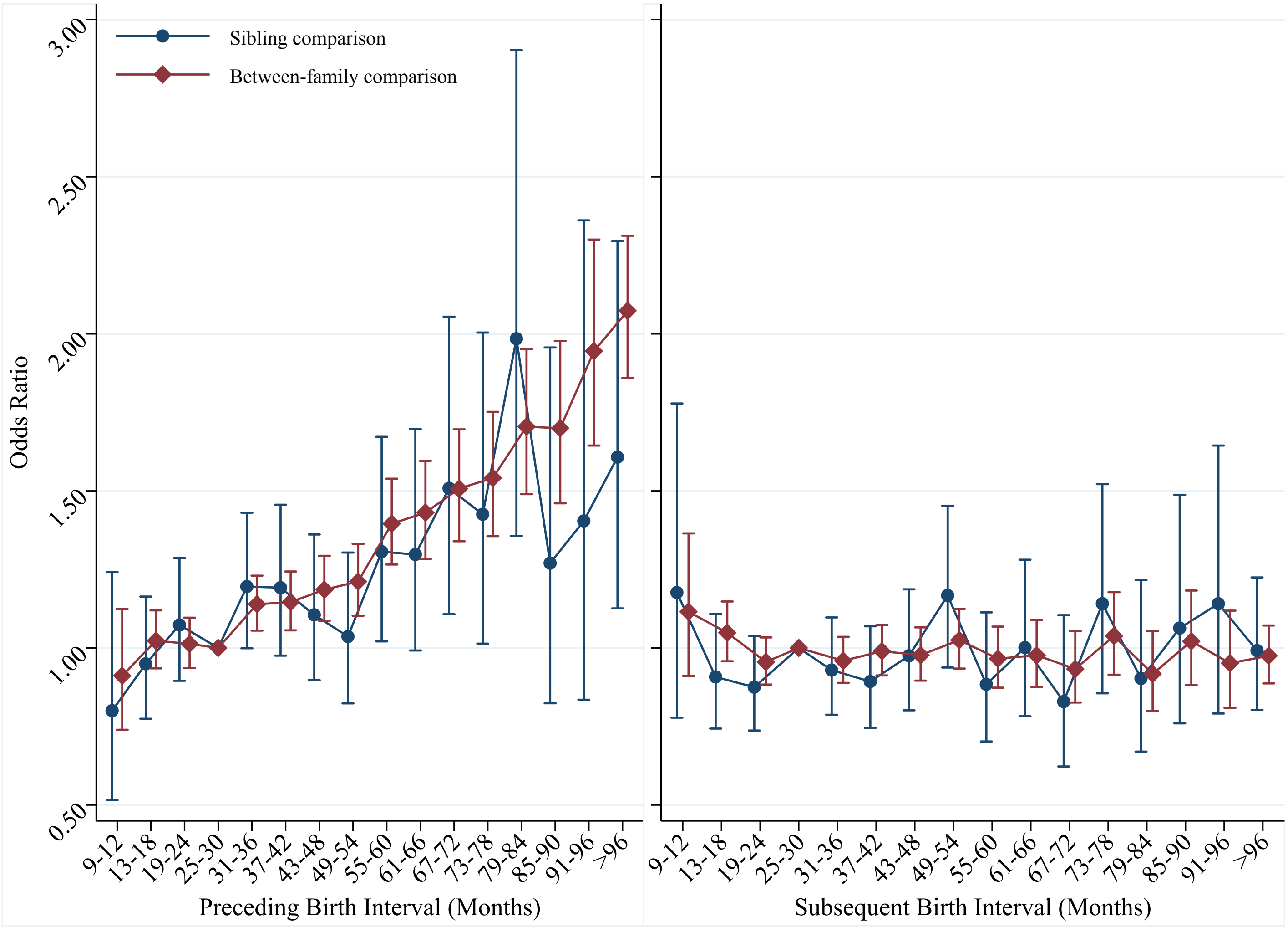

FIGURE S3. Odds ratios for being overweight or obese by preceding and subsequent birth intervals, Swedish men born 1962 to 1979. The analysis population for examining preceding birth intervals consists of individuals in sibling groups with at least three children, excluding the first-born. The analysis population for examining subsequent birth intervals consists of individuals in sibling groups with at least three male children, excluding the last-born. Error bars are 95% confidence intervals.

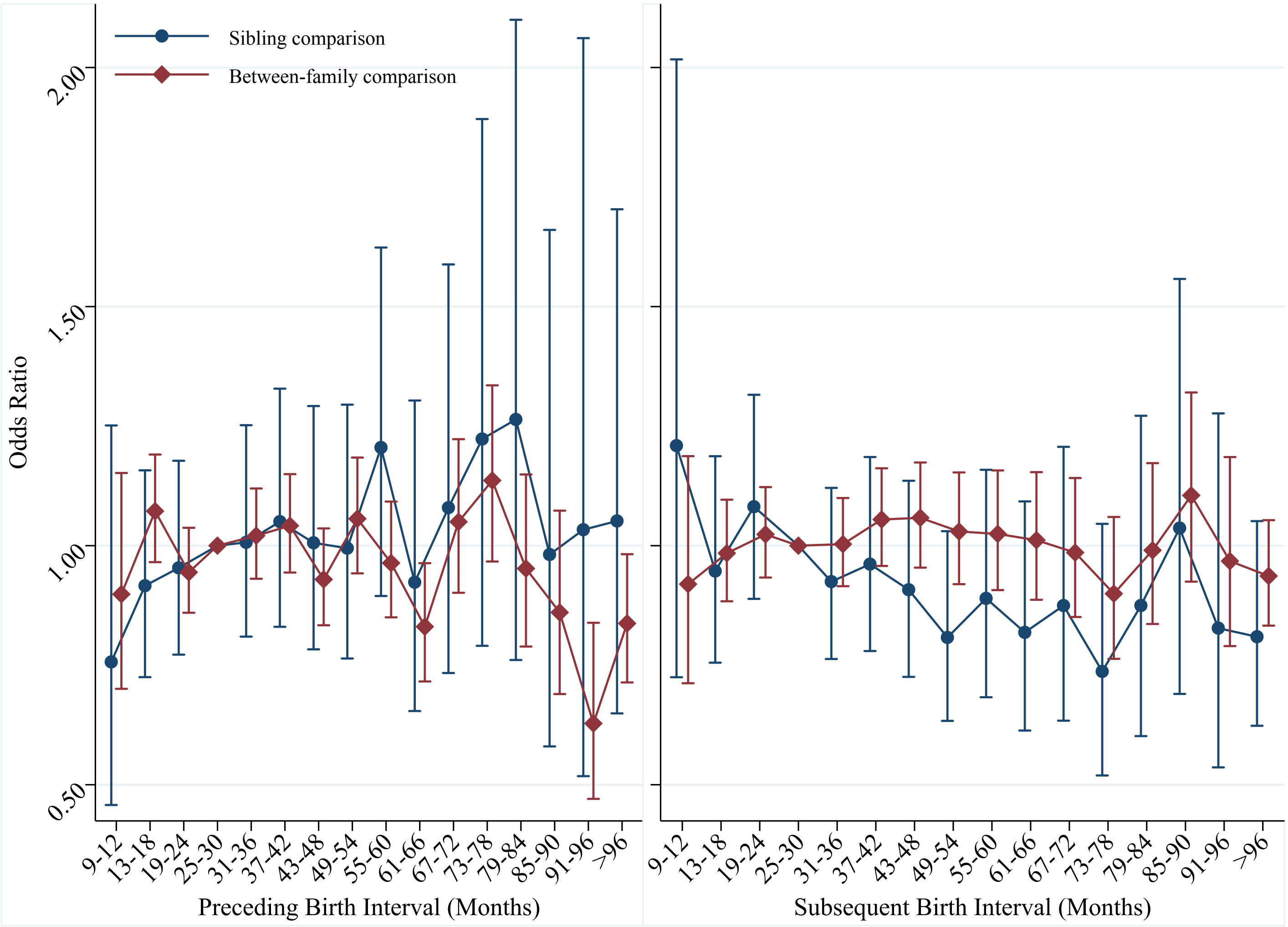

FIGURE S4. Odds ratios for being underweight by preceding and subsequent birth intervals, Swedish men born 1962 to 1979. The analysis population for examining preceding birth intervals consists of individuals in sibling groups with at least three children, excluding the first-born. The analysis population for examining subsequent birth intervals consists of individuals in sibling groups with at least three male children, excluding the last-born. Error bars are 95% confidence intervals.

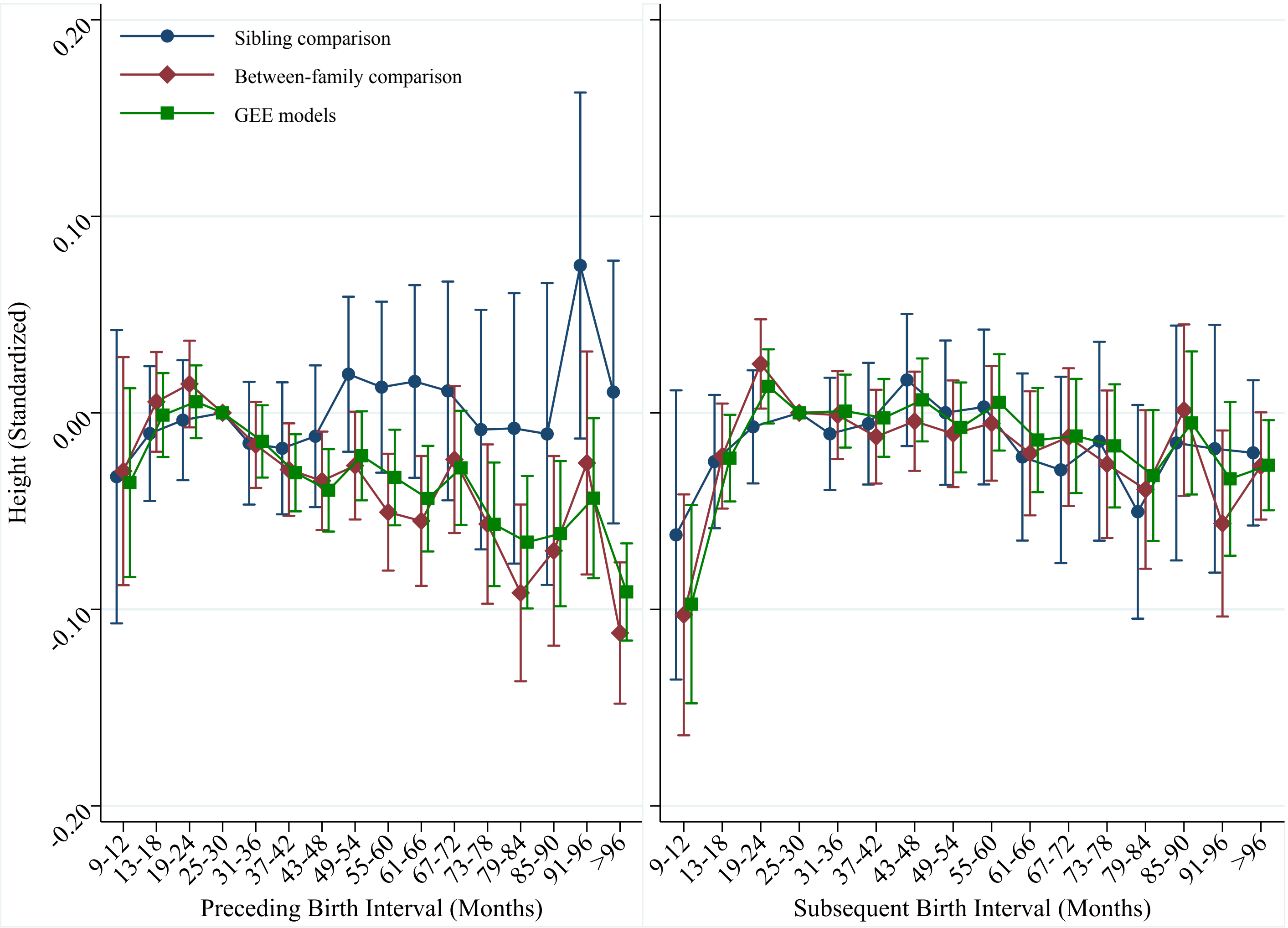

FIGURE S5. Height at ages 17 to 20 by preceding and subsequent birth intervals, Swedish men born 1962 to 1979. The analysis population for examining preceding birth intervals consists of individuals in sibling groups with at least three children, excluding the first-born. The analysis population for examining subsequent birth intervals consists of individuals in sibling groups with at least three male children, excluding the last-born. Error bars are 95% confidence intervals.

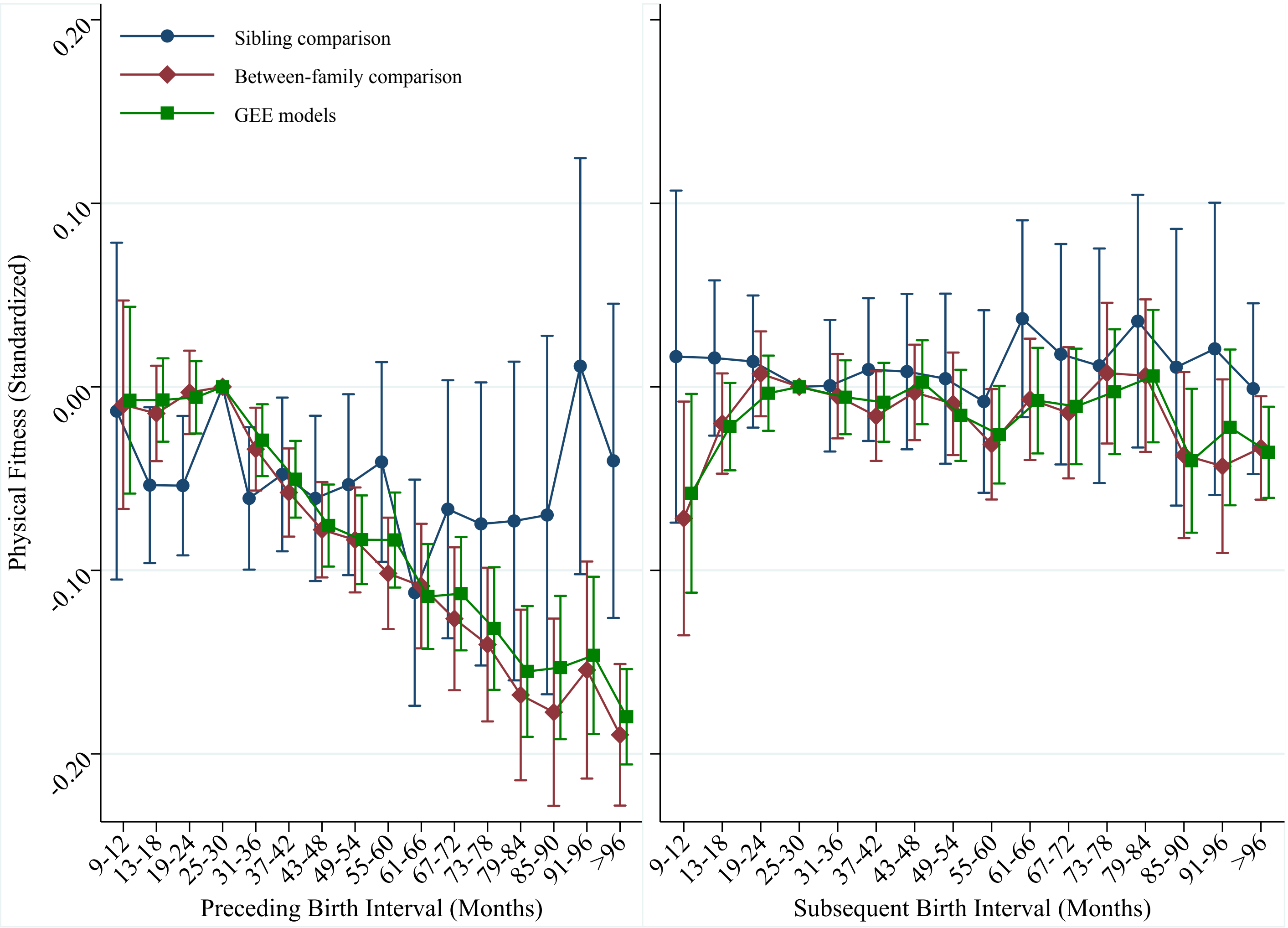

FIGURE S6. Physical fitness at ages 17 to 20 by preceding and subsequent birth intervals, Swedish men born 1962 to 1979. The analysis population for examining preceding birth intervals consists of individuals in sibling groups with at least three children, excluding the first-born. The analysis population for examining subsequent birth intervals consists of individuals in sibling groups with at least three male children, excluding the last-born. Error bars are 95% confidence intervals.

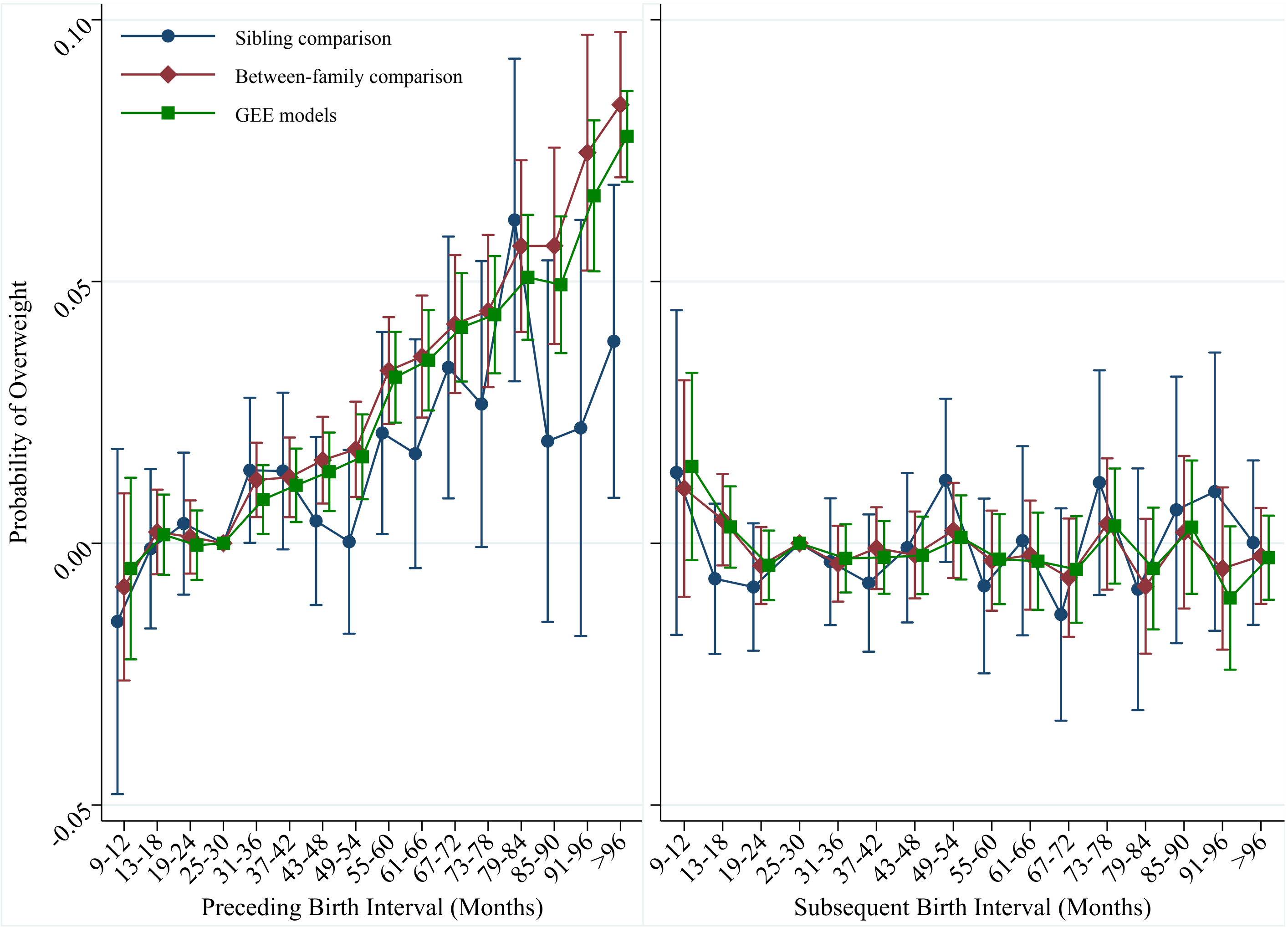

FIGURE S7. Predicted probability of being overweight or obese by preceding and subsequent birth intervals, Swedish men born 1962 to 1979. The analysis population for examining preceding birth intervals consists of individuals in sibling groups with at least three children, excluding the first-born. The analysis population for examining subsequent birth intervals consists of individuals in sibling groups with at least three male children, excluding the last-born. Error bars are 95% confidence intervals.

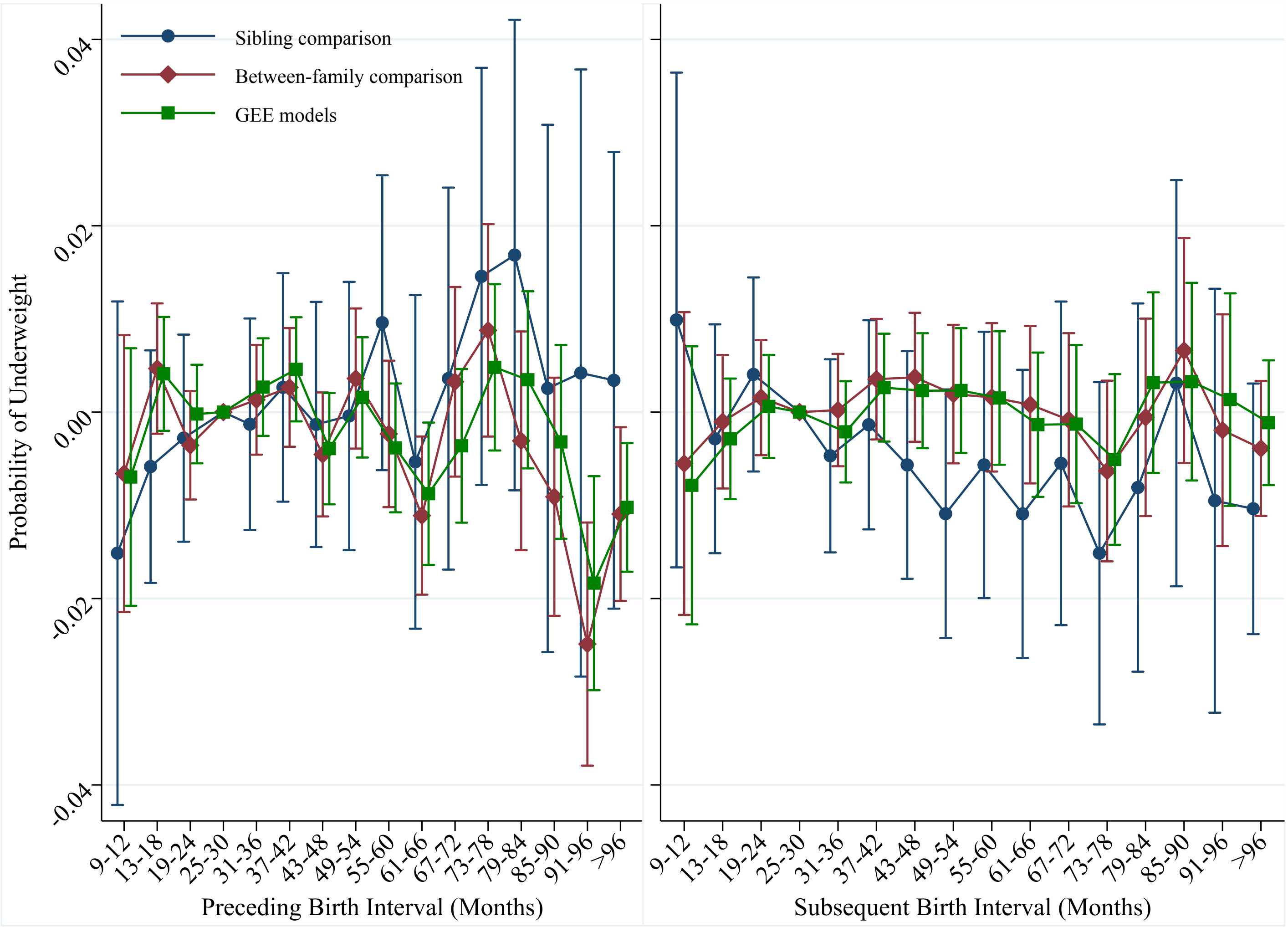

FIGURE S8. Predicted probability of being underweight by preceding and subsequent birth intervals, Swedish men born 1962 to 1979. The analysis population for examining preceding birth intervals consists of individuals in sibling groups with at least three children, excluding the first-born. The analysis population for examining subsequent birth intervals consists of individuals in sibling groups with at least three male children, excluding the last-born. Error bars are 95% confidence intervals.

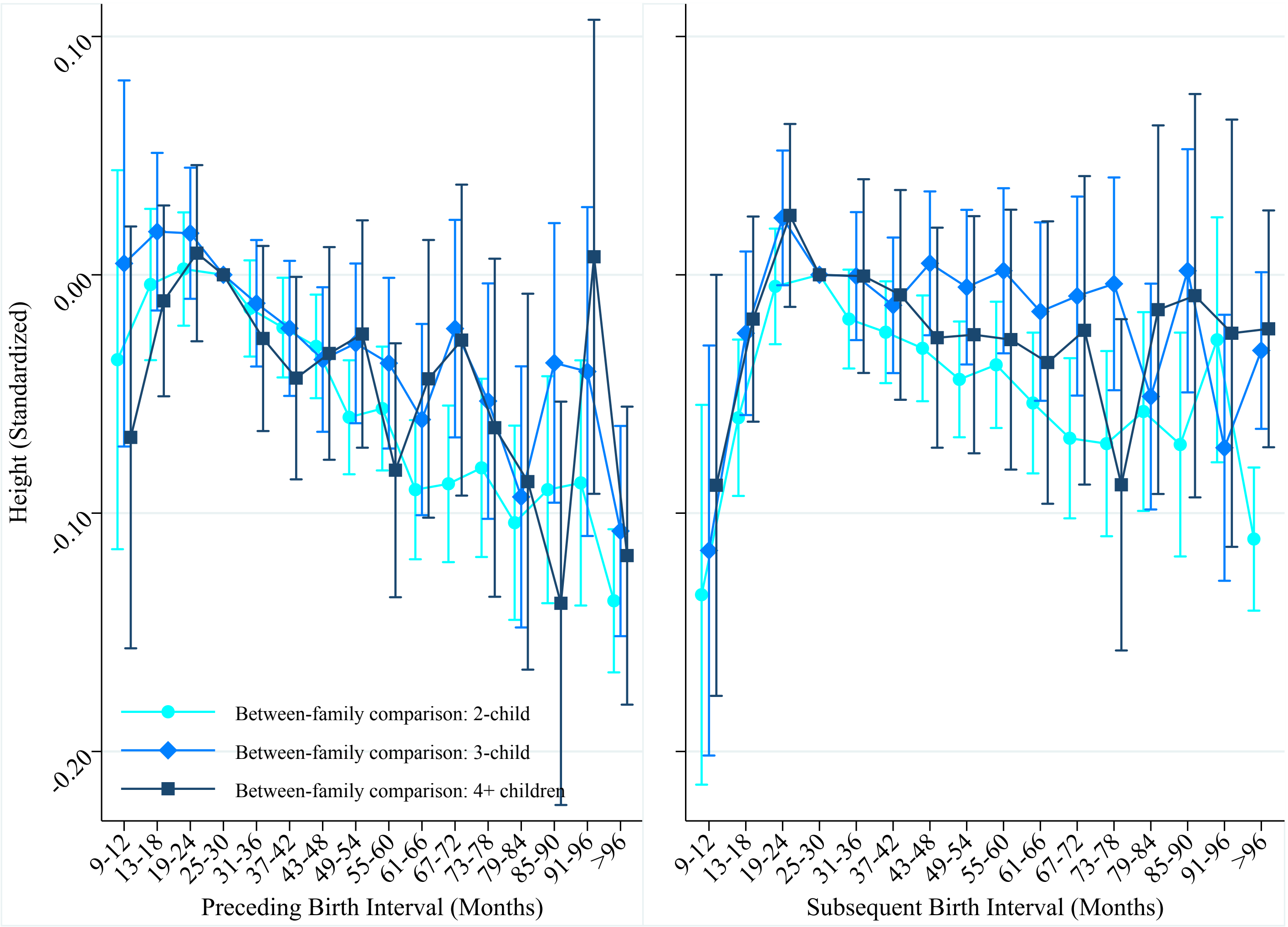

FIGURE S9. Height at ages 17 to 20 by preceding and subsequent birth intervals and by family size, Swedish men born 1962 to 1979. The analysis population for examining preceding birth intervals excludes first-borns. The analysis population for examining subsequent birth intervals excludes last-borns. Error bars are 95% confidence intervals.

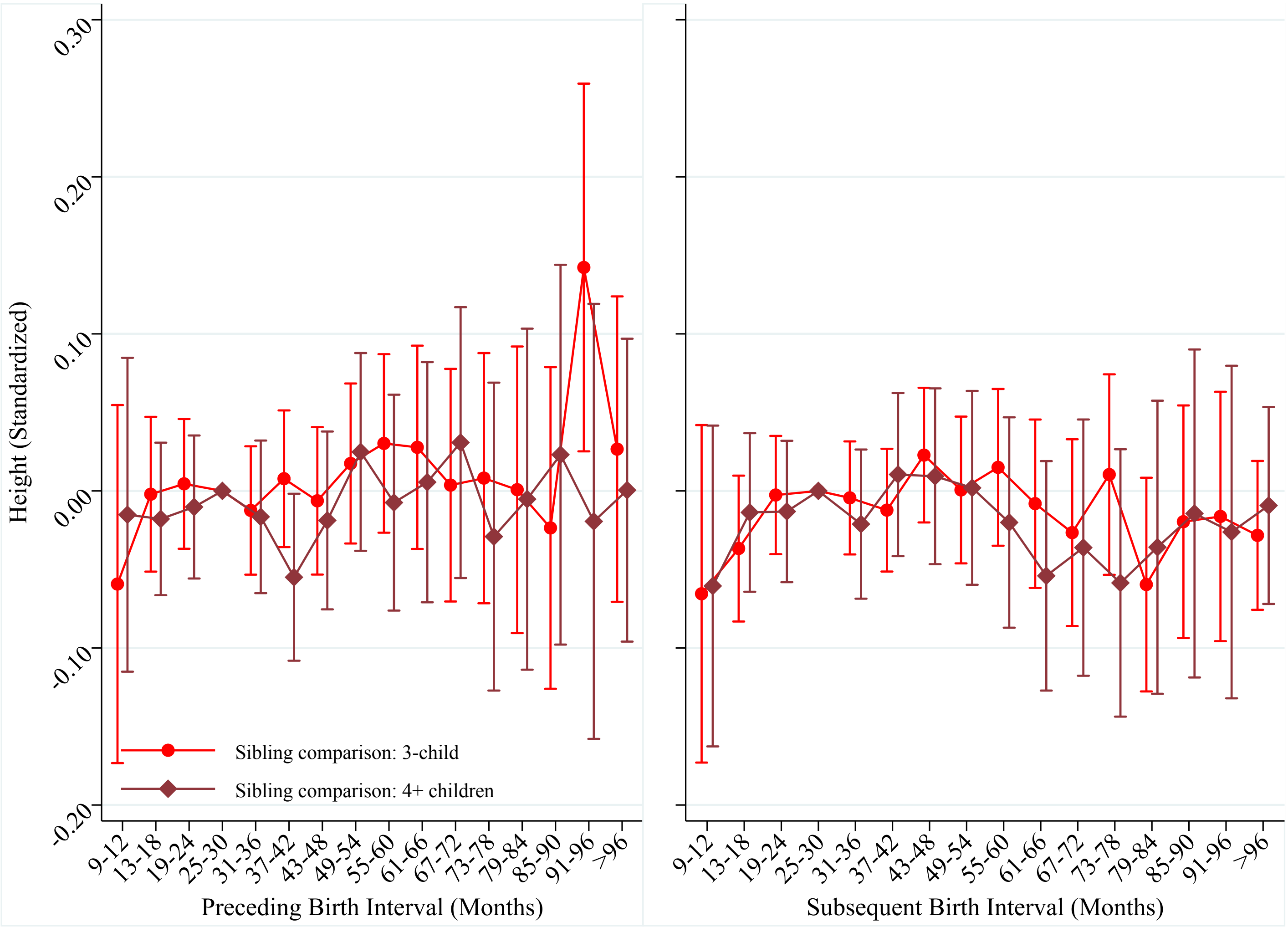

FIGURE S10. Height at ages 17 to 20 by preceding and subsequent birth intervals and by family size, Swedish men born 1962 to 1979. The analysis population for examining preceding birth intervals excludes first-borns. The analysis population for examining subsequent birth intervals excludes last-borns. Error bars are 95% confidence intervals.

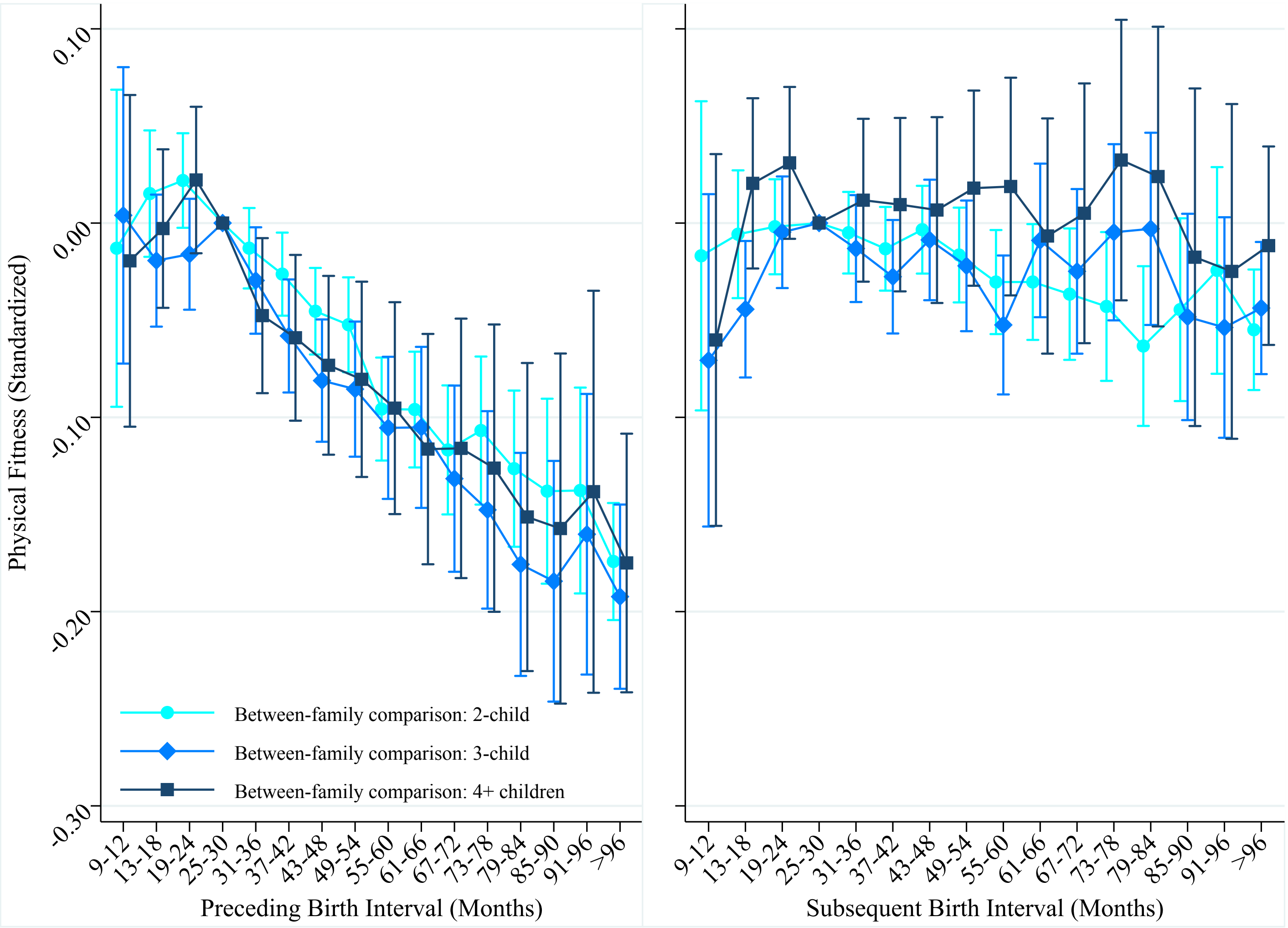

FIGURE S11. Physical fitness at ages 17 to 20 by preceding and subsequent birth intervals and by family size, Swedish men born 1962 to 1979. The analysis population for examining preceding birth intervals excludes first-borns. The analysis population for examining subsequent birth intervals excludes last-borns. Error bars are 95% confidence intervals.

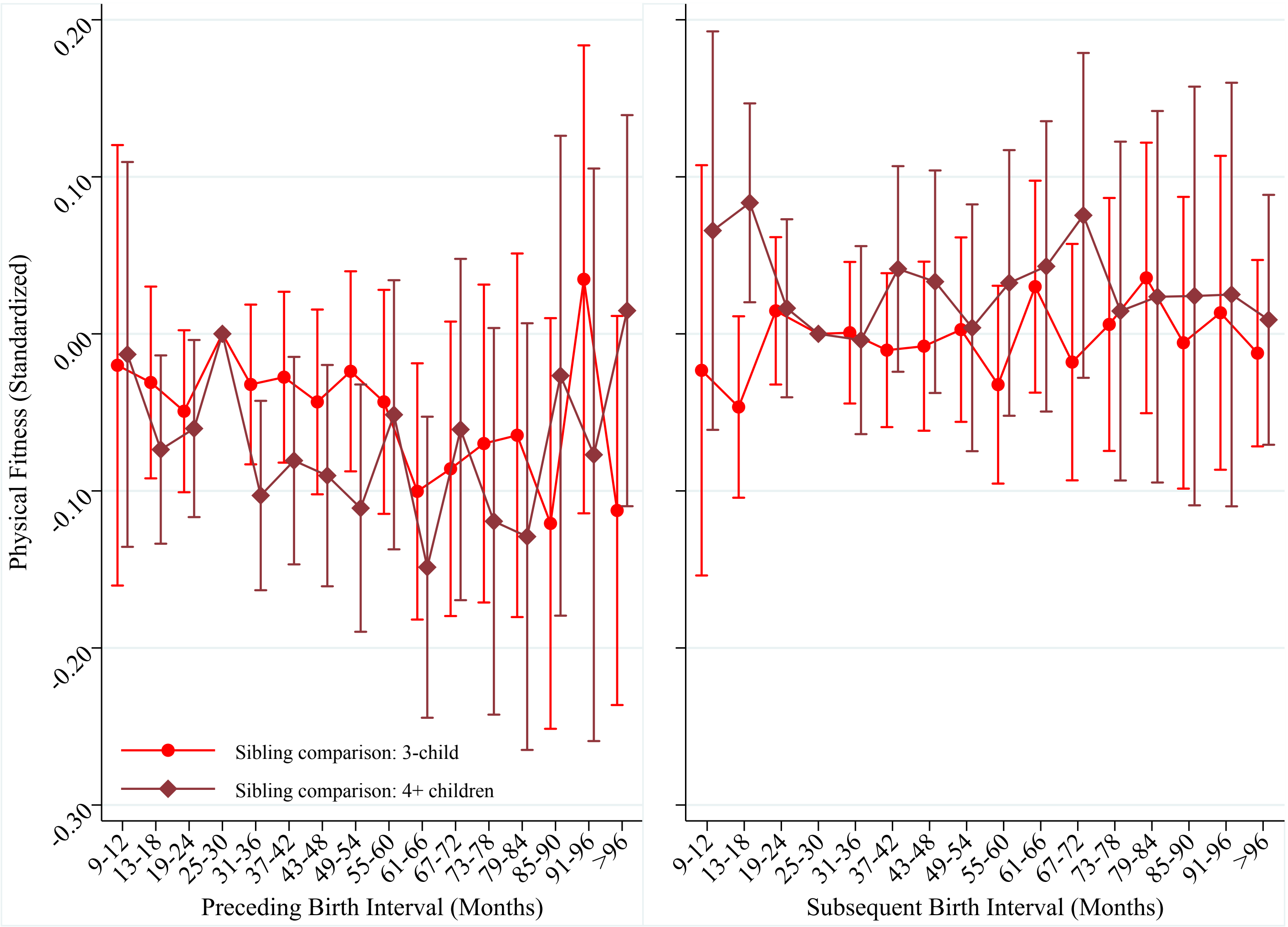

FIGURE S12. Physical fitness at ages 17 to 20 by preceding and subsequent birth intervals and by family size, Swedish men born 1962 to 1979. The analysis population for examining preceding birth intervals excludes first-borns. The analysis population for examining subsequent birth intervals excludes last-borns. Error bars are 95% confidence intervals.

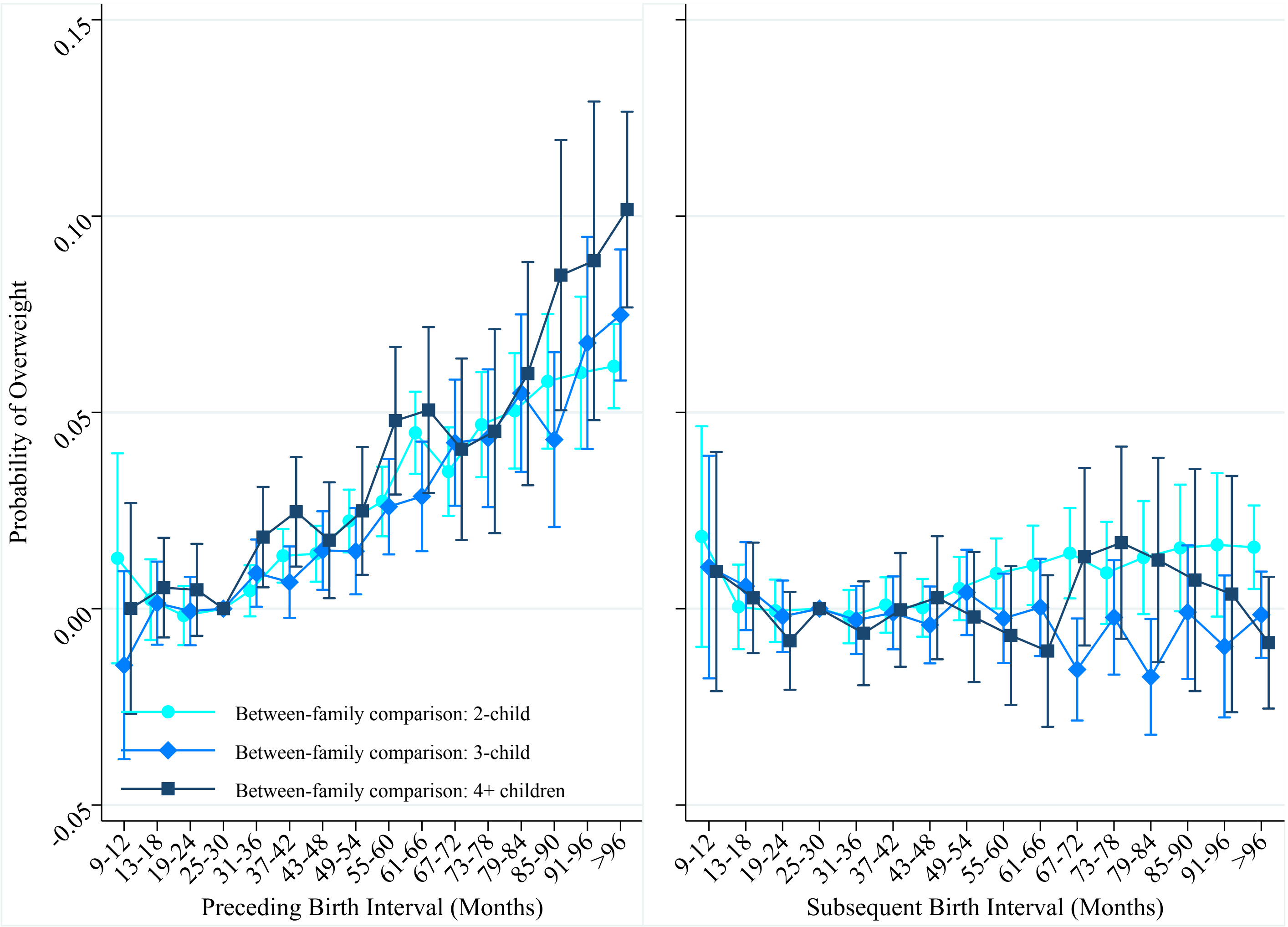

FIGURE S13. Predicted probability of being overweight or obese by preceding and subsequent birth intervals, Swedish men born 1962 to 1979. The analysis population for examining preceding birth intervals excludes first-borns. The analysis population for examining subsequent birth intervals excludes last-borns. Error bars are 95% confidence intervals.

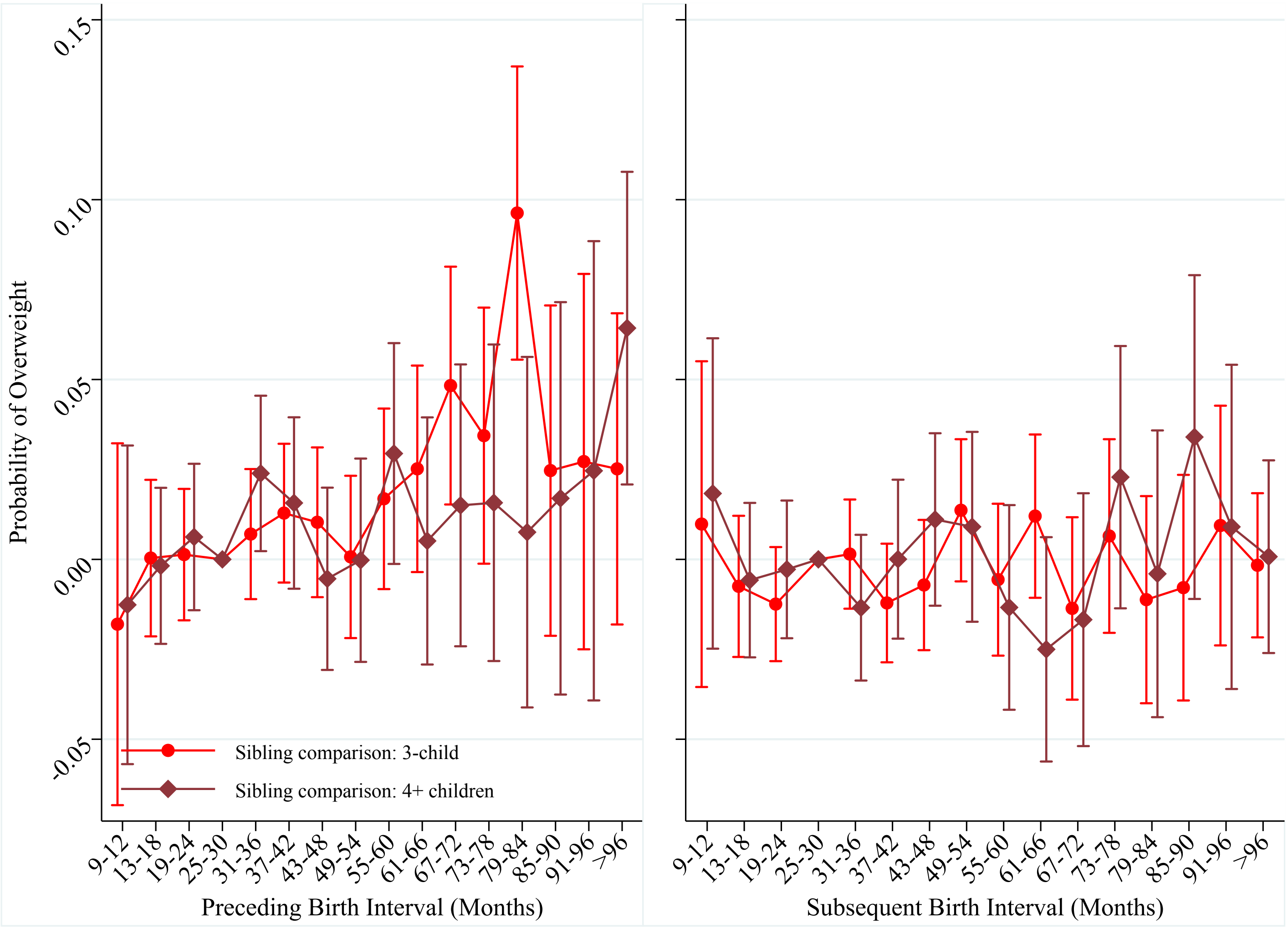

FIGURE S14. Predicted probability of being overweight or obese by preceding and subsequent birth intervals, Swedish men born 1962 to 1979. The analysis population for examining preceding birth intervals excludes first-borns. The analysis population for examining subsequent birth intervals excludes last-borns. Error bars are 95% confidence intervals.

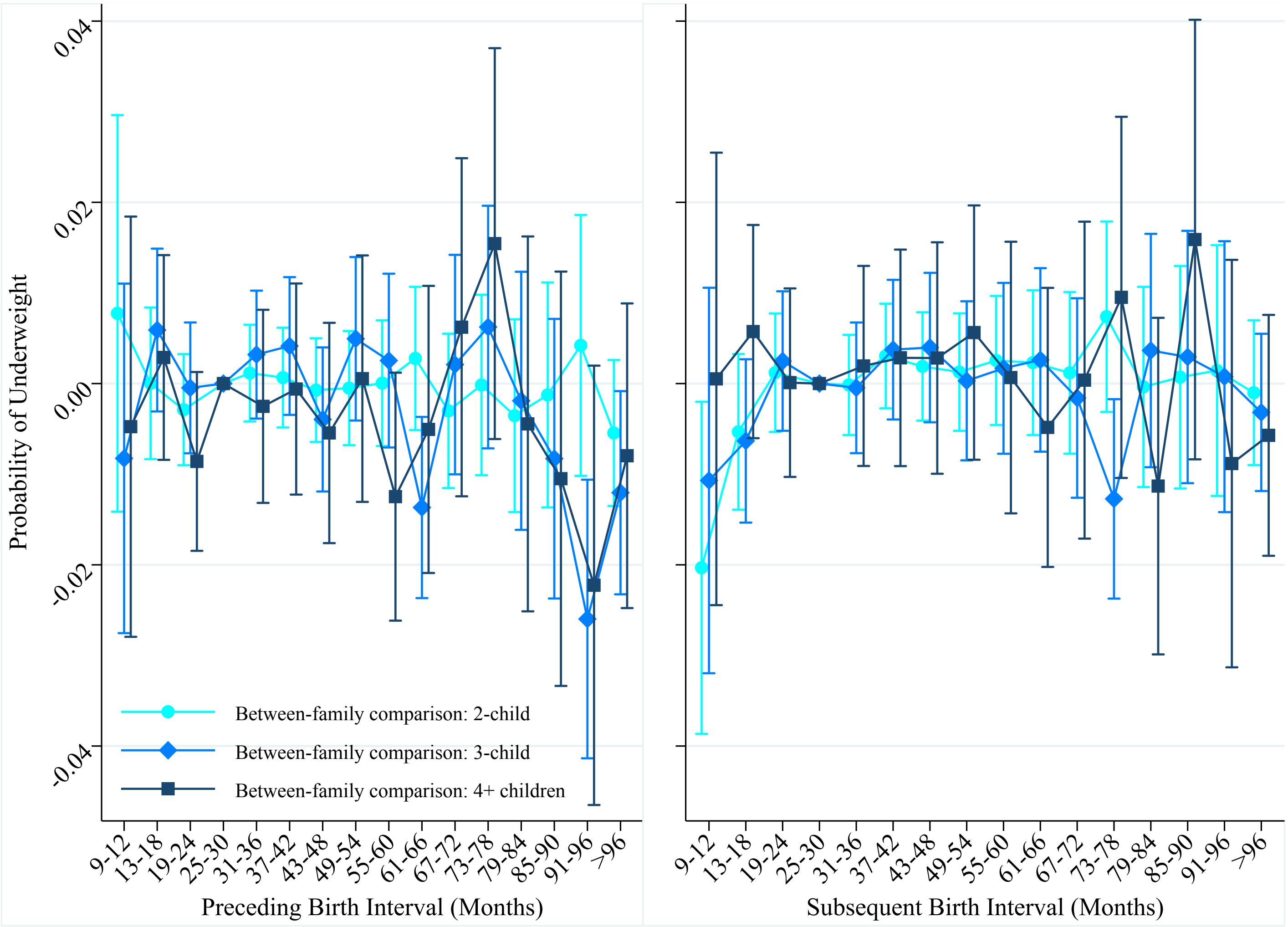

FIGURE S15. Predicted probability of being underweight by preceding and subsequent birth intervals, Swedish men born 1962 to 1979. The analysis population for examining preceding birth intervals excludes first-borns. The analysis population for examining subsequent birth intervals excludes last-borns. Error bars are 95% confidence intervals.

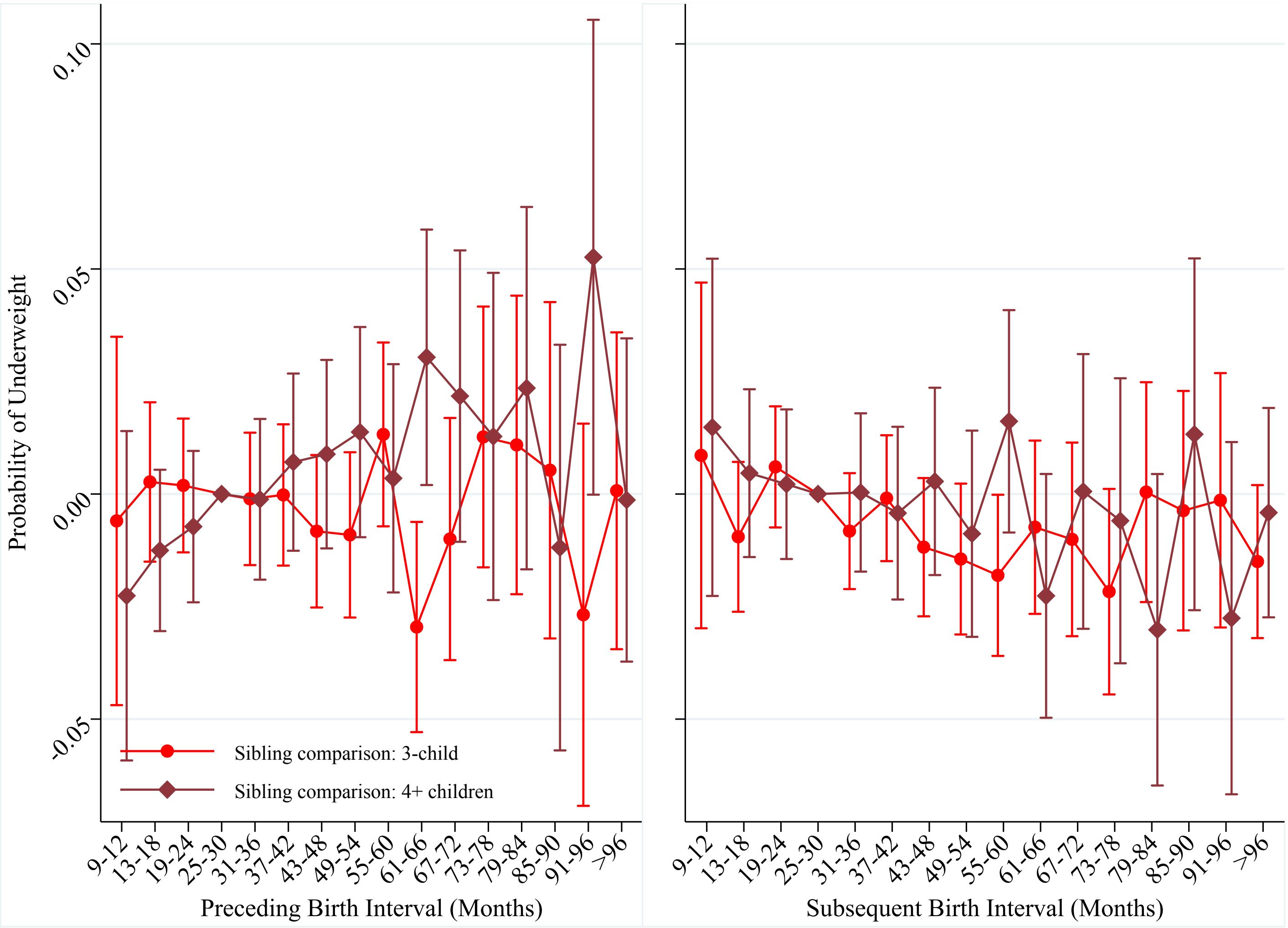

FIGURE S16. Predicted probability of being underweight by preceding and subsequent birth intervals, Swedish men born 1962 to 1979. The analysis population for examining preceding birth intervals excludes first-borns. The analysis population for examining subsequent birth intervals excludes last-borns. Error bars are 95% confidence intervals.

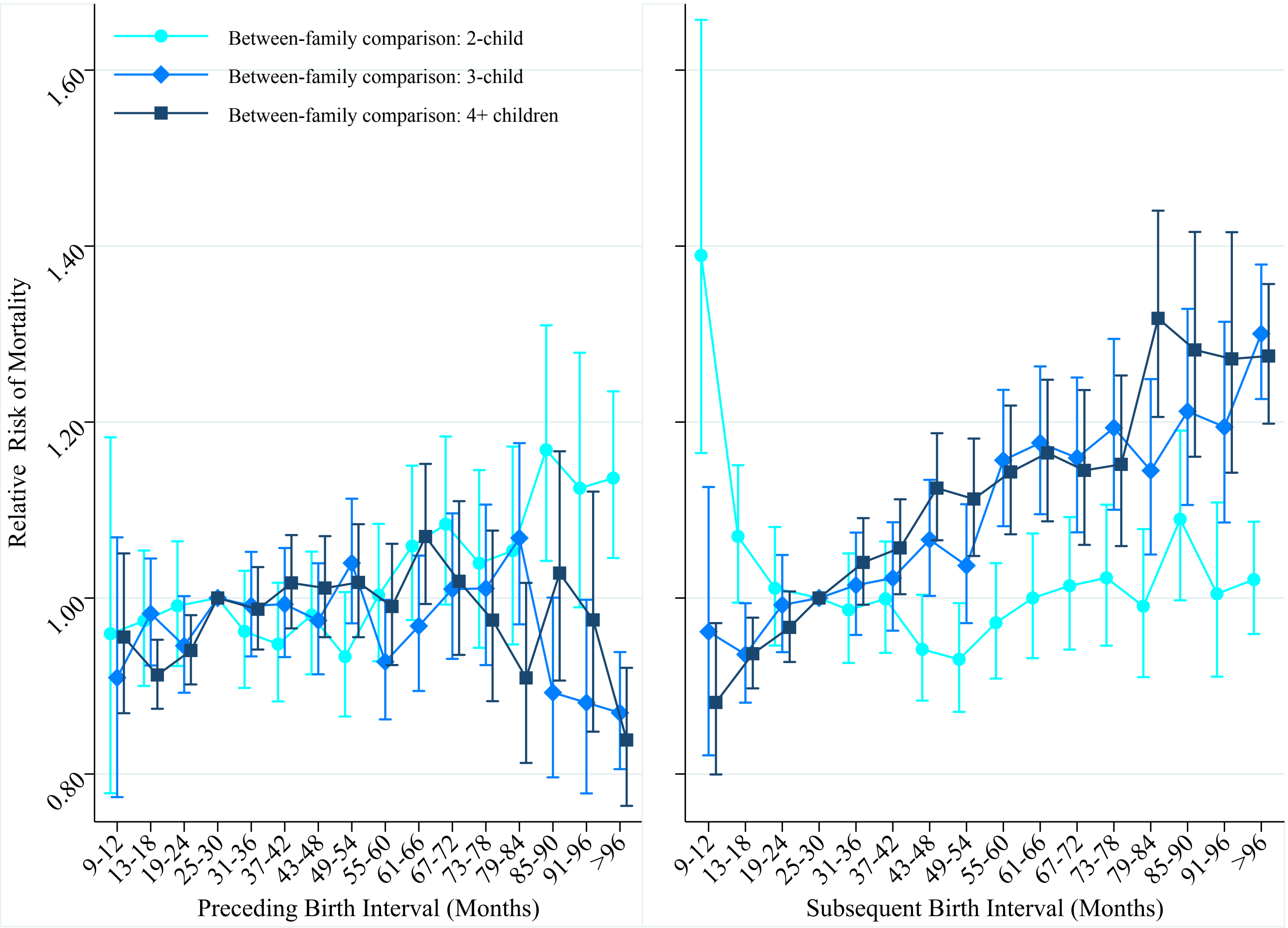

FIGURE S17. Hazard of mortality by preceding and subsequent birth intervals, Swedish men and women born 1938 to 1960. The analysis population for examining preceding birth intervals excludes first-borns. The analysis population for examining subsequent birth intervals excludes last-borns. Error bars are 95% confidence intervals.

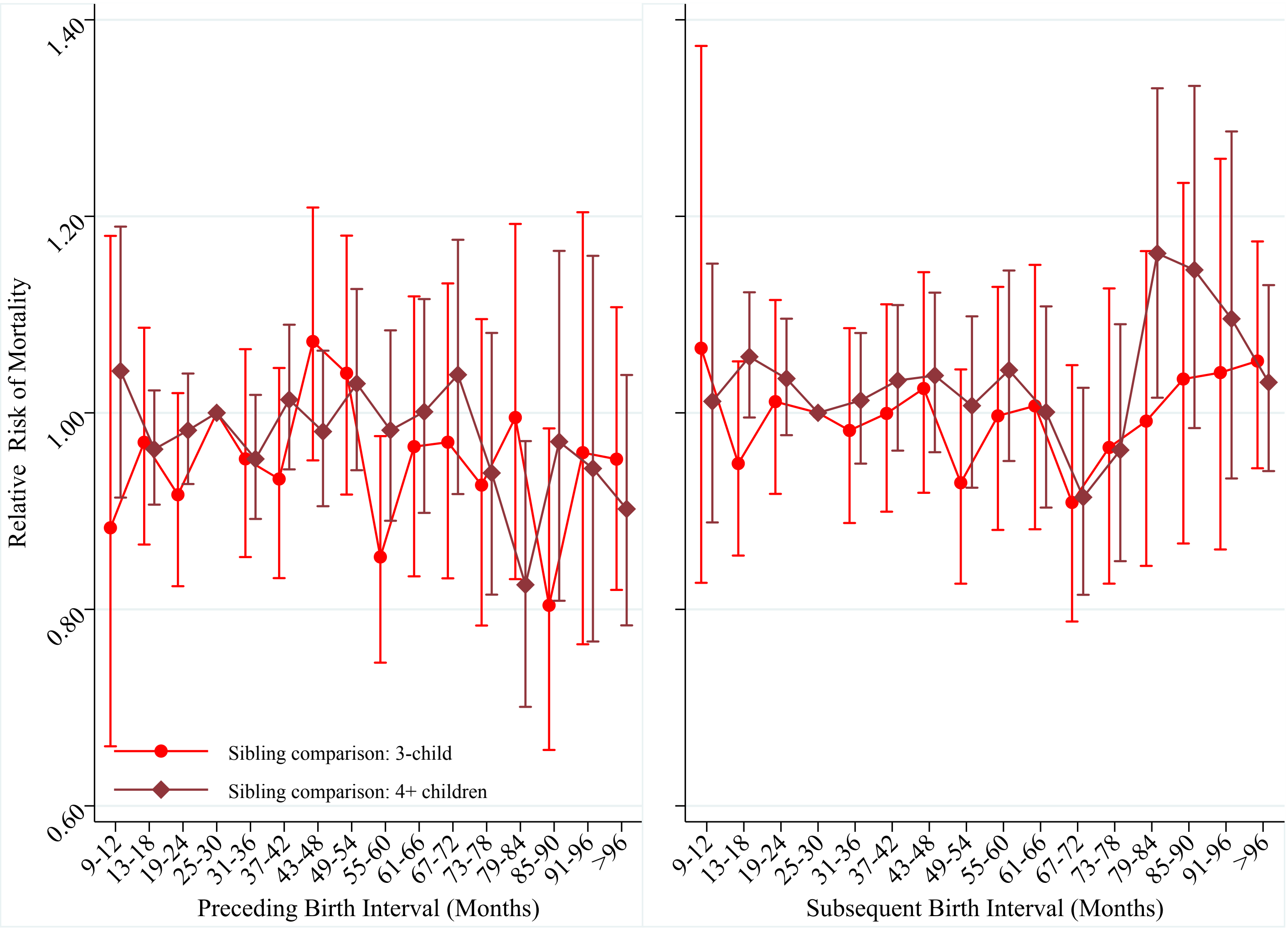

FIGURE S18. Hazard of mortality by preceding and subsequent birth intervals, Swedish men and women born 1938 to 1960. The analysis population for examining preceding birth intervals excludes first-borns. The analysis population for examining subsequent birth intervals excludes last-borns. Error bars are 95% confidence intervals.
